# Hybrid benzylidene thiazolidine-2,4-diones as potent apoptosis-inducing anticancer agents: design-driven optimization, cytotoxic profiling, and mechanistic validation in prostate cancer

**DOI:** 10.1039/d6ra01323f

**Published:** 2026-04-12

**Authors:** Saad Shaaban, Samia S. Hawas, Marwa Sharaky, Hussein Ba-Ghazal, Ayman Abo Elmaaty, Khadra B. Alomari, Mohamed Alaasar, Asma M. Elsharif, Fatema S. Alatawi, Mohamed Alaa Mohamed, Arwa Omar Al Khatib, Ahmed A. Al-Karmalawy

**Affiliations:** a Department of Chemistry, College of Science, King Faisal University Al-Ahsa 31982 Saudi Arabia sibrahim@kfu.edu.sa; b Department of Pharmaceutical Chemistry, Faculty of Pharmacy, Horus University-Egypt New Damietta 34518 Egypt akarmalawy@horus.edu.eg; c Cancer Biology Department, Pharmacology Unit, National Cancer Institute (NCI), Cairo University Cairo Egypt; d Medicinal Chemistry Department, Faculty of Pharmacy, Port Said University Port Said 42526 Egypt; e Medicinal Chemistry Department, Clinical Pharmacy Program, East Port Said National University Port Said 42526 Egypt; f Jazan University, Department of Physical Sciences, Chemistry Division 45142 Jazan Kingdom of Saudi Arabia; g Department of Chemistry, Faculty of Science, Cairo University Giza Egypt; h Department of Chemistry, College of Science, Imam Abdulrahman Bin Faisal University Dammam 31441 Saudi Arabia; i Department of Biochemistry, Faculty of Science, University of Tabuk Tabuk Saudi Arabia; j Department of Chemistry, Faculty of Science, Mansoura University Mansoura Egypt; k Faculty of Pharmacy, Al-Ahliyya Amman University Amman Jordan; l Department of Pharmaceutical Chemistry, College of Pharmacy, The University of Mashreq Baghdad 10023 Iraq

## Abstract

The smart combination of fragment merging, bioisosteric replacement, and conformational rigidification allowed for the design of a new series of *N*-(phenylthiazolyl)acetamide hybrids based on benzylidene-thiazolidine-2,4-dione as possible anticancer leads. Compounds with significantly increased cytotoxic activity were developed by methodically altering the benzylidene C4 ring structure and specifically modifying the thiazole olefinic bond. Analogues HB161 and HB162, which outperformed doxorubicin (DOX) against a variety of cancer types, demonstrated the highest average growth inhibition (GI) among the synthesized derivatives (HB121–HB169). A thorough IC_50_ analysis showed that HB123 and HB161 were more effective against PC-3 prostate cancer cells (7.14 and 7.90 µM, respectively), whereas HB162 showed balanced activity across colorectal, breast, and prostate cancer models. Strong apoptosis induction was demonstrated by mechanistic investigation, which showed that BAX and Caspase-3/7/9 were significantly upregulated while BCL-2, MMP-2, and MMP-9 were suppressed. Moreover, flow cytometry demonstrated that HB162-induced G0/G1 cell-cycle arrest is a major contributor to its antiproliferative action. To support HB161 and HB162 as BCL-2 downregulators, a molecular docking study was conducted focusing on the BCL-2 receptor, an essential component of the pathway that triggers apoptosis. Positive physicochemical behavior, non-mutagenicity, and good drug-likeness were highlighted by *in silico* ADMET profiling. In conclusion, this hybrid scaffold offers a promising platform for next-generation anticancer drugs that target apoptosis, especially for the treatment of prostate cancer.

## Abstract 2

Hybrid benzylidene thiazolidine-2,4-diones as potent apoptosis-inducing anticancer agents.

## Graphical abstract

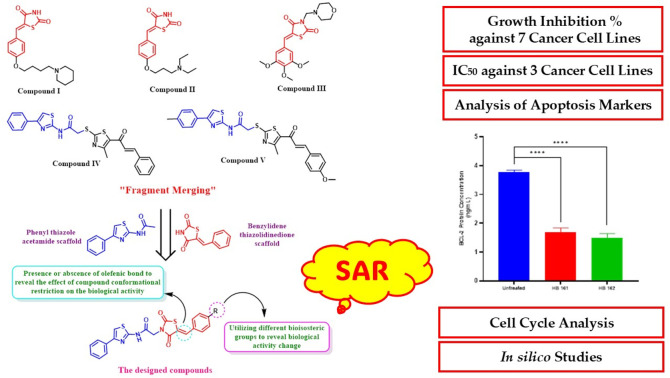

## 1. Introduction

Despite significant progress in cancer treatment, the disease continues to be a universal healthcare challenge.^1–3^ There were suggestions that global cancer cases could rise significantly, with estimates indicating an increase of more than 50% compared to current levels.^4,5^ Conventional anticancer therapies aim to kill rapidly dividing cells. However, this method also damages normal cells along with cancer cells. This can cause side effects like nausea, a weakened immune system, hair loss, and anemia.^6–8^

Two protein families play a key role in controlling apoptosis, or controlled cell destruction: BCL-2 and caspases. The caspase cascade is initiated by the release of cytochrome c. This procedure gets the cell ready to die.^9,10^ When activated, BAX, a crucial protein in the BCL-2 family that regulates apoptosis, causes mitochondrial outer membrane permeabilization (MOMP).^11,12^ This pathway is essential as it facilitates the release of cytochrome c and other pro-apoptotic molecules, which start programmed cell death.^9^ Additionally, p53 is essential for limiting cell proliferation. The role of p53 as a central tumor suppressor is highlighted by the fact that it is found in the nucleus, where it controls gene expression and can trigger apoptosis when required.^13,14^ Cellular stressors like DNA damage can cause p53 to build up inside the nucleus. When activated as a tumor suppressor, it can exhibit two responses: stopping the cell cycle to allow for DNA repair and starting apoptosis to get rid of damaged cells when repair is not possible.^13,15^

A class of zinc-dependent endopeptidases known as matrix metalloproteinases (MMPs) is essential for breaking down elements of the basement membrane (BM) and extracellular matrix (ECM).^16,17^ Therefore, gelatin and collagens IV and V are the main targets of matrix metalloproteinases MMP-2 and MMP-9.^18,19^ Their increased activity in cancer breaks down these barriers, allowing tumor invasion and metastasis, and providing insight into how MMP-2 and MMP-9 affect the development of cancer.^20,21^

Defects in the apoptosis pathway are linked to decreased pro-apoptotic proteins and increased anti-apoptotic proteins, which encourage researchers to use these markers to assess the effectiveness and safety of anticancer medications.^22–24^

In addition to conventional chemotherapeutic strategies, 2,4-thiazolidinedione (2,4-TZD) derivatives have drawn increasing attention in recent years as promising scaffolds for anticancer drug discovery. Originally developed as insulin sensitizers targeting peroxisome proliferator-activated receptor gamma (PPARγ), structural diversification of the TZD core has revealed significant anticancer potential across multiple tumor models beyond its antidiabetic applications.^25,26^ Recent medicinal chemistry efforts have highlighted that 2,4-TZD derivatives can modulate key pathways involved in tumorigenesis, including cell proliferation, apoptosis, angiogenesis, and metastasis. Their structural flexibility—especially at the C-5 position—allows for strategic substitution and functionalization that can enhance target affinity, selectivity, and cytotoxic potency.^25^ Mechanistically, many 2,4-TZDs appear to influence cell cycle regulators (*e.g.*, p21, p27), apoptotic proteins (*e.g.*, BAD, p53), and oncogenic signaling pathways such as PI3K/AKT/mTOR and RAS/RAF/MEK/ERK, as well as downregulate growth factor receptors like EGFR and angiogenic factors such as VEGF.^25^ Several recent studies have demonstrated the effectiveness of TZD analogues against various cancer cell lines. For instance, novel 2,4-TZD derivatives have been shown to disrupt oncogenic protein–protein interactions, inhibit tubulin polymerization, and induce apoptosis and cell cycle arrest in colon cancer models.^27^ Other reports describe the synthesis of TZD-bearing hybrid molecules exhibiting potent growth inhibition that rivals or surpasses standard chemotherapeutics such as doxorubicin (DOX), underlining their potential as cytotoxic agents.^28^ In addition, TZD derivatives incorporating heterocyclic or arylidene moieties continue to show promising antiproliferative profiles in breast, lung, and hepatic cancer cell lines, further supporting the versatility of this scaffold in anticancer drug design.^29^ Collectively, these advancements underscore 2,4-thiazolidinediones as valuable pharmacophores in the ongoing search for new anticancer therapeutics, motivating continued exploration of their structure–activity relationships and molecular mechanisms of action.^25^

**Fig. 1:**
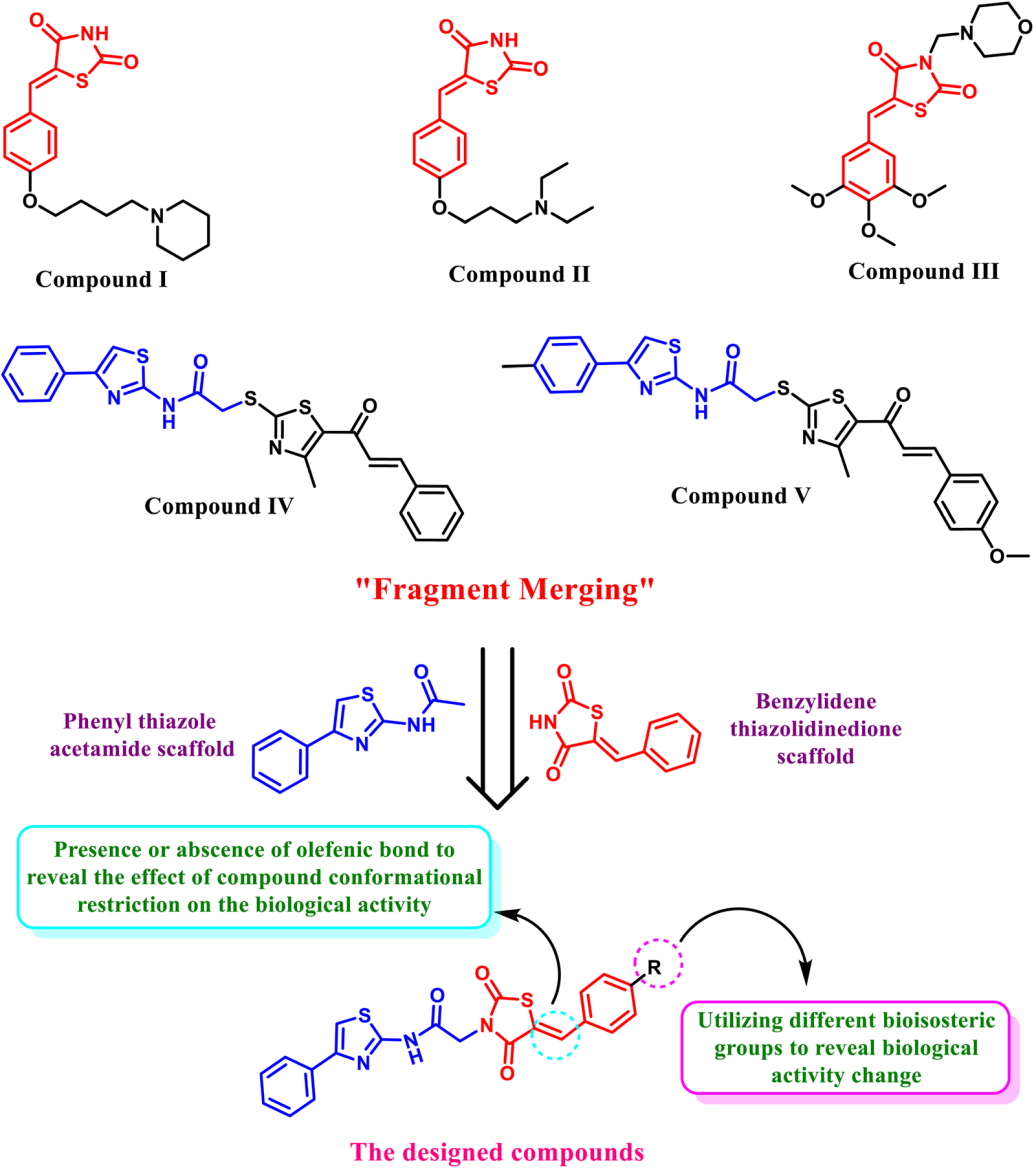
Some reported *N*-(phenylthiazole) acetamide/benzylidene thiazolidine-2,4-dione compounds with cytotoxic effect inspired the design of our new hybrid compounds through fragment merging, bioisosteric replacement, and structural rigidification.

The antitumor potential of *N*-(phenylthiazole) acetamide and benzylidene thiazolidine-2,4-dione scaffolds has been brought to light by efforts to find effective chemotherapeutics. In their study, Laxmi *et al.* developed benzylidene thiazolidine-2,4-dione analogues (compounds I and II, Fig. 1), which showed notable anticancer effects on a panel of 60 different types of cancer cells.^30^

The developed derivatives demonstrated significant efficacy against various cancer cell lines, including non-small cell lung cancer (NCI-H522), breast cancer (MDA-MB-468), prostate cancer (PC-3), colon cancer (COLO 205), and others.^30^ In addition, a series of benzylidene thiazolidine-2,4-dione derivatives (*e.g.*, compound III, Fig. 1) were synthesized by El-Kashef *et al.*, who subsequently evaluated their antitumor potential for breast cancer treatment.^31^ All synthesized compounds incorporated a thiazolidine-2,4-dione core conjugated with a trimethoxy-benzylidene group. Their anti-breast cancer activity was assessed using both normal breast cells and human breast cancer cells (MCF-7 and MDA-MB-231).^31^

Moreover, Al-Wahaibi *et al.* have afforded *N*-(phenylthiazole) acetamide derivatives as tubulin polymerization inhibitors endowed with apoptotic antiproliferative activity (*e.g.*, compounds IV and V).^32^ The MTT assay was used to examine the novel compounds' antiproliferative activity against HCT-116 colon cancer, PC-3 prostate cancer, and MCF-7 and MDA-MB-231 breast cancer cell lines. The results showed a significant cytotoxic effect when compared to reference medications, sorafenib, and DOX.^32^

Herein, a fragment merging approach was used to make benzylidene thiazolidine-2,4-dione and *N*-(phenylthiazole) acetamide containing hybrids to reveal their activity as new cytotoxic candidates. Moreover, the benzylidene ring was substituted at C4 with diverse bio-isosteric groups to pursue a change in cytotoxic activity. Additionally, the olefinic bond of the benzylidene moiety was replaced by a freely rotatable single bond for some derivatives to highlight the effect of conformation restriction of the designed compounds on cytotoxic activity.

## 2. Results and discussion

### 2.1. Chemistry

**Scheme 1:**
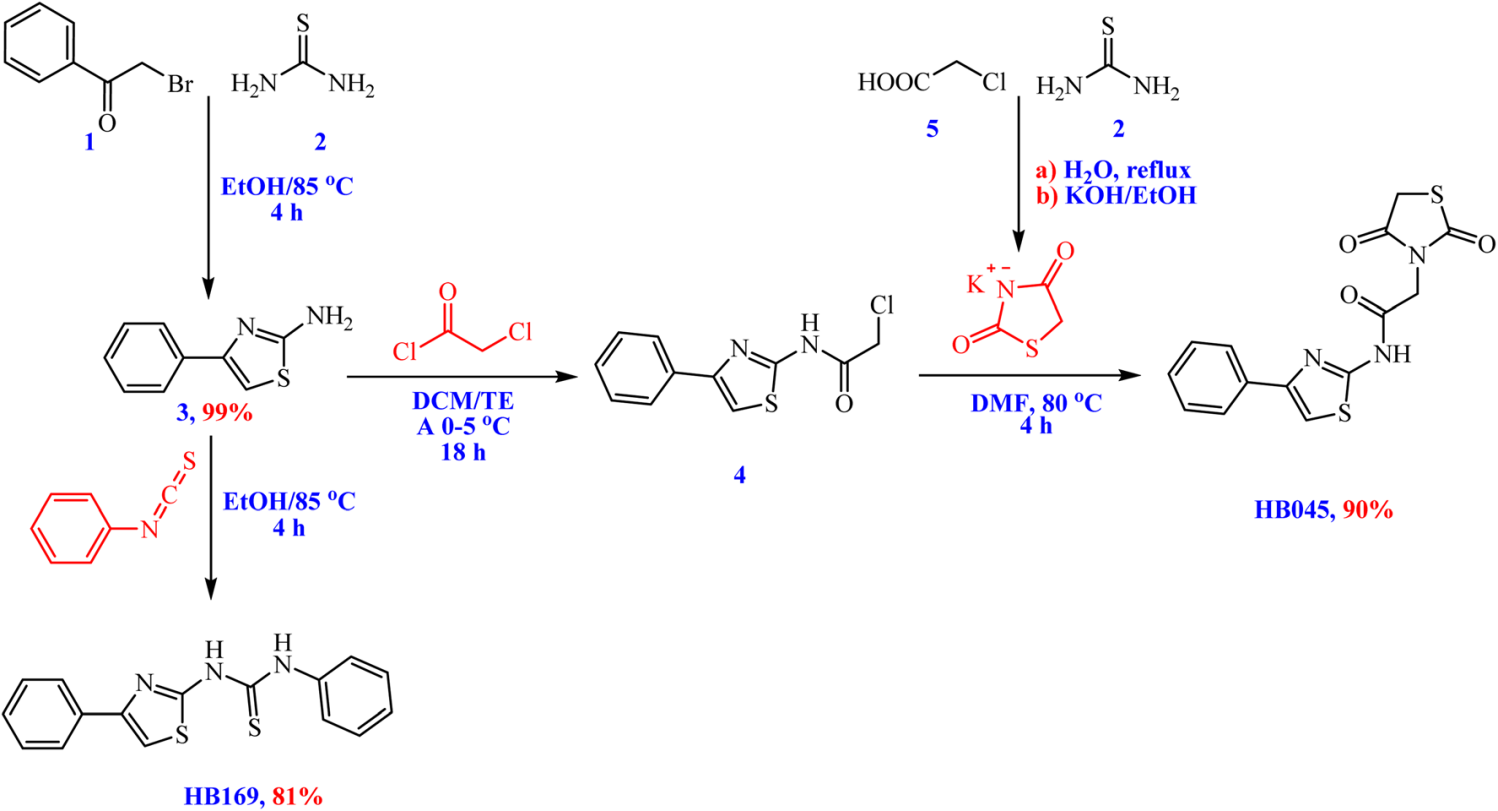
Synthesis of thiazolidinedione hybrids HB045 and HB169.

**Scheme 2:**
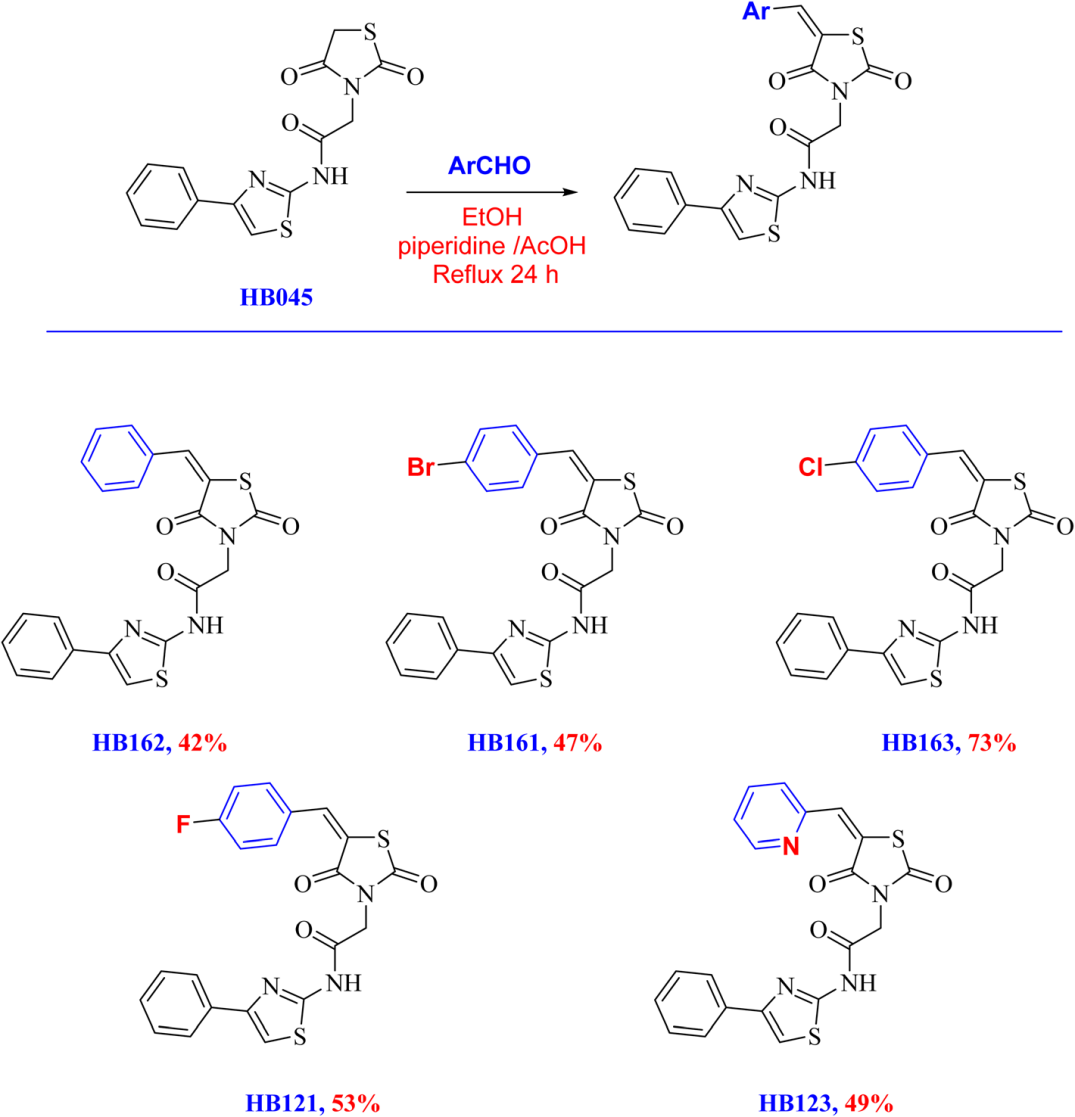
Synthesis of thiazolidinone-based arylidene derivatives *via* Knoevenagel condensation reactions.

The thiazole ring is a significant pharmacophore responsible for a variety of biological activities.^33–37^ They present as key structural components in numerous natural products, such as vitamin B_1_, and in pharmaceutical drugs, including the antidiabetic agents troglitazone and pioglitazone.^34,38,39^ Schemes 1 and 2 show the synthetic procedures utilized to create thiazolidinedione hybrids.

Thiazolidinedione hybrids were obtained *via* reaction of thiourea and phenacyl bromide, which furnished 4-phenylthiazol-2-amine (3) in 99% yield (Scheme 1). Subsequent reaction of 2-amino-thiazol 3 with phenyl isothiocyanate afforded the 1-phenyl-3-(4-phenylthiazol-2-yl)thiourea (HB169) in 81% yield. Furthermore, the acylation of 2-amino-thiazol 3 with chloroacetyl chloride in dichloromethane afforded the corresponding acetamide (4), which, upon reaction with the K salt of thiazolidine-2,4-dione, provided 2-(2,4-dioxothiazolidin-3-yl)-*N*-(4-phenylthiazol-2-yl)acetamide (HB045) in 90% yield (Scheme 1).

The FT-IR spectra of HB169 revealed significant bands at 1593–1493 cm-1 that corresponded to C=N and aromatic C=C stretching of the thiazole and phenyl moieties, along with a broad N–H stretching absorption at 3144 and 2936 cm^−1^, which is typical of the NH groups of thiourea. In the ^1^HNMR spectrum, two downfield singlets at *δ* 13.34 and 12.76 ppm correspond to the two hydrogen-bonded thiourea NH protons, confirming formation of the *N*,*N*′-disubstituted thiourea. The remaining signals appear between *δ* 8.68 and 7.15 ppm as multiplets and doublets integrating for eleven aromatic protons, in agreement with two phenyl rings and the thiazole proton. At *δ* 8.68 ppm, the most deshielded singlet can be attributed to the thiazole ring's H-5, which is typically shifted downfield by the adjacent heteroatoms, while the pattern of doublets, triplets, and multiplets between *δ* 8.04–7.15 ppm matches two monosubstituted phenyl groups. Signals at *δ* 178.26 and 163.15 ppm in the ^13^C NMR spectra can be referred to the thiocarbonyl (C=S) and C-2 of the thiazole ring, respectively. Signals in the range *δ* 139.63–124.01 ppm correspond to the aromatic carbons of both phenyl rings and the remaining thiazole carbons, while the resonance at *δ* 105.89 ppm is consistent with the thiazole C-5 carbon.

A broad N–H stretching band at 3224–3090 cm^−1^ in the FT-IR spectrum of HB045 is suggestive of the amide/thiazolidinedione NH functionality. Strong absorptions at 1759 and 1654 cm^−1^ are characteristic of the two carbonyl groups of the thiazolidinedione ring (imide-type C=O) and the amide C=O, respectively. The amide and thiazolidinedione fragments' N–H bending and C–N/C–O stretching vibrations are represented by extra bands at 1559, 1325–1275, and 1182 cm^−1^. The amide/thiazolidinedione NH proton was identified by a singlet signal at *δ* 12.68 ppm in the ^1^H NMR spectrum. The aromatic region (*δ* 7.88–7.32 ppm) shows one singlet and three sets of doublets/triplets integrating for five protons, in agreement with a monosubstituted phenyl ring and the thiazole proton. The methylene group tying the amide nitrogen to the thiazolidinedione ring and the thiazole-attached acetyl methylene are represented by two singlets at *δ* 4.47 and 4.32 ppm (2H each), respectively. In the ^13^C NMR spectrum, two carbonyl carbons of the thiazolidinedione ring appear at *δ* 172.01 and 171.51 ppm, while the amide carbonyl and thiazole C-2 resonate at *δ* 164.79 and 158.38 ppm, respectively. Aromatic and thiazole carbons are observed at *δ* 128.83, 127.92, 126.26, and 108.22 ppm. The two methylene carbons appear at *δ* 43.79 and 34.50 ppm, supporting the presence of distinct CH_2_ groups adjacent to heteroatoms (N and C=O).

The corresponding arylidene derivatives were then obtained by subjecting the thiazolidinedione–thiazole hybrid HB045 to the Knoevenagel condensation with a number of aromatic aldehydes, including benzaldehyde, 4-bromobenzaldehyde, 4-chlorobenzaldehyde, 4-fluorobenzaldehyde, and picolinaldehyde (Scheme 2). We assessed a variety of catalysts (AcOH, piperidine, and triethylamine) and solvents (polar protic, polar aprotic, and nonpolar) in our optimization investigations. These conditions, however, frequently resulted in a considerable recovery of the starting material but generally low conversions and product purity. However, utilizing ethanol as the solvent in conjunction with a catalytic mixture of piperidine and AcOH proved to be the most effective, allowing for complete conversion to the intended thiazolidinone-based arylidenes in fair to good yields (up to 42–73%).

Broad bands in the 3250–3380 cm^−1^ range were visible in the FT-IR for HB162, HB161, HB163, HB121, and HB123, which is consistent with the amide/thiazolidinedione N–H functionality. Strong absorptions between 1758–1738 and 1692–1654 cm^−1^ are attributed to the amide carbonyl and the two imide C=O groups of the thiazolidinedione ring. Additional bands at 1540–1560 cm^−1^ and 1610–1619 cm^−1^ are indicative of the thiazole ring and arylidene fragments' C=C/C=N stretching. The amide/thiazolidinedione NH was identified by a downfield singlet signal in the ^1^HNMR at *δ* ≈ 12.7 ppm. At *δ* ≈ 8.0 ppm, the olefinic proton of the 5 arylidene group was detected as a singlet. The methylene group (CH_2_CO) linker that connects the thiazolidinedione core to the thiazole acetamide is responsible for the singlet signal that all compounds displayed at *δ* ≈ 4.64–4.66 ppm (2H). All compounds showed three downfield carbonyl signals in the ^13^CNMR around *δ* ≈ 171–169 and 167–165 ppm, which correspond to the amide C=O of the acetamide chain and the two thiazolidinedione carbonyls. Additionally, a methylene (CH_2_) carbon at *δ* ≈ 44–46 ppm was present in all derivatives, which corresponds to the methylene group of the acetamide linker.

### 2.2. Evaluation of biology

#### 2.2.1. Determination of the growth inhibitory percentages (GI%) for derivatives HB121–HB169 through a panel of cancer and normal cell lines

To assess cytotoxic potential, compounds HB121–HB169 were tested for growth inhibition percentage (GI%) using the SRB colorimetric assay^40^ using seven human cancer cells; hepatocellular carcinoma (HuH-7), lung cancers (A549 and H460), human breast cancer (MCF-7), human osteosarcoma (MG63), colorectal carcinoma (HCT-116), and prostate cancer (PC-3).

To further evaluate the safety of the synthesized compounds in non-malignant cells, the human skin fibroblast (HSF) line was incorporated into the study. The GI% for analogues HB121–HB169 are presented in Table 1, with their efficacy compared against the reference anticancer drug (DOX).

**Table 1:** The GI% values for analogues (HB121–HB169) (±SD) were determined utilizing seven cancer and one normal cell lines

	HB121	HB123	HB161	HB162	HB163	HB169	DOX
H460	81.99	68.07	83.86	90.34	80.03	79.48	73.58
	±5.13	±2.73	±2.32	±6.66	±11.12	±1.97	±1.76
HCT-116	63.10	44.65	85.56	82.52	67.48	55.48	71.28
	±3.55	±8.90	±2.81	±5.75	±4.18	±5.45	±5.07
MCF7	58.27	47.80	79.70	81.28	42.35	70.17	69.00
	±9.17	±4.30	±6.57	±3.20	±4.92	±6.14	±0.16
HuH7	63.49	51.84	29.41	17.51	51.21	53.81	74.81
	±4.73	±5.86	±10.04	±9.18	±6.10	±4.34	±0.69
PC-3	76.24	70.00	89.70	89.04	82.49	82.68	68.93
	±3.95	±4.33	±5.14	±4.82	±4.94	±3.18	±5.42
A549	59.21	20.06	63.34	65.14	53.12	66.55	49.00
	±2.48	±3.66	±3.22	±5.91	±6.76	±3.61	±1.46
MG63	31.07	−11.31	76.84	78.42	32.84	27.76	69.87
	±4.71	±22.51	±2.09	±3.89	±7.29	±2.65	±1.61
HSF	66.66	21.54	65.30	62.05	59.29	66.72	26.79
	±5.79	±6.83	±7.33	±6.77	±8.73	±5.31	±6.62

Interestingly, compounds HB161 and HB162 could display the highest mean GI% (72.63% and 72.04%, respectively), exceeding the DOX average GI% (68.07%). Furthermore, among the compounds under investigation, compounds HB169, HB121, and HB163 had an exceptional mean GI% (62.27, 61.91, and 58.50%, respectively). Among the compounds provided, compound HB123 may exhibit the lowest mean GI% (41.59%), as shown in Table 1.

Furthermore, compound HB123 demonstrated favorable safety and selectivity toward malignant cells, as reflected by its low GI% in normal HSF.

#### 2.2.2. Half-maximal inhibitory concentrations (IC_50_) against the PC-3, MCF-7, and HCT-116 cancer cell lines assessment

**Fig. 2:**
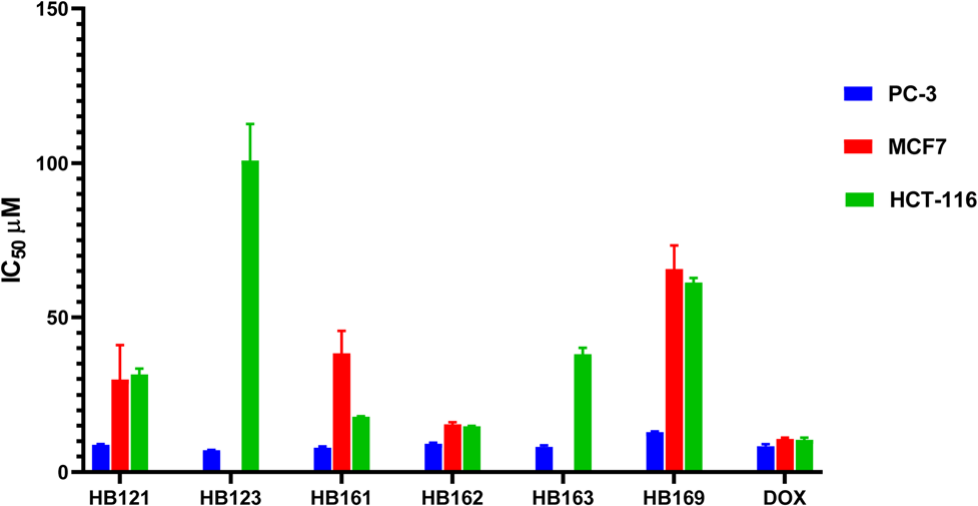
The half-maximal inhibitory concentrations (IC_50_) for the newly synthesized compounds (HB121–HB169) using PC-3, MCF7, and HCT-116 human cell lines for cancer. Quantitative analysis and graphical representation were performed using GraphPad Prism (version 8.2). Values are expressed as the mean ± standard deviation (SD) of at least three independent experiments. Statistical evaluation was performed using one-way analysis of variance (ANOVA) followed by Tukey's post hoc multiple-comparison test. *P < 0.05 was considered statistically significant compared with doxorubicin.

Following initial screening, cell lines with favorable GI% were used to calculate the IC_50_ values of the newly synthesized compounds (HB121–HB169). Thus, PC-3, MCF-7, and HCT-116 cancer cells were assessed using the SRB colorimetric assay methodology,^40^Fig. 2. The derivatives HB121–HB169 were evaluated against the chosen cancer cells at concentrations ranging from 0 to 50 µg mL^−1^.

##### 2.2.2.1 Structure–activity relationships (SAR) study

**Fig. 3:**
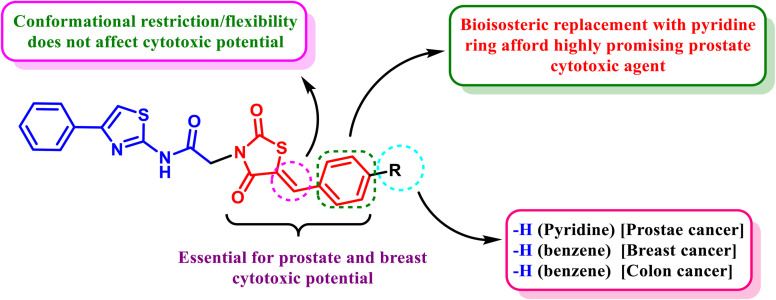
The novel cytotoxic candidates' link between structure and cytotoxic activity (HB121–HB169).

The bioisosteric replacement of the phenyl ring of the benzylidene thiazolidine-2,4-dione core with a pyridine ring and retaining the thiazole exocyclic olefinic bond (compound HB123), Fig. 3, resulted in the highest cytotoxic potency against prostate cancer with the lowest IC_50_ (7.14 µM), better than the reference cytotoxic drug DOX (8.41 µM). This enhanced activity can be attributed to the electron-deficient nature of the pyridine ring, which may improve hydrogen bonding and polar interactions within the biological target.

Besides, it was revealed that C-4 substitution of the phenyl ring of the benzylidene thiazolidine-2,4-dione core with bromide and giving up the thiazole exocyclic olefinic bond (compound HB161) can afford highly outstanding prostate cytotoxic potential (IC_50_ = 7.90 µM). The improved activity of this analogue may be associated with the strong electron-withdrawing effect and increased polarizability of the bromine atom, which can enhance hydrophobic and halogen-bonding interactions. Hence, we can argue that conformational restriction of our afforded compounds does not play a pivotal role in prostate cytotoxic biological activity, Fig. 3. The absence of benzylidene thiazolidine-2,4-dione core (compound HB169) can display the least prostate cytotoxic potential with an IC_50_ value of 12.93 µM, highlighting the critical role of this scaffold in maintaining cytotoxic potency.

Furthermore, the investigated compounds exhibited lower cytotoxic potential against breast cancer (MCF-7) cells compared to DOX. It was displayed that without C-4 substitution of the phenyl ring of the benzylidene thiazolidine-2,4-dione core and keeping the thiazole exocyclic olefinic bond (compound HB162), reasonable breast cytotoxic potential can be exhibited with IC_50_ = 15.44 µM (DOX IC_50_ = 10.74 µM). This suggests that the absence of substituents on the phenyl ring may reduce electronic modulation and binding affinity. Similarly, the absence of benzylidene thiazolidine-2,4-dione core (compound HB169) can display the lower breast cytotoxic potential with an IC_50_ value of 65.66 µM. Hence, we can argue that the benzylidene thiazolidine-2,4-dione core may be regarded as essential for prostate and breast cytotoxic potential.

In the case of colorectal cancer (HCT-116), it was demonstrated that, in contrast to DOX (IC_50_ = 10.46 µM), dependable colon cytotoxic potential could be achieved with IC_50_ values of 14.82 and 17.95 µM, respectively, without C-4 substitution of the phenyl ring of the benzylidene thiazolidine-2,4-dione core and maintaining the thiazole exocyclic olefinic bond (compound HB162) or C-4 substitution of the phenyl ring of the benzylidene thiazolidine-2,4-dione core with bromide and in the absence of the thiazole exocyclic olefinic bond (compound HB161), which may be attributed to a balance between steric bulk and electronic effects. With an IC_50_ value of 100.91 µM, compound HB123, which bioisosterically substitutes a pyridine ring for the phenyl ring of the benzylidene thiazolidine-2,4-dione core while keeping the thiazole exocyclic olefinic link, can exhibit the lowest colon cytotoxic potential, indicating that the pyridine substitution may not be favorable for interaction with targets relevant to colon cancer.

##### 2.2.2.2 Apoptosis-related indicators' protein expression

The apoptotic activity of the two analogues, HB161 and HB162, in PC-3 prostate cancer cells was assessed using a protein expression study. In order to shed light on how each molecule alters cell death signaling pathways, the evaluation concentrated on important pro- and anti-apoptotic indicators.

The pro-apoptotic proteins Caspase-3, Caspase-7, and Caspase-9 (1.87-, 1.12-, and 2.53-fold, respectively) and BAX (2.81-fold) were clearly upregulated after HB161 treatment. The anti-apoptotic and metastasis-associated proteins MMP-2, MMP-9, and BCL-2, on the other hand, were downregulated, exhibiting 1.31-, 1.89-, and 2.23-fold lower expression levels, respectively.

A similar pattern was observed with HB162, which also enhanced pro-apoptotic signaling. BAX was increased to 2.70-fold, while Caspase-3, Caspase-7, and Caspase-9 rose to 2.04-, 1.41-, and 1.93-fold, respectively. The anti-apoptotic markers MMP-2, MMP-9, and BCL-2, on the other hand, were suppressed, decreasing to 1.39-, 1.75-, and 2.48-fold, respectively.

**Fig. 4:**
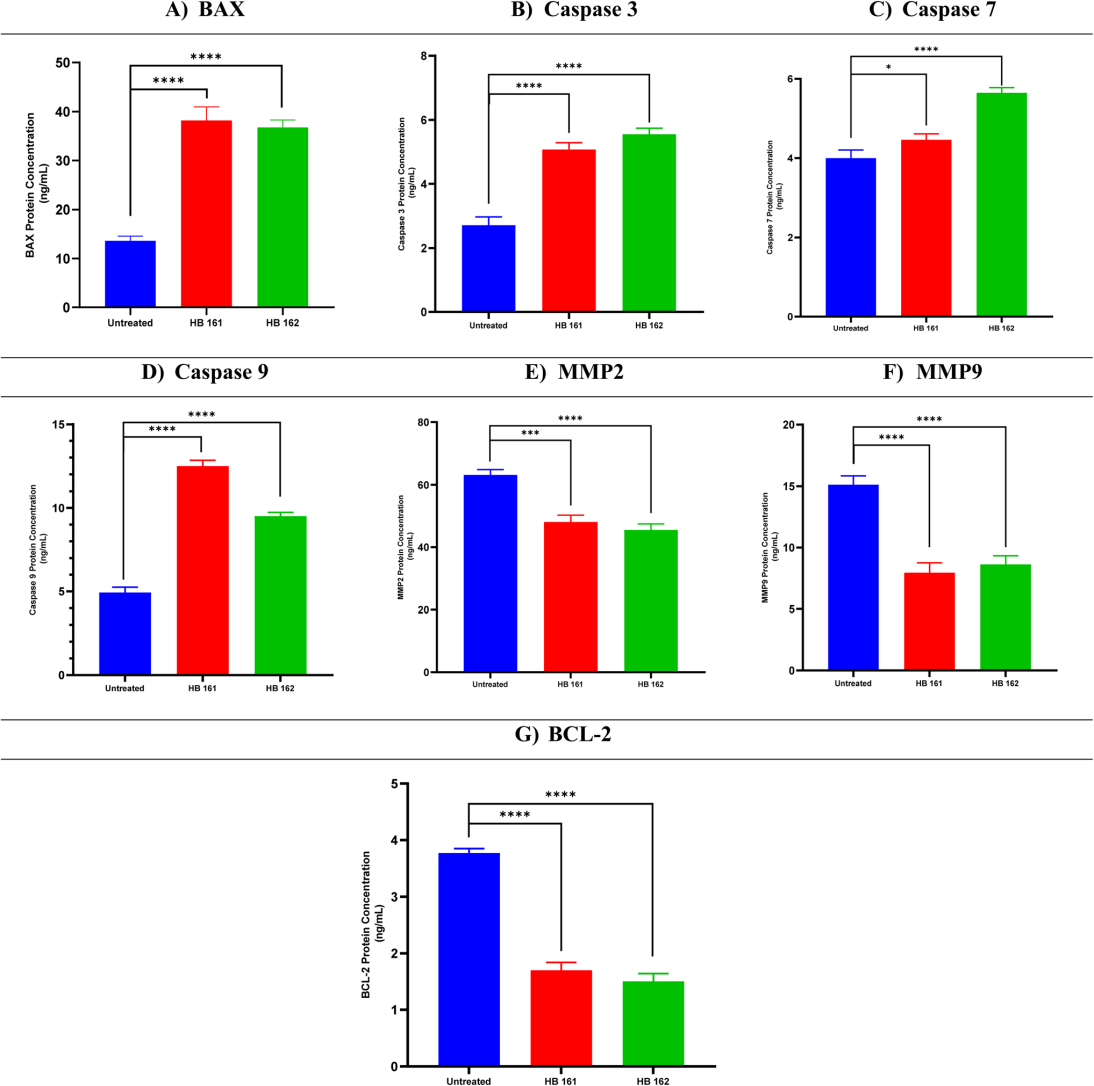
Protein expression profiles of MCF-7 cells after treatment with compounds HB161 and HB162 in comparison to untreated controls for BAX (A), caspase-3 (B), caspase-7 (C), caspase-9 (D), MMP-2 (E), MMP-9 (F), and BCL-2 (G). Quantitative analysis and graphical representation were performed using GraphPad Prism (version 8.2). Values are expressed as the mean ± standard deviation (SD) of at least three independent experiments. Statistical evaluation was performed using one-way analysis of variance (ANOVA) followed by Tukey's post hoc multiple-comparison test. *P < 0.05 was considered statistically significant compared with the untreated control.

When everything is taken into account, HB162 has a particularly potent apoptotic effect on PC-3 cells, as evidenced by the marked upregulation of pro-apoptotic mediators and the obvious suppression of proteins linked to invasion and apoptosis. The potential of both analogues as viable apoptosis-inducing candidates is further supported by the significant apoptotic activity displayed by HB161. Fig. 4 shows apoptotic markers.

#### 2.2.3. Evaluation of HB162 cell cycle arrest in the PC-3 cancer cell line

**Fig. 5:**
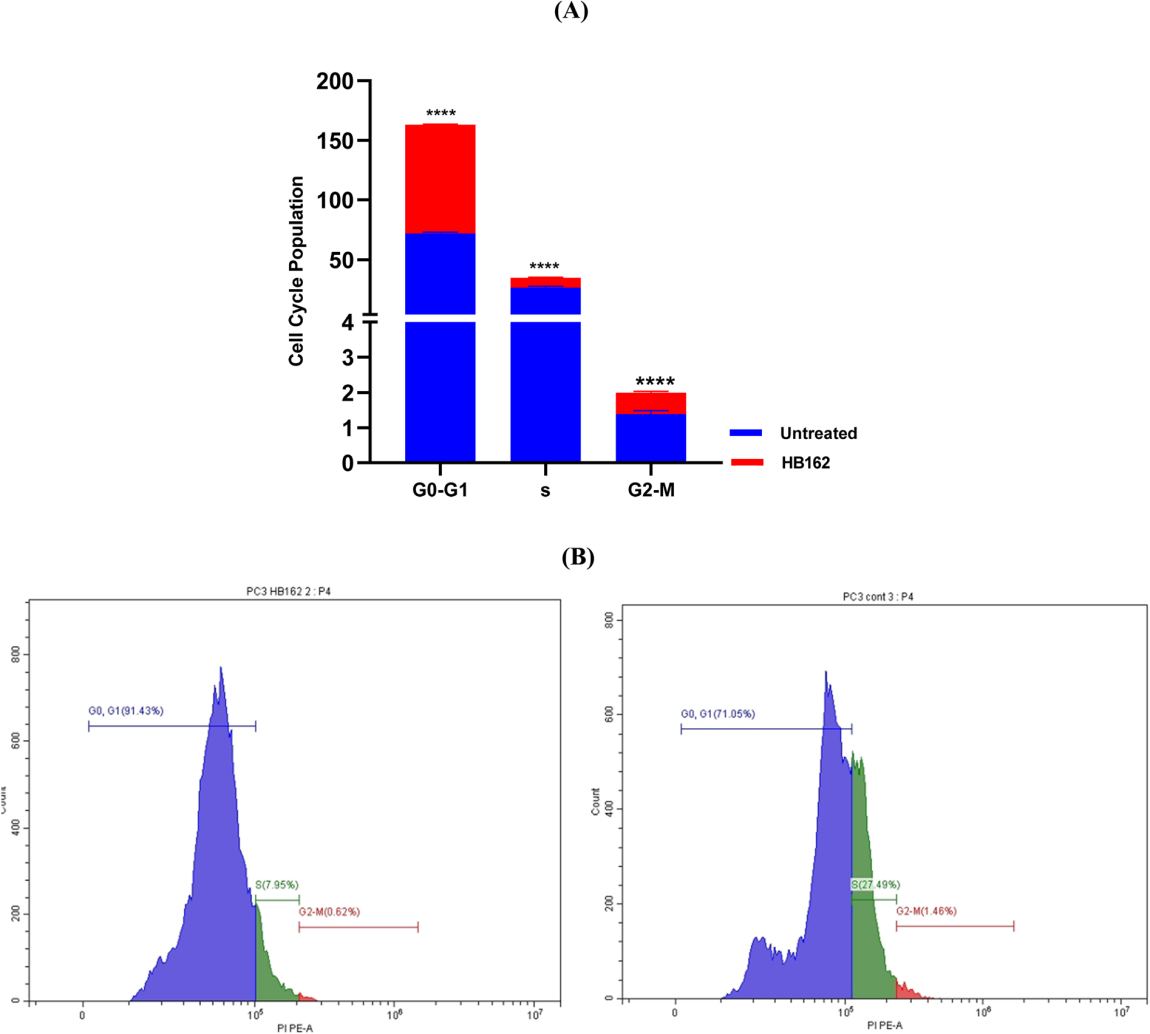
(A) Cell cycle studies using flow cytometry of PC-3 cancer cells treated with HB162 in comparison to untreated controls. (B) Representative histograms showing PC-3 cells exposed to HB162 (left) *versus* control cells (right).

A flow cytometric assay was conducted to ascertain which cell cycle phase is impacted by HB162, given its empowering anticancer activity against PC-3 cells. According to the analysis, after HB162 treatment, the proportion of cells in the G0–G1 phase rose significantly from 72% in untreated cells to 91.1%. Simultaneously, the G2–M phase population reduced somewhat from 1.39% to 0.62%, and the percentage of cells in the S phase dropped from 26.62% to 8.29% (Fig. 5). These findings suggest that by causing G0–G1 cell cycle arrest, HB162 efficiently suppresses cell growth.

To ensure the reliability of these findings, the experiment was repeated three times, and representative histograms are provided in the SI (Supplementary Figures S1 and S2).

### 2.3. Studies conducted *in silico*

#### 2.3.1. Molecular docking

To support HB161 and HB162 as BCL-2 downregulators, a molecular docking study was conducted focusing on the BCL-2 receptor, an essential component of the pathway that triggers apoptosis. *N*-heteroaryl sulfonamide, the BCL-2 receptor's large co-crystal inhibitor, was included as a reference for comparison after the BCL-2 receptor with ID 4IEH was chosen from the Protein Data Bank.

The binding scores for the analogues HB161 and HB162 were −7.31 and −7.34 kcal mol^−1^, respectively, with root mean square deviation (RMSD) values of 1.64 and 1.90 Å. Additionally, at an RMSD of 1.89 Å, the docked co-crystal of BCL-2 achieved a binding score of −9.94 kcal mol^−1^. The smaller sizes of the analogues HB161 and HB162 in comparison to the large co-crystal inhibitor (*N*-heteroaryl sulfonamide), which can establish more hydrophobic and hydrogen contacts, may be the reason for their lower scores.

**Fig. 6:**
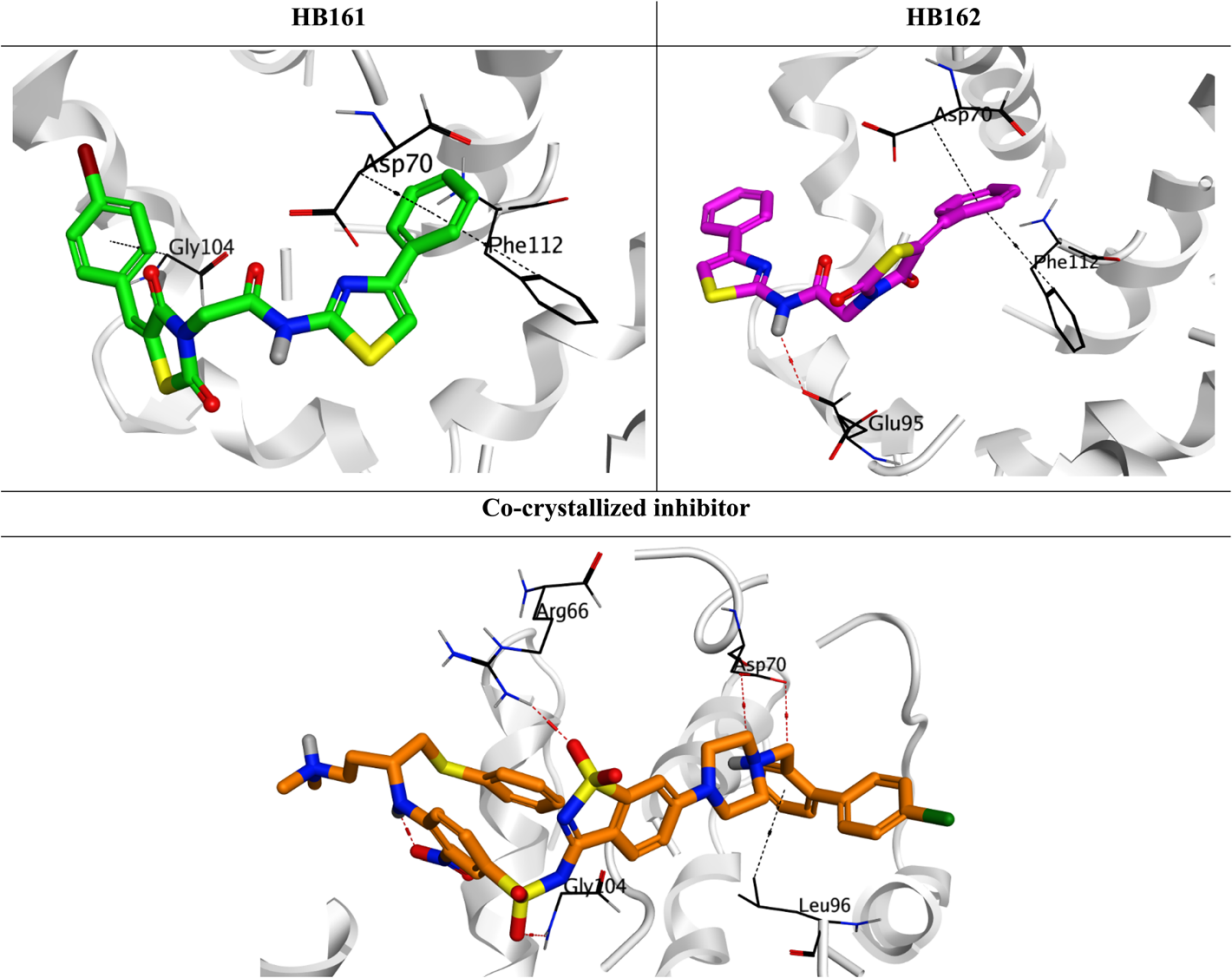
3D binding interactions between the co-crystal inhibitor and the analogues HB161 and HB162 in the BCL-2 receptor's active site (PDB ID: 4IEH).

Three pi-hydrogen interactions with Asp70, Phe112, and Gly104 fixed the small analogue HB161 inside the BCL-2's active pocket. However, in addition to forming a hydrogen bond with Glu95, the small homologue HB162 demonstrated the creation of two pi-hydrogen interactions with Asp70 and Phe112. Additionally, four hydrogen bonds were established between the docked BCL-2 co-crystal inhibitor and Arg66, Gly104, and Asp70 (2). Additionally, it created a single pi–hydrogen bond with Leu96 (Fig. 6).

As a result, it is strongly advised that the small analogues (HB161 and HB162) be considered as prospective BCL-2 downregulators. They may also be further optimized by using an extension strategy to reach the additional empty pocket of the BCL-2 active site.

#### 2.3.2. ADMET and physicochemical studies

**Fig. 7:**
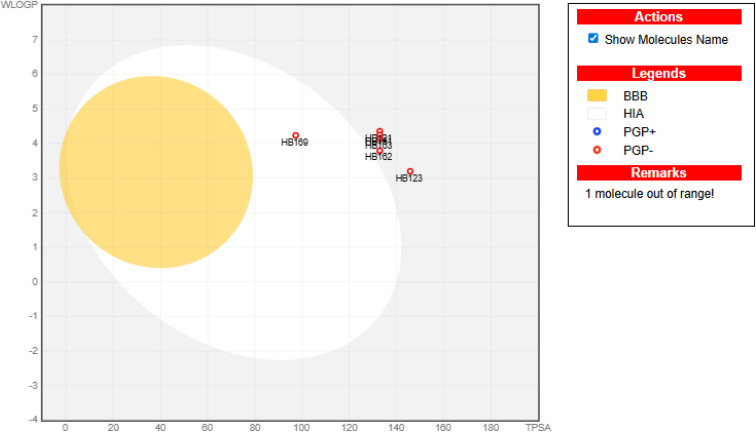
The Boiled-egg diagram for the newly synthesized analogues (HB121–HB169), along with DOX as a reference control.

It is worth noting that compound HB169 exhibited high gastrointestinal absorption, suggesting the potential for reliable oral bioavailability.^41,42^ However, all other afforded compounds (HB121, HB123, HB161, HB162, and HB163) can manifest low GIT absorption. So, they cannot be administered *via* the oral route, and further future proactive steps should be attempted to optimize and enhance their oral bioavailability *via* further structural modification and solubility improvement tools. Additionally, the synthesized compounds do not cross the blood–brain barrier, indicating a lower risk of adverse effects associated with the central nervous system. Notably, none of the synthesized compounds can act as substrates for P-glycoprotein (Pgp), indicating the potential for improved gastrointestinal bioavailability, as shown in Fig. 7.

Interestingly, none of the synthesized hybrids were found to violate Lipinski's rule of five.^43^ SI Fig. S3 presents bioavailability radar plots for the investigated compounds, providing insights into their bioavailability characteristics.

Furthermore, none of the synthesized compounds exhibited Ames toxicity, suggesting a reduced likelihood of mutagenicity.^44^ Additionally, the synthesized compounds are predicted to be safe with respect to cardiotoxicity, as they do not inhibit *h*ERG I channels involved in cardiac electrical activity.^45^ However, inhibition of *h*ERG II by all synthesized compounds may pose a potential risk of cardiac arrhythmia.^46^ All examined derivatives appear to possess hepatotoxic potential, as shown in Table 2.

**Table 2:** The predicted ADME profiles and physicochemical characteristics of the analogues (HB121–HB169) in comparison with DOX^a^

		HB121	HB123	HB161	HB162	HB163	HB169	DOX
Molecular properties	Molar refractivity	118.93	116.77	126.35	118.97	123.66	93.47	132.66
	TPSA (A^z^)	132.91	145.80	132.91	132.91	132.91	97.28	206.07
	Log *P* o/w (WLOGP)	4.36	3.20	4.25	3.80	4.14	4.24	−0.32
	Consensus log *P* o/w	3.68	2.77	3.97	3.43	3.89	3.77	0.44
	Water solubility	MS	MS	MS	MS	MS	MS	S
Pharmacokinetics parameters and drug/lead likeness	GI absorption	Low	Low	Low	Low	Low	High	Low
	BBB permeant	No	No	No	No	No	No	No
	P-gp substrate	No	No	No	No	No	No	Yes
	CYP1A2 inhibitor	Yes	No	Yes	Yes	Yes	Yes	No
	CYP2C19 inhibitor	Yes	Yes	Yes	Yes	Yes	Yes	No
	CYP2C9 inhibitor	Yes	Yes	Yes	Yes	Yes	Yes	No
	CYP2D6 inhibitor	No	No	No	No	No	Yes	No
	CYP3A4 inhibitor	Yes	Yes	Yes	Yes	Yes	Yes	No
	Drug likeness (lipinski)	Yes	Yes	Yes	Yes	Yes	Yes	No
	Lead likeness	No	No	No	No	No	No	No
Toxicity parameters	Ames toxicity	No	No	No	No	No	No	Yes
	Max. Tolerated dose (log mg per kg per day)	0.63	0.363	0.638	0.062	0.033	0.008	0.405
	*h*ERG I inhibitor	No	No	No	No	No	No	No
	*h*ERG II inhibitor	Yes	Yes	Yes	Yes	Yes	Yes	No
	Oral rat acute toxicity (LD_50_) (mol kg^−1^)	2.892	2.996	2.932	2.723	2.767	2.604	3.934
	Oral rat chronic toxicity (log mg per kg_bw per day)	1.69	1.166	1.609	0.886	0.954	1.113	3.435
	Hepatotoxicity	Yes	Yes	Yes	Yes	Yes	Yes	No
	Minnow toxicity (log mM)	0.298	1.443	−0.144	1.129	0.071	1.889	8.145

a MS: moderately soluble; S: soluble.

## 3. Conclusion

In this study, a rational design strategy integrating fragment merging, bioisosteric replacement, and conformational control successfully yielded a new class of benzylidene thiazolidine-2,4-dione/*N*-(phenylthiazole) acetamide hybrids with promising anticancer potential. With cytotoxic profiles that were either better than or comparable to DOX across a variety of cancer cell lines, HB161, HB162, and HB123 were found to be the most active analogues *via* systematic structural diversification, which also led to distinct SAR trends. Notably, HB162 showed broad-spectrum antiproliferative effects and caused a noticeable G0/G1 cell-cycle arrest, while HB123 and HB161 showed strong activity against PC-3 prostate cancer cells. By upregulating BAX and Caspases-3/7/9 and suppressing the anti-apoptotic mediators BCL-2, MMP-2, and MMP-9, mechanistic validation showed that HB161 and HB162 activate apoptotic pathways to produce their anticancer effects. The small analogues (HB161 and HB162) were suggested by molecular docking to be promising BCL-2 downregulators. They may be further optimized by using an extension strategy to reach the additional empty pocket of the BCL-2 active site. Although future optimization is necessary to improve oral bioavailability and reduce potential cardiotoxic and hepatotoxic liabilities, complementary ADMET predictions indicated favorable drug-likeness, lack of mutagenicity, and acceptable pharmacokinetic behavior. Taken together, these results present a flexible hybrid scaffold with proven biological activity and a well-defined mechanistic basis, establishing these compounds—specifically HB161 and HB162—as viable lead candidates for additional preclinical research in solid tumors, including prostate cancer. These hybrids may progress toward clinically useful anticancer treatments with further structural improvement and thorough pharmacological analysis.

## 4. Materials and methods

### 4.1. Chemistry

Compounds 3,^47^4,^48^ and thiazolidine-2,4-dione^49^ and its potassium salt were synthesized using the reported literature approach (see also SI for the preparation methods).^39^

#### 4.1.1 Synthesis of 1-phenyl-3-(4-phenylthiazol-2-yl)thiourea (HB169)

4-Phenylthiazol-2-amine (**3**) (1 mmol) and phenyl isothiocyanate (1.5 mmol) reacted in 15 mL of EtOH to form compound HB169. After four hours of refluxing, the mixture was left to cool. White crystals were obtained by filtering the produced crystals, washing them with EtOH, and then drying them; yield = 81%; MP = 232–233 °C; FT-IR (*ν*, cm^−1^): 3144, 2936, 1593, 1570, 1550, 1513, 1493, 1170, 1153, 681, 460, 497; ^1^H NMR (600 MHz, pyridine-*d*_5_) *δ* 13.34 (s, 1H, NH), 12.76 (s, 1H, NH), 8.68 (s, 1H, Ar–H), 8.04 (d, *J* = 7.7 Hz, 1H, Ar–H), 7.97–7.94 (m, 1H, Ar–H), 7.53 (d, *J* = 10.2 Hz, 1H, Ar–H), 7.42–7.34 (m, 4H, Ar–H), 7.32–7.27 (m, 1H, Ar–H), 7.15 (t, *J* = 3.6 Hz, 2H, Ar–H); ^13^C NMR (151 MHz, pyridine-*d*_5_) *δ* 178.26, 163.15, 139.63, 133.90, 129.00, 128.94, 128.37, 126.22, 125.54, 124.01, 105.89; MS (ESI): *m*/*z* = calcd. For C_16_H_13_N_3_S_2_ [M^+^]: 311.4, found 309.8 [M^+^ − 2], 310.8 [M^+^ − 1], 311.8 [M^+^].

#### 4.1.2 Synthesis of 2-(2,4-dioxothiazolidin-3-yl)-*N*-(4-phenylthiazol-2-yl)acetamide (HB045)

2-Chloro-*N*-(4-phenylthiazol-2-yl)acetamide (4) (20 mmol) and the potassium salt of 2,4-thiazolidinedione (30 mol) in DMF (10 mL) were reacted to synthesize compound HB045. For four hours, the mixture was heated to 80 °C. Following completion, 30 mL of water was added, and the precipitate that resulted was separated, cleaned with water, and dried to produce yellow powder; yield = 90%; MP = 219–220 °C; FT-IR (*ν*, cm^−1^): 3224, 3090, 1759, 1654, 1559, 1325, 1298, 1275, 1182, 776; ^1^H NMR (500 MHz, DMSO-*d*_6_) *δ* 12.68 (s, 1H, NH), 7.88 (d, *J* = 7.6 Hz, 2H, Ar–H), 7.65 (s, 1H, Ar–H), 7.42 (t, *J* = 7.5 Hz, 2H, Ar–H), 7.32 (t, *J* = 7.1 Hz, 1H, Ar–H), 4.47 (s, 2H, CH_2_), 4.32 (s, 2H, CH_2_); ^13^C NMR (151 MHz, pyridine-*d*_6_) *δ* 172.01, 171.51, 164.79, 158.38, 128.83, 127.92, 126.26, 108.22, 43.79, 34.50; HRMS calcd. For C_14_H_11_N_3_O_3_S_2_ [M^+^]: 333.3800, found 334.0315 [M^+^ + 1].

#### 4.1.3 General preparation of thiazolidinone-based arylidene derivatives

Piperidine (50 µL) and AcOH (50 µL) were added to a mixture of HB045 (15 mmol) and the suitable aldehyde (18 mmol) in EtOH (20 mL), and the reaction was refluxed for 24 hours. Following the addition of 30 mL of H_2_O, the resulting solid was filtered, cleaned with aqueous EtOH, and, if necessary, recrystallized from EtOH : DMF (1 : 1).

##### 4.1.3.1 Synthesis of 2-(5-benzylidene-2,4-dioxothiazolidin-3-yl)-*N*-(4-phenylthiazol-2-yl)acetamide (HB162)

After being synthesized from HB045 (15 mmol) and benzaldehyde (18 mol), compound HB162 was separated as a white powder; yield = 42%; MP = 235–236 °C; FT-IR (*ν*, cm^−1^): 3286, 1741, 1692, 1670, 1610, 1560, 1483, 1376, 1148, 725, 665, 515; ^1^H NMR (500 MHz, DMSO-d_6_) *δ* 12.77 (s, 1H, NH), 8.00 (s, 1H, Ar–H), 7.89 (d, *J* = 7.5 Hz, 2H, Ar–H), 7.67 (s, 2H, Ar–H), 7.57 (t, *J* = 7.3 Hz, 2H, Ar–H), 7.53 (d, *J* = 7.1 Hz, 1H, Ar–H), 7.43 (t, *J* = 7.6 Hz, 2H, Ar–H), 7.33 (t, *J* = 7.3 Hz, 1H, Ar–H), 4.66 (s, 2H, CH_2_); ^13^C NMR (151 MHz, pyridine-*d*_5_) *δ* 167.54, 165.70, 164.76, 158.40, 134.27, 133.33, 130.70, 130.32, 129.32, 128.85, 127.94, 126.28, 122.98, 121.60, 108.29, 43.96; MS (ESI): *m*/*z* = calcd. For C_21_H_15_N_3_O_3_S_2_ [M^+^]: 421.4, found 419.6 [M^+^-2], 421.7 [M^+^-1]; HRMS calcd. For C_21_H_15_N_3_O_3_S_2_ [M^+^]: 421.4890, found 422.0625 [M^+^].

##### 4.1.3.2 Synthesis of 2-(5-(4-bromobenzylidene)-2,4-dioxothiazolidin-3-yl)-*N*-(4-phenylthiazol-2-yl)acetamide (HB161)

HB045 (15 mmol) and 4-bromo-benzaldehyde (18 mol) were combined to synthesize compound HB161, which was then separated as a white powder; yield = 47%; MP = 263–264 °C; FT-IR (*ν*, cm^−1^): 3363, 3118, 2980, 1756, 1686, 1618, 1838, 1483, 1275, 1071, 1005, 814; ^1^H NMR (500 MHz, DMSO-*d*_6_) *δ* 12.77 (s, 1H, NH), 7.98 (s, 1H, Ar–H), 7.89 (d, *J* = 7.6 Hz, 2H, Ar–H), 7.77 (d, *J* = 8.4 Hz, 2H, Ar–H), 7.67 (s, 1H, Ar–H), 7.61 (d, *J* = 8.4 Hz, 2H, Ar–H), 7.43 (t, *J* = 7.6 Hz, 2H, Ar–H), 7.32 (t, *J* = 7.3 Hz, 1H, Ar–H), 4.65 (s, 2H, CH_2_); ^13^C NMR (151 MHz, pyridine-*d*_5_) *δ* 174.74, 171.78, 166.94, 158.09, 137.84, 134.37, 133.99, 133.42, 133.20, 131.33, 130.29, 129.40, 127.78, 124.95, 109.73, 45.52; HRMS calcd. For C_21_H_14_BrN_3_O_3_S_2_ [M^+^]: 500.3850, found 501.9712 [M^+^ + 1], 502.9741 [M^+^ + 2].

##### 4.1.3.3 Synthesis of 2-(5-(4-chlorobenzylidene)-2,4-dioxothiazolidin-3-yl)-*N*-(4-phenylthiazol-2-yl)acetamide (HB163)

HB045 (15 mmol) and 4-chloro-benzaldehyde (18 mol) were combined to synthesize compound HB163, which was then separated as a white powder; yield = 73%; MP = 258–259 °C; FT-IR (*ν*, cm^−1^): 3364, 3117, 2977, 1758, 1686, 1677, 1654, 1611, 1542, 1483, 1542, 1370, 1324, 1275, 1073, 1061, 828; ^1^H NMR (500 MHz, DMSO-d_6_) *δ* 12.77 (s, 1H, NH), 8.00 (s, 1H, Ar–H), 7.87 (t, *J* = 14.6 Hz, 2H, Ar–H), 7.69 (d, *J* = 4.4 Hz, 1H, Ar–H), 7.68 (d, *J* = 5.2 Hz, 2H, Ar–H), 7.63 (d, *J* = 8.5 Hz, 2H, Ar–H), 7.43 (t, *J* = 7.6 Hz, 2H, Ar–H), 7.32 (t, *J* = 7.3 Hz, 1H, Ar–H), 4.65 (s, 2H, CH_2_); ^13^C NMR (151 MHz, pyridine-*d*_5_) *δ* 168.74, 167.07, 166.21, 159.89, 137.80, 134.34, 133.46, 133.14, 131.01, 130.35, 129.45, 127.79, 123.77, 109.80, 45.51; HRMS calcd. For C_21_H_14_ClN_3_O_3_S_2_ [M^+^]: 455.93100, found 456.0234 [M^+^ + 1], 457.0262 [M^+^ + 2], 458.0226 [M^+^ + 3], 459.0134 [M^+^ + 4], 460.0134 [M^+^ + 5].

##### 4.1.3.4 Synthesis of 2-(5-(4-fluorobenzylidene)-2,4-dioxothiazolidin-3-yl)-*N*-(4-phenylthiazol-2-yl)acetamide (HB121)

HB045 (15 mmol) and 4-flouro-benzaldehyde (18 mol) were combined to synthesize compound HB121, which was then separated as a green powder; yield = 53%; MP = 227–228 °C; FT-IR (*ν*, cm^−1^): 3363, 3252, 3208, 2948, 2922, 1687, 1619, 1546, 1517, 1479, 1436, 1197, 1013, 992; ^1^H NMR (500 MHz, DMSO-*d*_6_) *δ* 12.77 (s, 1H, NH), 8.01 (s, 1H, Ar–H), 7.89 (d, *J* = 7.5 Hz, 2H, Ar–H), 7.74 (dd, *J* = 8.1, 5.6 Hz, 2H, Ar–H), 7.67 (s, 1H, Ar–H), 7.45–7.37 (m, 4H, Ar–H), 7.32 (t, *J* = 7.3 Hz, 1H, Ar–H), 4.65 (s, 2H, CH_2_); ^13^C NMR (151 MHz, DMSO-*d*_6_) *δ* 166.76, 160.27, 154.28, 149.02, 134.87, 129.11, 128.03, 126.01, 107.01, 45.30; HRMS calcd. For C_21_H_14_FN_3_O_3_S_2_ [M^+^]: 439.4794, found 440.0522 [M^+^ + 1], 441.0554 [M^+^ + 2], 442.0463 [M^+^ + 3], 443.0502 [M^+^ + 4].

##### 4.1.3.5 Synthesis of 2-(2,4-dioxo-5-(pyridin-2-ylmethylene)thiazolidin-3-yl)-*N*-(4-phenylthiazol-2-yl)acetamide (HB123)

HB045 (15 mmol) and pyridine-2-carbaldehyde (18 mol) were combined to create compound HB123, which was then separated as a grey powder; yield = 49%; MP = 274–275 °C; FT-IR (*ν*, cm^−1^): 3254, 3203, 3050, 3003, 2936, 1738, 1682, 1656, 1616, 1582, 1408, 1386, 1276, 1249, 1148, 779; ^1^H NMR (500 MHz, DMSO-*d*_6_) *δ* 12.76 (s, 1H, NH), 8.79 (d, *J* = 4.2 Hz, 1H, Ar–H), 8.02 (s, 1H, Ar–H), 7.97 (t, *J* = 7.6 Hz, 1H, Ar–H), 7.93–7.86 (m, 3H, Ar–H), 7.67 (s, 1H, Ar–H), 7.50–7.38 (m, 3H, Ar–H), 7.32 (t, *J* = 7.3 Hz, 1H, Ar–H), 4.64 (s, 2H, CH_2_); ^13^C NMR (151 MHz, pyridine-*d*_5_) *δ* 171.63, 166.09, 164.94, 151.63, 137.12, 129.77, 128.84, 128.80, 127.92, 127.70, 127.52, 126.28, 123.81, 108.25, 43.36; MS (ESI): *m*/*z* = calcd. For C_20_H_14_N_4_O_3_S_2_ [M^+^]: 422.4, found 420.7 [M^+^ − 2], 421.7 [M^+^ − 1], 422.7 [M^+^]; HRMS calcd. For C_20_H_14_N_4_O_3_S_2_ [M^+^]: 422.4770, found 440.0522 [M^+^ + 1], 441.0554 [M^+^ + 2], 442.0463 [M^+^ + 3], 443.0502 [M^+^ + 4].

### 4.2. Evaluation of biology

#### 4.2.1. Assay for growth inhibition percentage

All cancer cell lines used in this study (Hepatocellular carcinoma (HuH-7), lung malignancies (A549, H460), breast cancer (MCF-7), osteosarcoma (MG63), colorectal carcinoma (HCT-116), and prostate cancer (PC-3)) were obtained from Vacsera (Giza, Egypt), while the normal human skin fibroblast (HSF) cell line was obtained from the American Type Culture Collection (ATCC, USA). Cells were cultured in Dulbecco's Modified Eagle Medium (DMEM) supplemented with 10% fetal bovine serum (FBS), 1% penicillin–streptomycin, and maintained at 37 °C in a humidified incubator with 5% CO_2_. The cytotoxic activity of compounds HB121–HB169 was evaluated using the sulforhodamine B (SRB) colorimetric assay, following previously reported protocols.^40,50^ Briefly, cells were seeded in 96-well plates at an appropriate density (5 × 10^3^–1 × 10^4^ cells/well) and allowed to attach for 24 h prior to treatment. Cells were then treated with the tested compounds at a fixed concentration for 48 h. After treatment, cells were fixed with trichloroacetic acid (TCA), stained with SRB dye, and the absorbance was measured at 540 nm using a microplate reader. Growth inhibition percentages (GI%) were calculated relative to untreated control cells.

#### 4.2.2. Assay for cytotoxic inhibitory concentration 50 (IC_50_)

Half-maximal inhibitory concentration (IC_50_) values were determined for selected compounds (HB121–HB169) against PC-3, MCF-7, and HCT-116 cancer cell lines using the SRB assay.^51,52^ Cells were treated with a series of compound concentrations (0–50 µg mL^−1^) for 48 h under the same culture conditions described above. Dose–response curves were generated, and IC_50_ values were calculated using nonlinear regression analysis. All experiments were conducted in triplicate (*n* = 3 independent biological replicates). Statistical analysis was performed using GraphPad Prism software. Data were expressed as mean ± SD, and comparisons between groups were performed using one-way ANOVA followed by appropriate post hoc tests. A *p*-value < 0.05 was considered statistically significant.^51–53^

#### 4.2.3. Expression of proteins linked to apoptosis

The expression of apoptosis-associated proteins for HB161 and HB162 in PC-3 prostate cancer cells was examined in order better to clarify the apoptotic activity of the most promising derivatives. The purpose of this analysis was to determine whether their cytotoxic effects were caused by altering important regulators of programmed cell death. Cells were treated with the tested compounds under defined conditions, and protein expression levels were analyzed using appropriate biochemical assays as described in (SI).^54,55^ A better understanding of the pro-apoptotic mechanisms triggered by these substances was enabled by identifying notable changes in apoptotic markers through comparative profiling of treated and untreated PC-3 cells. Comparative analysis between treated and untreated cells was performed to identify significant changes in apoptotic markers, providing insight into the underlying mechanism of cytotoxic activity.

#### 4.2.4. DNA content analysis using flow cytometry (compound HB162)

The effect of HB162 on cell cycle regulation was examined by plating PC-3 prostate cancer cells (2 × 10^5^ cells per well) in 12-well culture plates and exposing them to varying concentrations of the compound for predetermined incubation times, as previously reported.^56–58^ Following treatment, the cells were extracted and preserved in ice-cold 70% ethanol made in phosphate-buffered saline (PBS) for an entire night at −20 °C. After fixation, the cells were rinsed and then resuspended in PBS that contained 0.1% Triton X-100, 40 µg mL^−1^ propidium iodide, and 0.1 mg mL^−1^ RNase A (Sigma, USA). A Becton Dickinson flow cytometer fitted with a 488 nm argon laser was then used to analyze the samples. The percentage of cells in each stage of the cell cycle was measured using the acquired fluorescence profiles, which shed light on changes in cell cycle progression brought on by HB162.

### 4.3. Studies conducted *in silico*

#### 4.3.1. Molecular docking

The AutoDock Vina^59^ and PyMOL^60^ were employed to dock the interesting analogues (HB161 and HB162) to the BCL-2 receptor following its synthesis by energy minimization and partial charge optimization.^61,62^ With a grid spacing of 1000 Å, the grid size was set to 30 × 30 × 30 *xyz* points, and the grid center was set to (*x*, *y*, *z*): 9.92, 20.528, and 19.532. 3D hydrogenation, correction, and energy reduction were used to create the PDB of the chosen protein receptor, 4IEH ID.^63^ The BCL-2 co-crystal inhibitor was contrasted with its analogues (HB161 and HB162) in terms of binding mode and score. Additionally, a redocking process for the BCL-2 co-crystal inhibitor validated the software's validity (RMSD < 2 Å).^64,65^

#### 4.3.2. Physicochemical investigations and *in silico* ADMET

The novel analogues' pharmacokinetic behavior was predicted using SwissADME, a free web tool developed by the Swiss Institute of Bioinformatics (SIB) (https://www.swissadme.ch/index.php). The site helped assess each chemical structure's potential as a therapeutic candidate by generating predictions about its ADME features based on the SMILES codes entered.^66^ Additionally, based on their chemical structures, the pkCSM web platform (https://biosig.lab.uq.edu.au/pkcsm/prediction_single/toxicity_1765271299.12) was used to estimate the toxicity of the novel compounds *in silico*.^67^ The novel analogues' physicochemical and ADMET properties were evaluated computationally against DOX.

## Author contributions

Conceptualization and supervision: Saad Shaaban and Ahmed A. Al-Karmalawy; data curation, visualization, methodology, and writing–review & editing: Saad Shaaban, Samia S. Hawas, Marwa Sharaky, Hussein Ba-Ghazal, Ayman Abo Elmaaty, Khadra B. Alomari, Mohamed Alaasar, Asma M. Elsharif, Fatema S. Alatawi, Mohamed Alaa Mohamed, Arwa Omar Al Khatib, Ahmed A. Al-Karmalawy. Finally, all authors revised and approved the final submitted version of the manuscript.

## Conflicts of interest

The authors declared no conflict of interest.

## Funding

This work was supported by the Deanship of Scientific Research, Vice Presidency for Graduate Studies and Scientific Research, King Faisal University, Saudi Arabia [Grant No. KFU261378].

## Supplementary Material

RA-016-D6RA01323F-s001

## Data Availability

The data supporting this article have been included in the manuscript and the Supporting Information (SI). Supplementary information: chemistry (methods and materials); spectroscopic data; biological data; ADMET and physicochemical studies. See DOI: https://doi.org/10.1039/d6ra01323f.
