# Supplementary material for: Hybrid benzylidene thiazolidine-2,4-diones as potent apoptosis-inducing anticancer agents: design-driven optimization, cytotoxic profiling, and mechanistic validation in prostate cancer
Source: RSC Adv. 2026 Apr 12;16(21):19144–57. doi: 10.1039/d6ra01323f (PMC13070299; doi:10.1039/d6ra01323f)
Supplement: RA-016-D6RA01323F-s001 [file RA-016-D6RA01323F-s001.pdf]

Supplementary Information (SI) for RSC Advances.
This journal is © The Royal Society of Chemistry 2026

Supporting Information

Hybrid Benzylidene Thiazolidine-2,4-diones as Potent Apoptosis-Inducing Anticancer Agents: Design-Driven Optimization, Cytotoxic Profiling, and Mechanistic Validation in Prostate Cancer

Saad Shaaban[1,¥], Samia S. Hawas[2,¥], Marwa Sharaky[3], Hussein Ba-Ghazal[1], Ayman Abo Elmaaty[4,5], Khadra B. Alomari[6], Mohamed Alaasar[7], Asma M. Elsharif[8], Fatema S. Alatawi[9], Mohamed Alaa Mohamed[10], Arwa Omar Al Khatib[11], Ahmed A. Al-Karmalawy[2,12*]

[1] Department of Chemistry, College of Science, King Faisal University, Al-Ahsa 31982, Saudi Arabia.
[2] Department of Pharmaceutical Chemistry, Faculty of Pharmacy, Horus University-Egypt, New Damietta 34518, Egypt.
[3] Cancer Biology Department, National Cancer Institute (NCI), Cairo University, Cairo, Egypt.

[4] Medicinal Chemistry Department, Faculty of Pharmacy, Port Said University, Port Said 42526, Egypt.
[5] Medicinal Chemistry Department, Clinical Pharmacy Program, East Port Said National University, Port Said 42526, Egypt.
[6] Jazan University, Department of Physical Sciences, Chemistry Division, P.O. Box 114, 45142, Jazan, Kingdom of Saudi Arabia.
[7] Department of Chemistry, Faculty of Science, Cairo University, Giza, Egypt.
[8] Department of Chemistry, College of Science, Imam Abdulrahman Bin Faisal University, Dammam 31441, Saudi Arabia.
[9] Department of Biochemistry, Faculty of Science, University of Tabuk, Tabuk, Saudi Arabia.
[10] Department of Chemistry, Faculty of Science, Mansoura University, Mansoura, Egypt
[11] Faculty of Pharmacy, Al-Ahliyya Amman University, Amman, Jordan.

[12] Department of Pharmaceutical Chemistry, College of Pharmacy, The University of Mashreq, Baghdad 10023, Iraq.

*Corresponding authors:

Ahmed A. Al-Karmalawy; Email: akarmalawy@horus.edu.eg

Saad Shaaban; Email: sibrahim@kfu.edu.sa

¥: These authors contributed equally.

Chemistry: Methods and Materials

All solvents and reagents were purchased from Sigma Aldrich and were used without any prior purifications. Melting points (MP) in ºC were recorded on the Gallenkamp instrument. The IR spectra (KBr, ύ cm^{-1}) were recorded at King Faisal University on a Mattson 5000 FTIR Spectrophotometer.

The structure characterization of the prepared materials is based on ^{1}H-NMR, ^{13}C-NMR (Agilent Technologies 500 and 600 MHz VNMRS in DMSO-d_6 and pyridine-d_5 solution, with tetramethylsilane as internal standard. The high-resolution mass spectra were measured at Martin Luther University, Halle, Germany, on Thermo Scientific Q Exactive Plus mit HESI, APCI, sowie LIFDI-Quelle (Linden CMS).

Synthesis of 4-phenylthiazol-2-amine (3)[1]

A mixture of phenacyl bromide (3 mmol) and thiourea (3.6 mmol) in EtOH (15 mL) was refluxed for 4 h. After cooling, the resulting precipitate was filtered, washed with water, and dried to give the compound 3 as a white solid in 99 % yield, mp 150-152 ºC (Lit. 149-150 ºC).

Synthesis of 2-chloro-N-(4-phenylthiazol-2-yl)acetamide (4)[2]

To a solution of 4-phenylthiazol-2-amine (**3**) (1 mmol) in dichloromethane (15 ml), triethylamine (50 µl) was added while stirring at room temperature. The mixture was then cooled to 0-5 ºC and then chloroacetyl chloride (1.5 mmol) was added dropwise, and the mixture was stirred for 1 h, and then for 3 h at room temperature. After completion, water (30 mL) was added, and the reaction was extracted 3 times with saturated brine. The organic layers were combined, dried over anhydrous sodium sulfate, evaporated to dryness to give a white solid, yield: 89%, m.p.141-142 ºC (Lit. 143-144 ºC).

Synthesis of Thiazolidine-2,4-dione [3]

A mixture of thiourea (100 mmol) and monochloroacetic acid (100 mmol) was dissolved in distilled water (250 mL) and then refluxed for 3 days. The mixture was then cooled, and the resulting white crystals were isolated and dried.

Synthesis of Potassium salt of 2,4-thiazolidinediones [3]

Thiazolidine-2,4-dione (8 g) was dissolved in 12.5 ml of ethanol. To this solution, KOH (4.2 g) in ethanol (10 mL) was added. The mixture was stirred for 2 h. The white crystalline solid

was collected after filtration, washed with ethanol, and dried under vacuum to get the potassium salt of thiazolidinedione.

Spectroscopic Data

1-phenyl-3-(4-phenylthiazol-2-yl)thiourea (HB169)

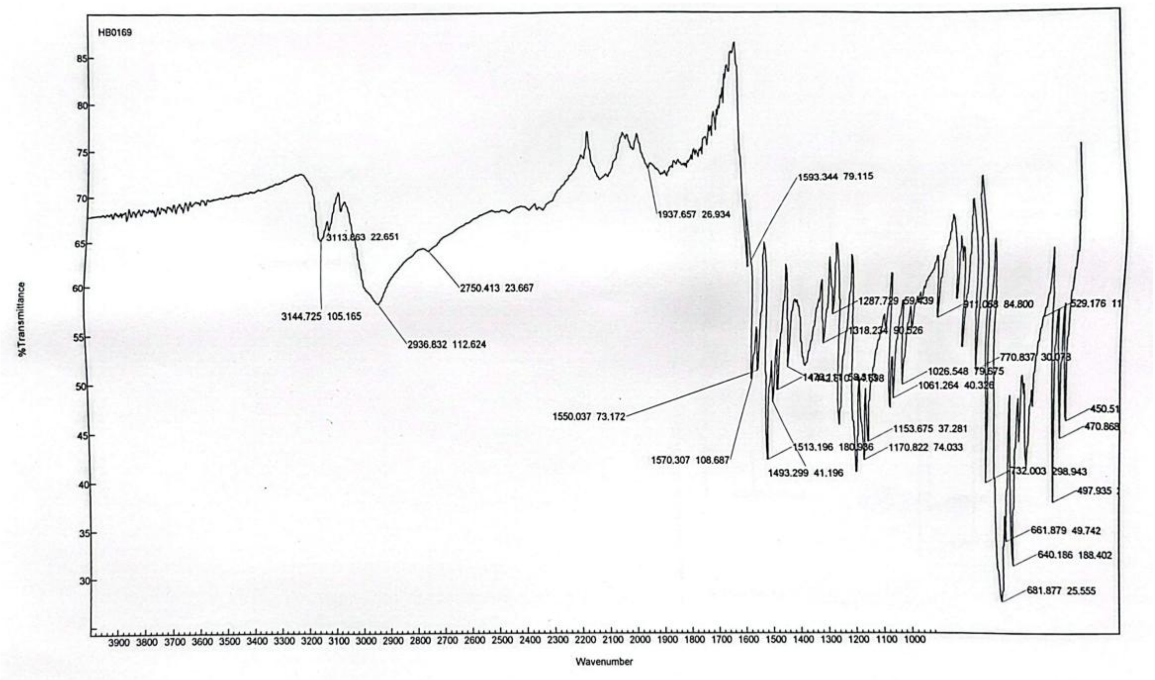

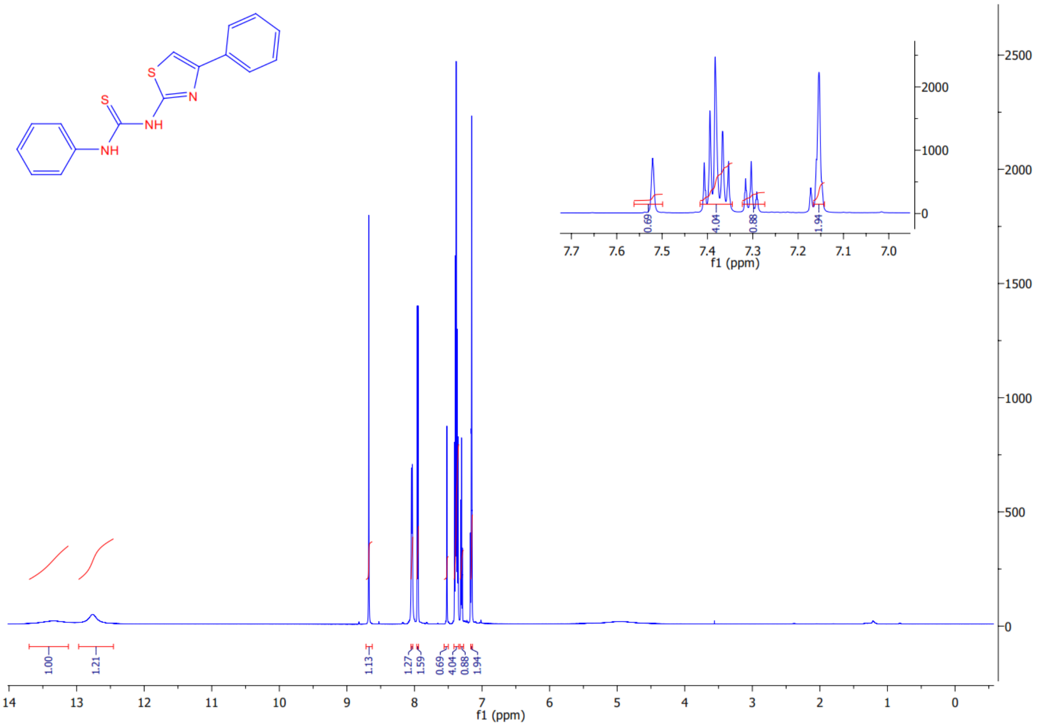

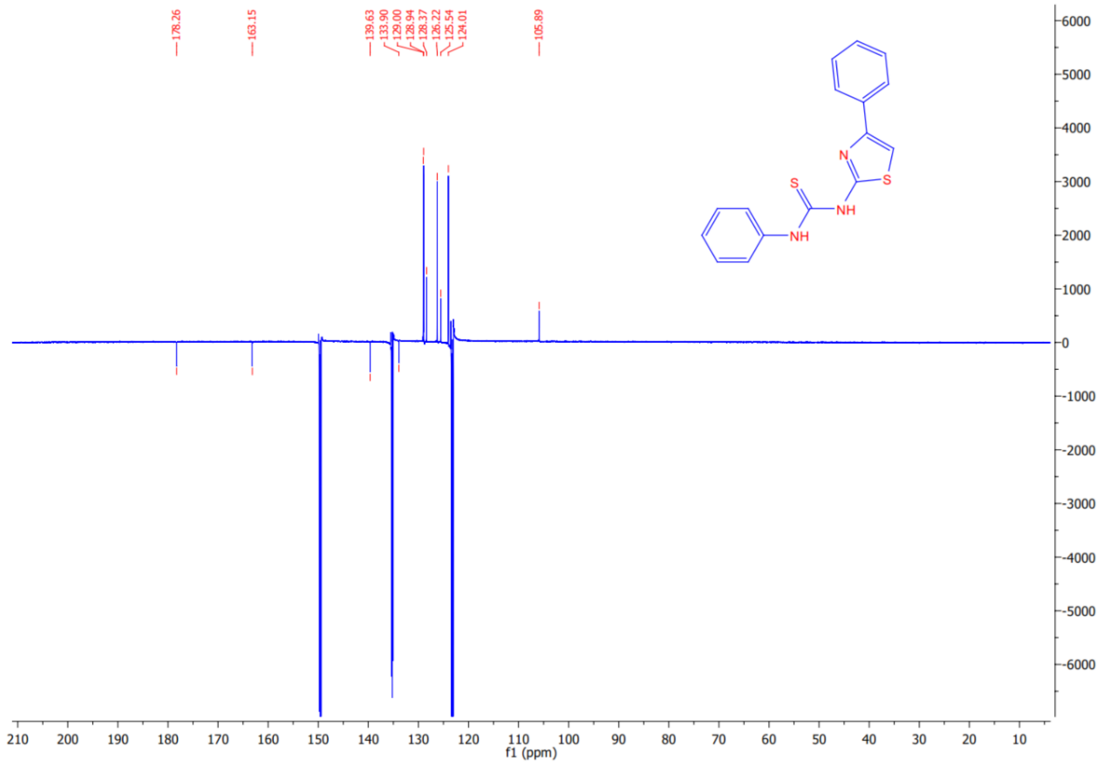
178.26
163.15
139.63
133.90
129.00
128.94
128.37
126.22
125.54
124.01
105.89
S
NH
N
S
NH
f1 (ppm)

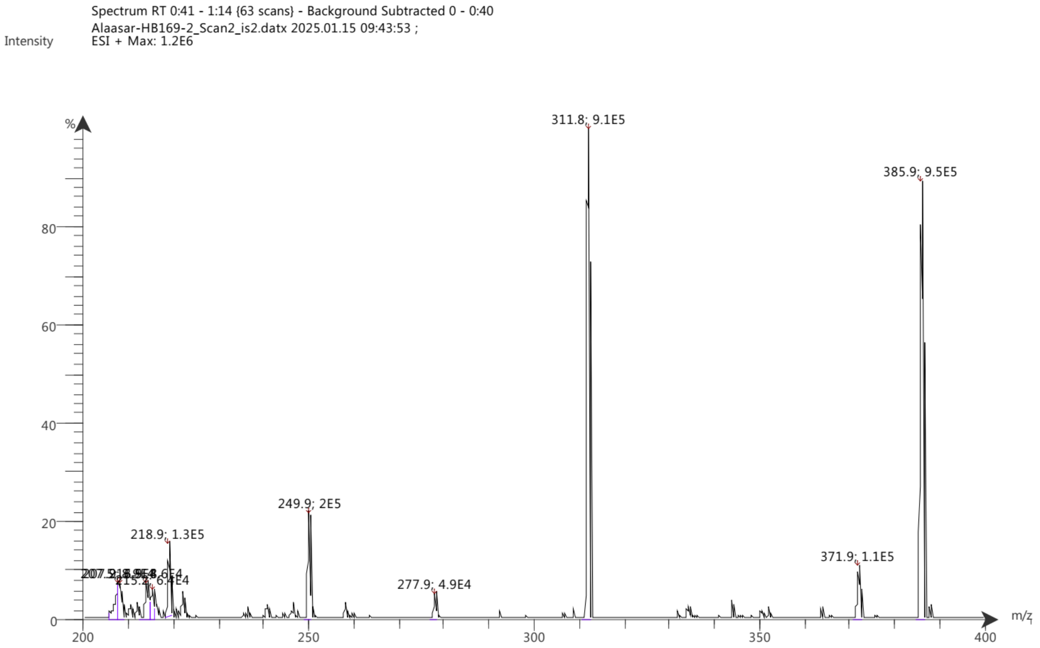
Spectrum RT 0:41 - 1:14 (63 scans) - Background Subtracted 0 - 0:40
Alaasar-HB169-2_Scan2_is2.datx 2025.01.15 09:43:53 ;
ESI + Max: 1.2E6
Intensity
311.8; 9.1E5
385.9; 9.5E5
249.9; 2E5
218.9; 1.3E5
371.9; 1.1E5
277.9; 4.9E4
215.2; 6.4E4
m/z

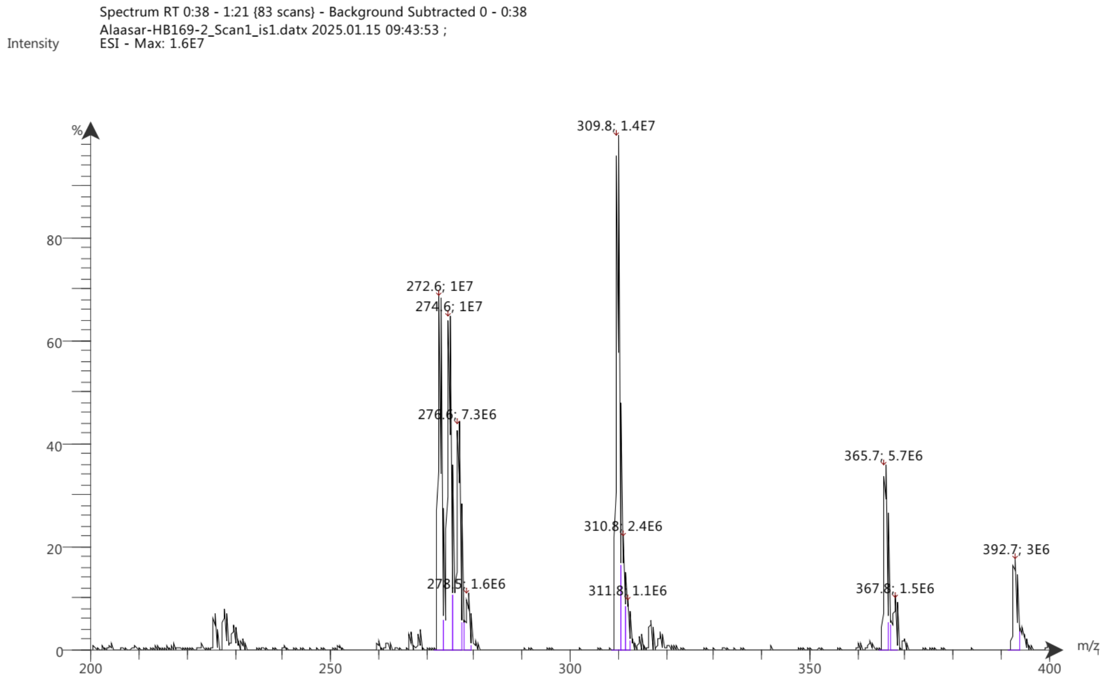
Spectrum RT 0:38 - 1:21 (83 scans) - Background Subtracted 0 - 0:38
Alaasar-HB169-2_Scan1_is1.datx 2025.01.15 09:43:53 ;
Intensity
ESI - Max: 1.6E7
%
309.8; 1.4E7
272.6; 1E7
274.6; 1E7
276.6; 7.3E6
278.5; 1.6E6
310.8; 2.4E6
311.8; 1.1E6
365.7; 5.7E6
367.8; 1.5E6
392.7; 3E6
m/z

2-(2,4-dioxothiazolidin-3-yl)-N-(4-phenylthiazol-2-yl)acetamide (HB045)

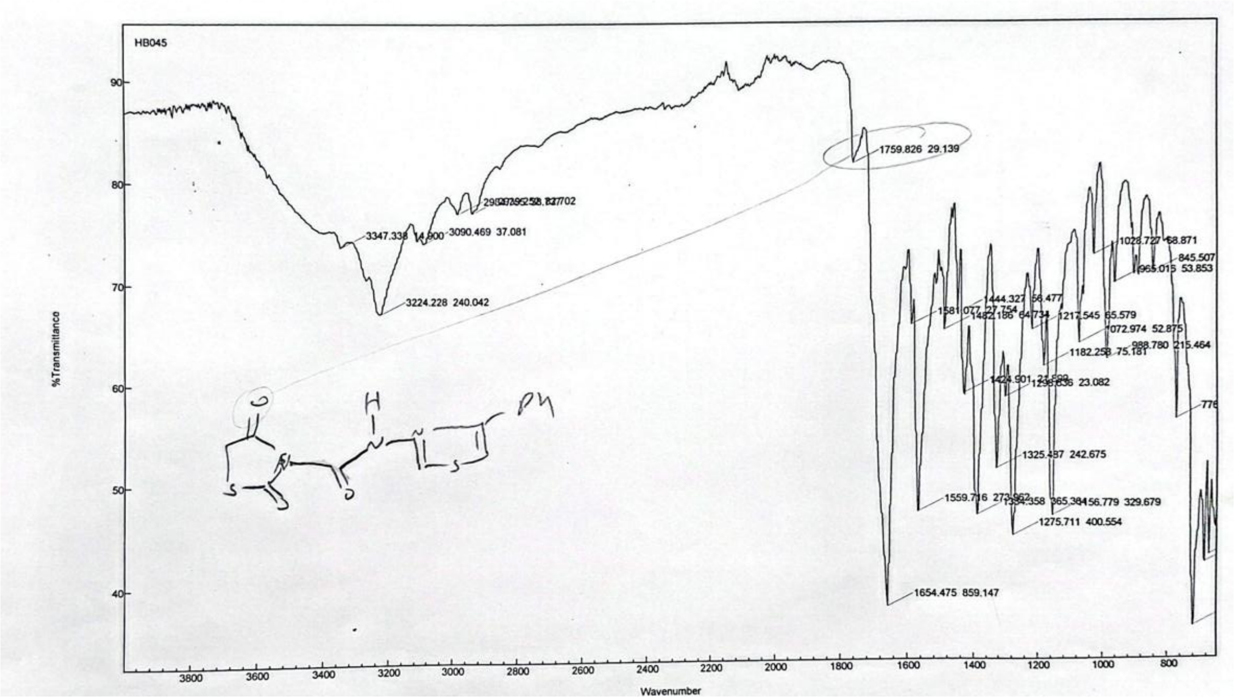

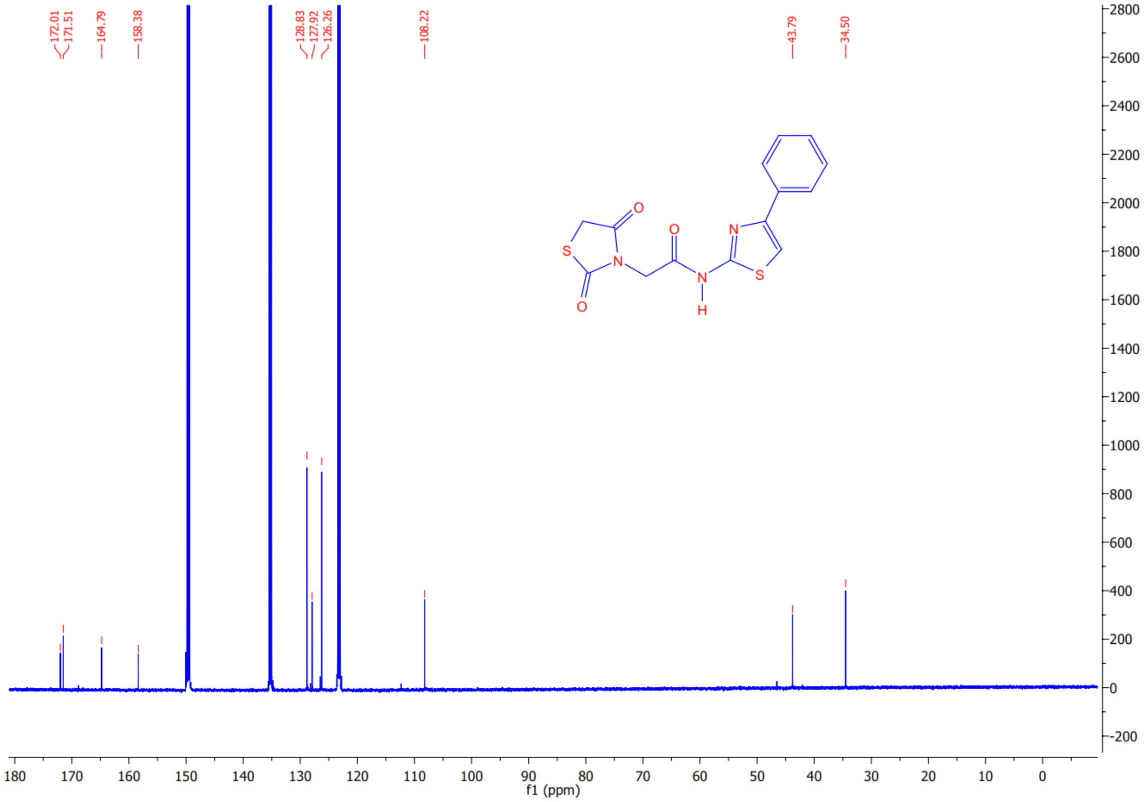

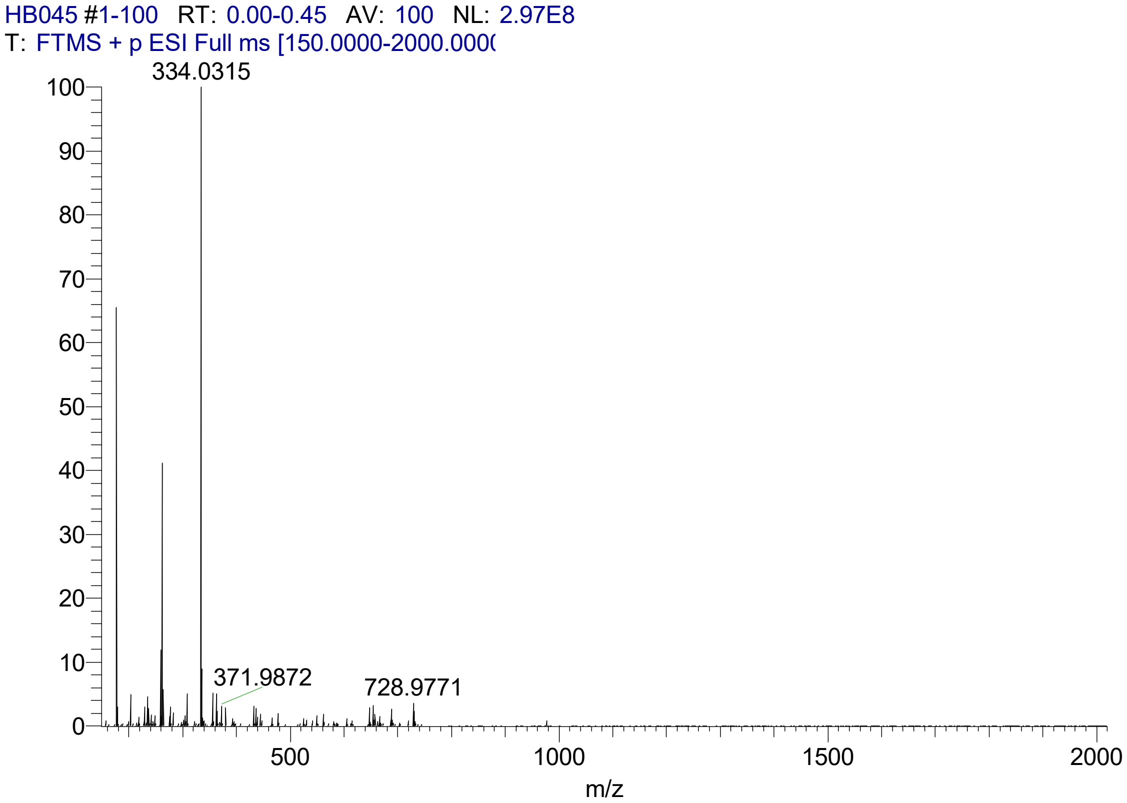
HB045 #1-100 RT: 0.00-0.45 AV: 100 NL: 2.97E8
T: FTMS + p ESI Full ms [150.0000-2000.000(
334.0315
371.9872
728.9771
m/z

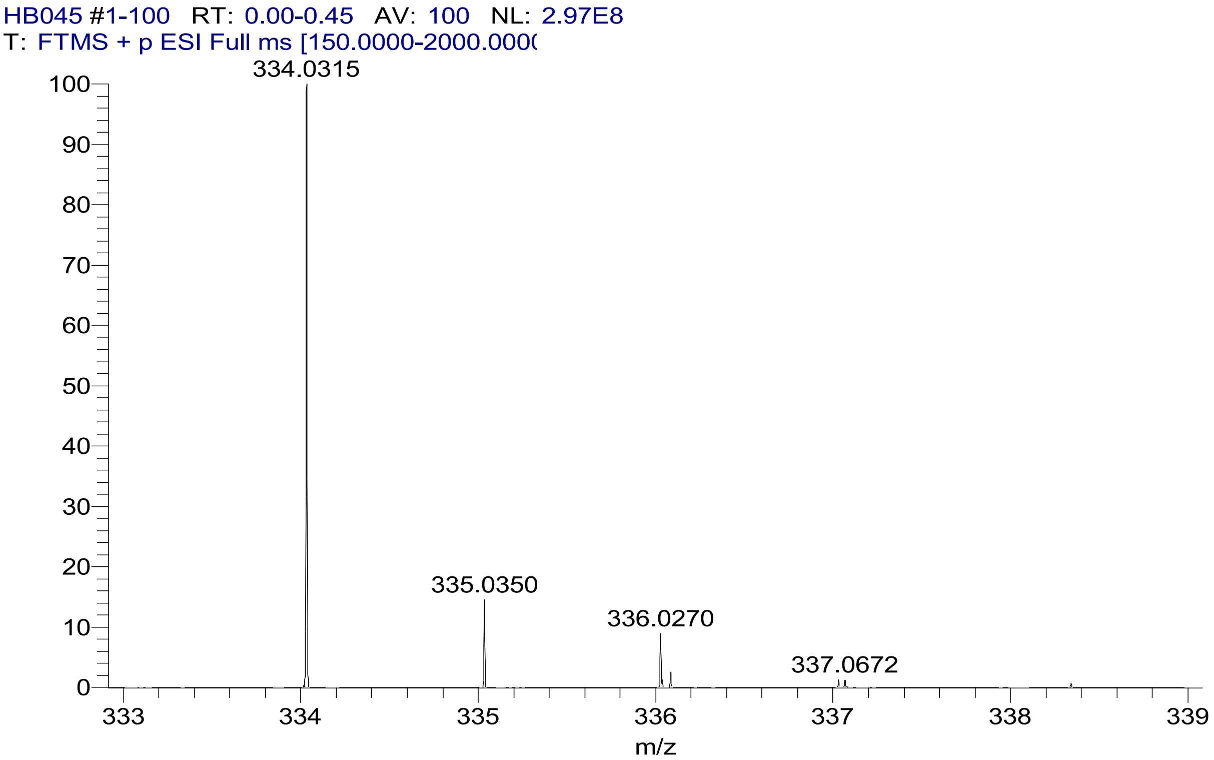
HB045 #1-100 RT: 0.00-0.45 AV: 100 NL: 2.97E8
T: FTMS + p ESI Full ms [150.0000-2000.000(
334.0315
335.0350
336.0270
337.0672
m/z

2-(5-benzylidene-2,4-dioxothiazolidin-3-yl)-N-(4-phenylthiazol-2-yl)acetamide (HB162)

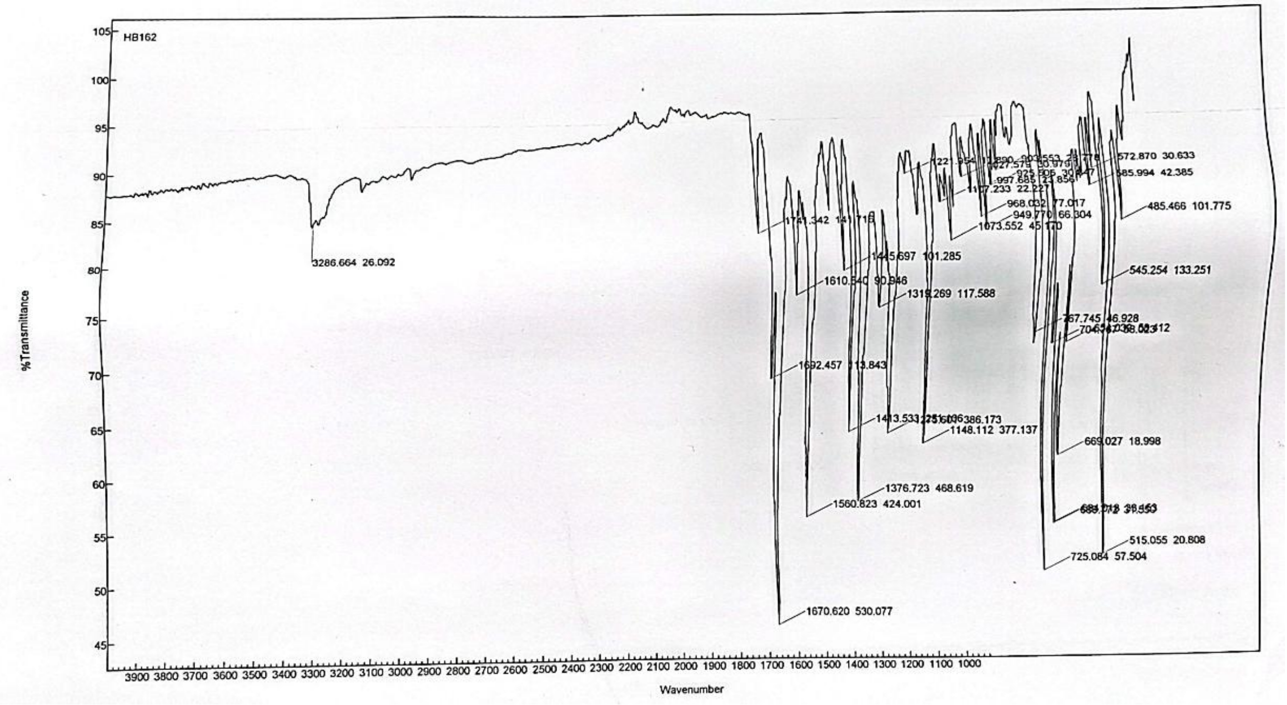

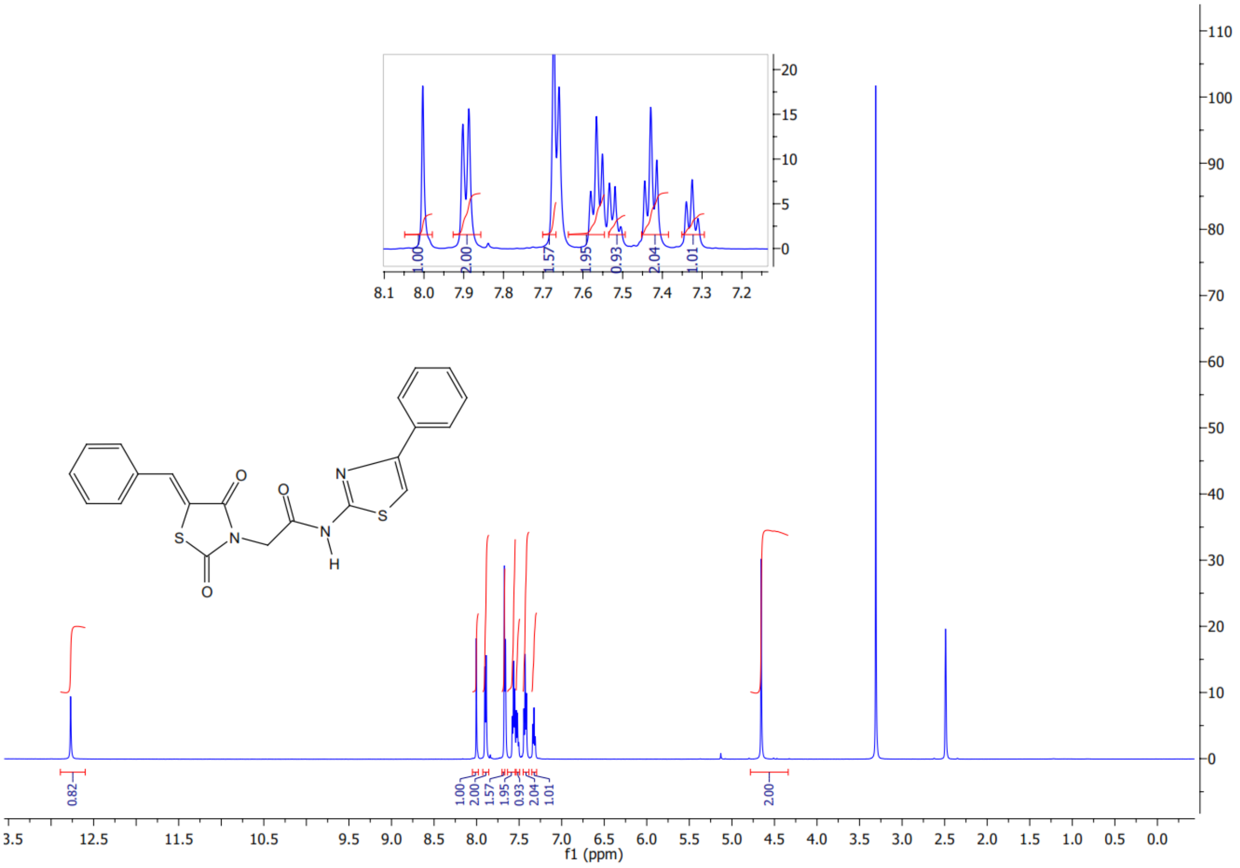

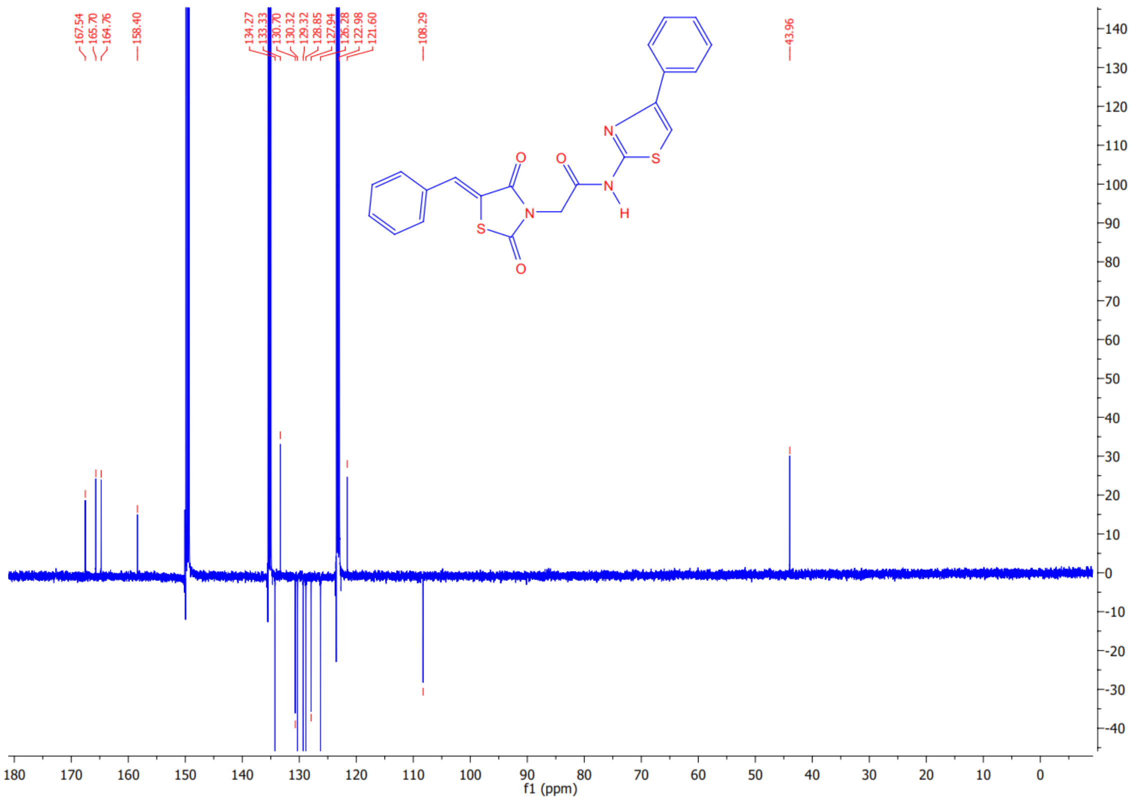
167.54
165.70
164.76
158.40
134.27
133.33
130.70
130.32
129.32
128.85
127.94
126.28
122.98
121.60
108.29
43.96
O
O
N
S
N
S
N
H
O
O
f1 (ppm)

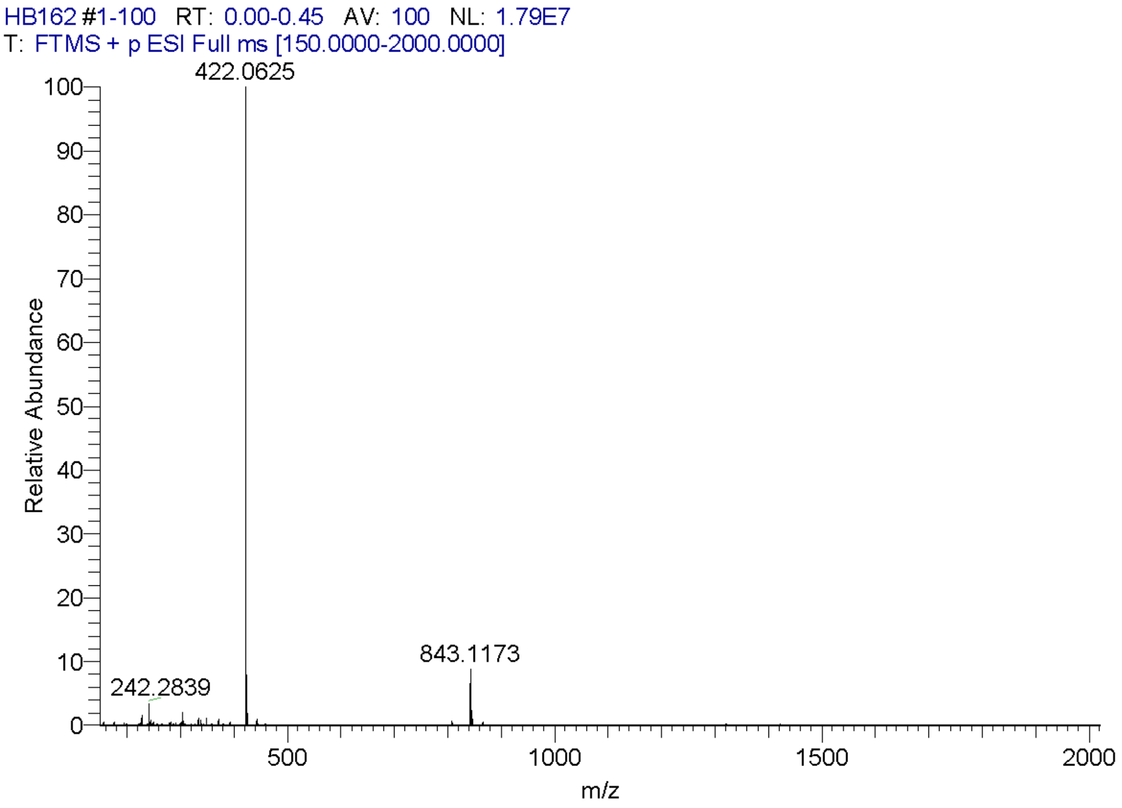
HB162 #1-100 RT: 0.00-0.45 AV: 100 NL: 1.79E7
T: FTMS + p ESI Full ms [150.0000-2000.0000]
422.0625
843.1173
242.2839
Relative Abundance
m/z

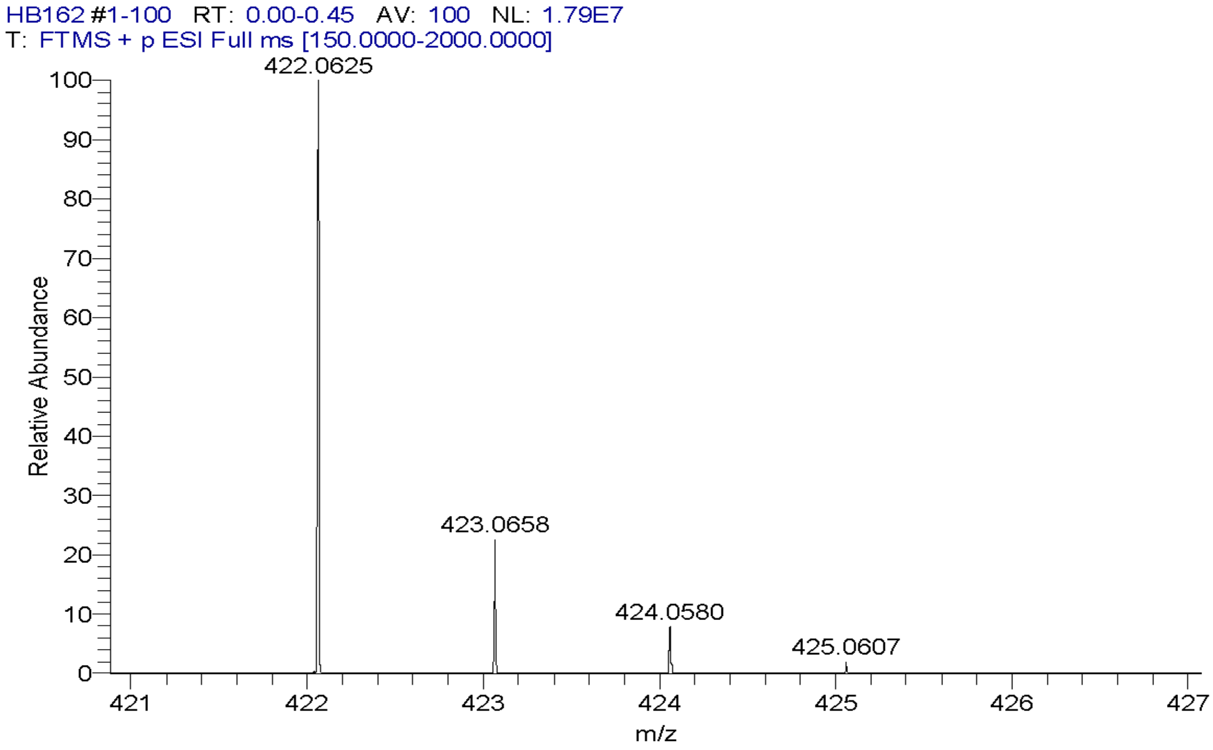

HB162 #1-100 RT: 0.00-0.45 AV: 100 NL: 1.79E7
T: FTMS + p ESI Full ms [150.0000-2000.0000]
422.0625
423.0658
424.0580
425.0607
Relative Abundance
m/z

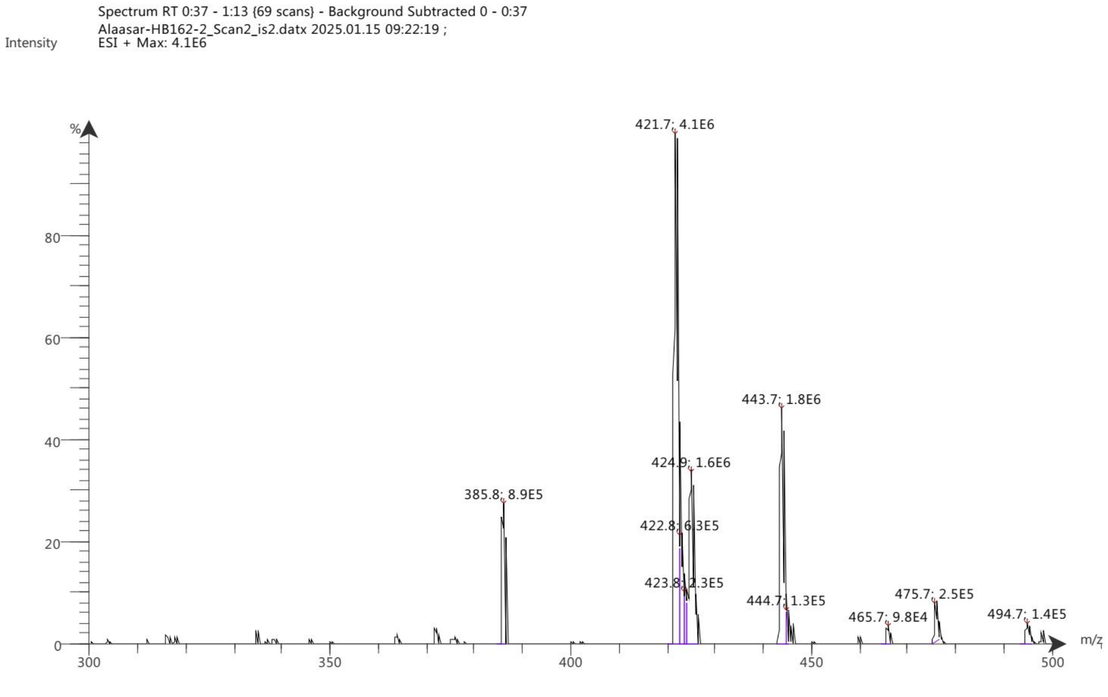

Spectrum RT 0:37 - 1:13 (69 scans) - Background Subtracted 0 - 0:37
Alaasar-HB162-2_Scan2_is2.datx 2025.01.15 09:22:19 ;
ESI + Max: 4.1E6
Intensity
421.7; 4.1E6
443.7; 1.8E6
424.9; 1.6E6
385.8; 8.9E5
422.8; 6.3E5
423.8; 2.3E5
444.7; 1.3E5
475.7; 2.5E5
465.7; 9.8E4
494.7; 1.4E5
m/z

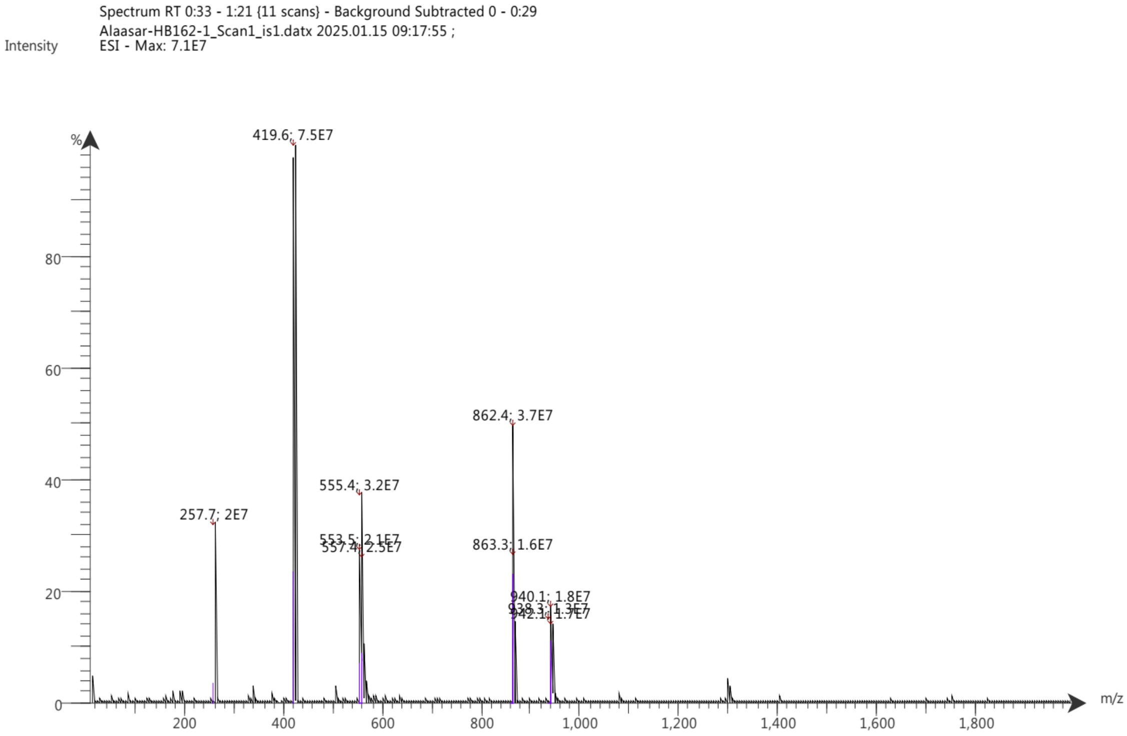
Spectrum RT 0:33 - 1:21 (11 scans) - Background Subtracted 0 - 0:29
Alaasar-HB162-1_Scan1_is1.datx 2025.01.15 09:17:55 ;
Intensity
ESI - Max: 7.1E7
%
419.6; 7.5E7
862.4; 3.7E7
555.4; 3.2E7
257.7; 2E7
553.5; 2.1E7
557.4; 2.5E7
863.3; 1.6E7
940.1; 1.8E7
m/z
0
20
40
60
80
200
400
600
800
1,000
1,200
1,400
1,600
1,800

2-(5-(4-bromobenzylidene)-2,4-dioxothiazolidin-3-yl)-N-(4-phenylthiazol-2-yl)acetamide (HB161)

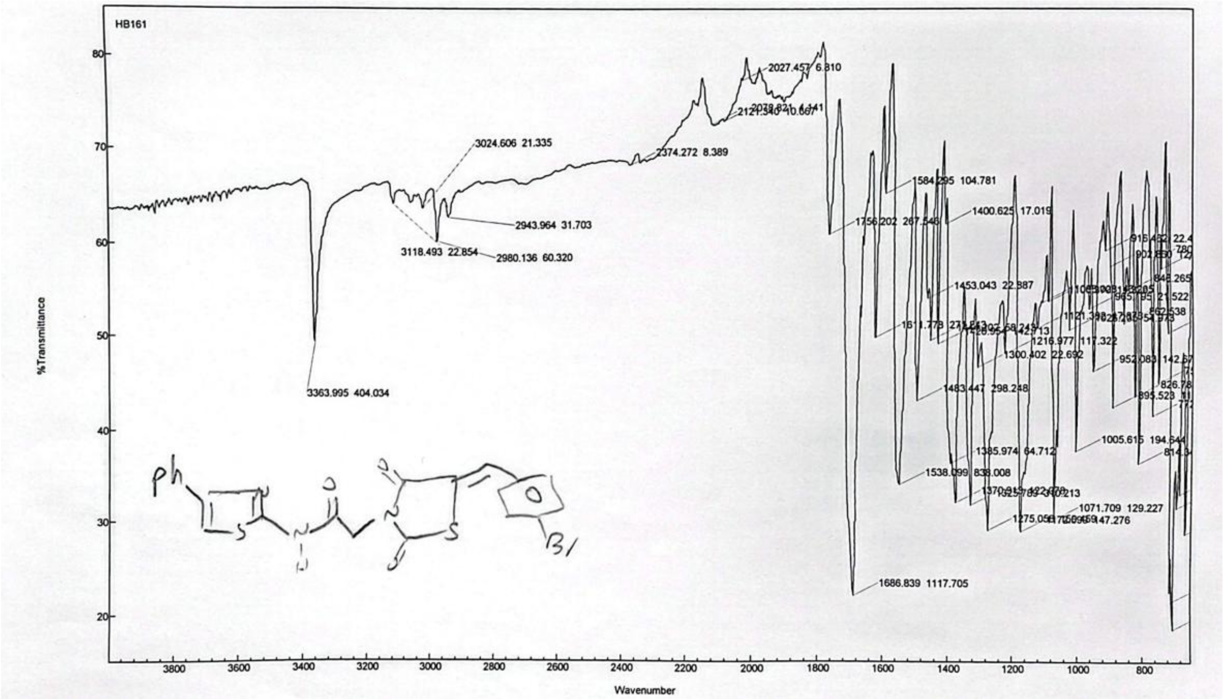

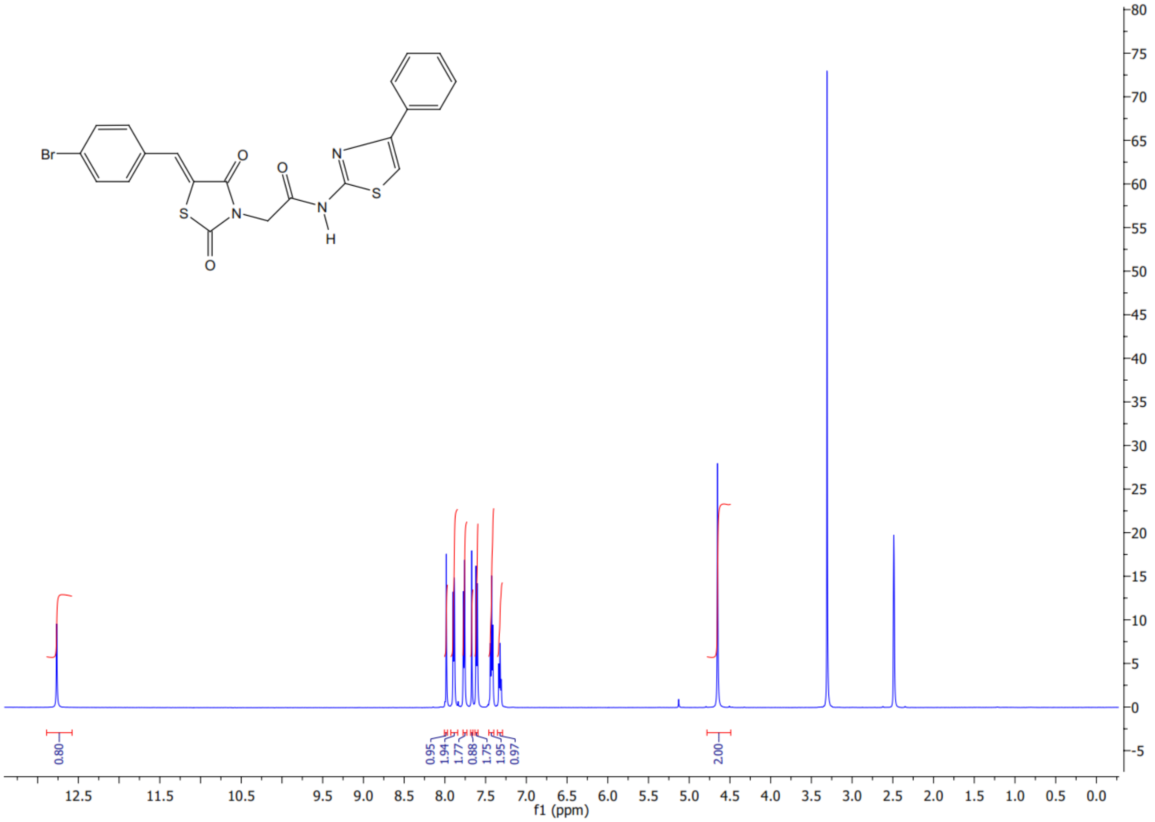

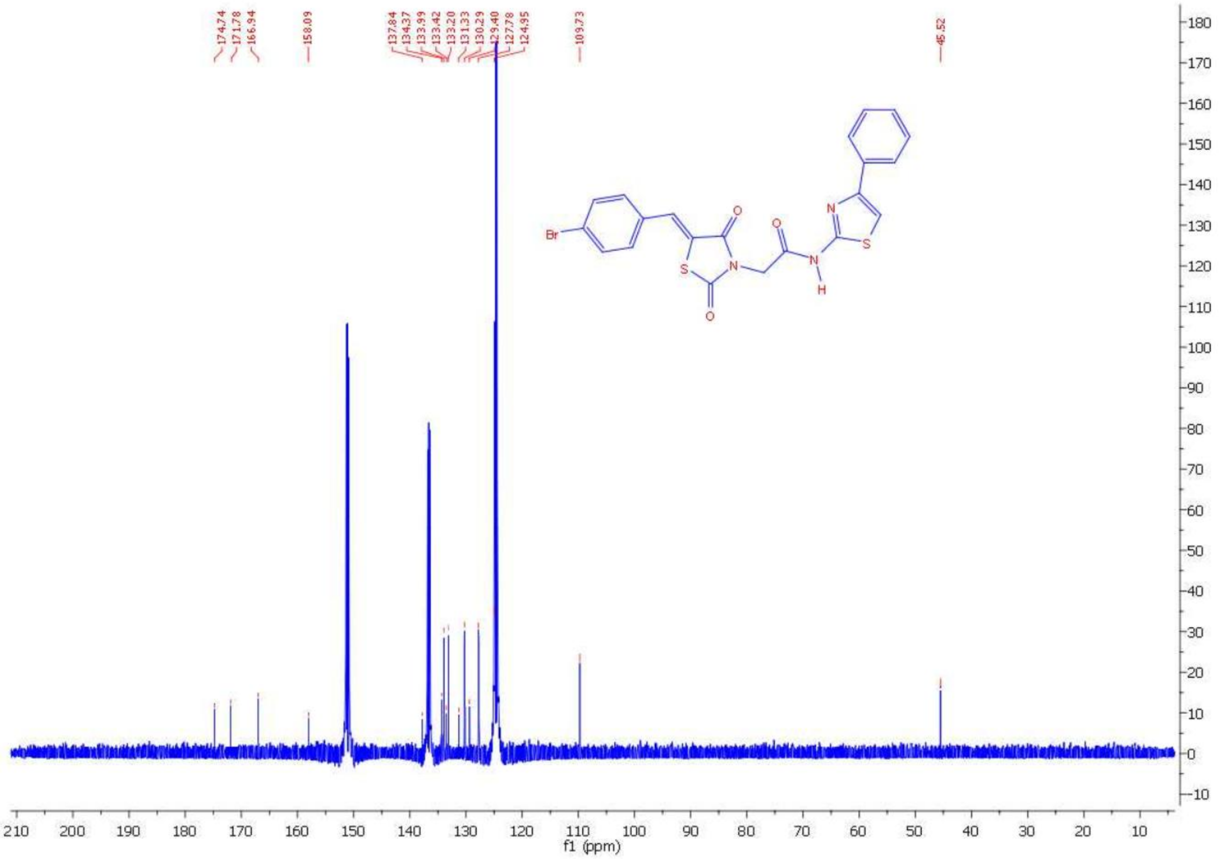
174.74
171.78
166.94
158.09
137.84
134.37
133.99
133.42
133.20
131.33
130.29
129.40
127.78
124.95
109.73
45.52
Br
O
S
N
O
O
N
H
N
S
f1 (ppm)

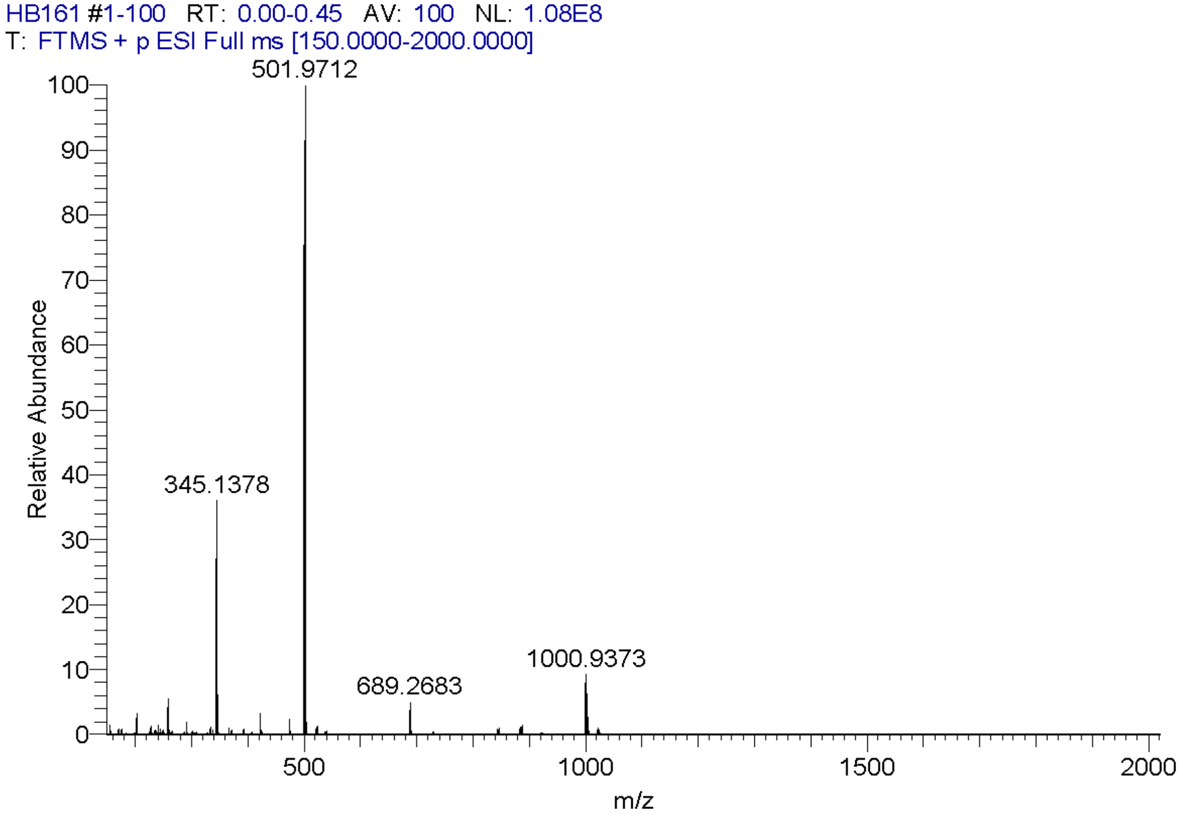
HB161 #1-100 RT: 0.00-0.45 AV: 100 NL: 1.08E8
T: FTMS + p ESI Full ms [150.0000-2000.0000]
501.9712
345.1378
689.2683
1000.9373
Relative Abundance
m/z

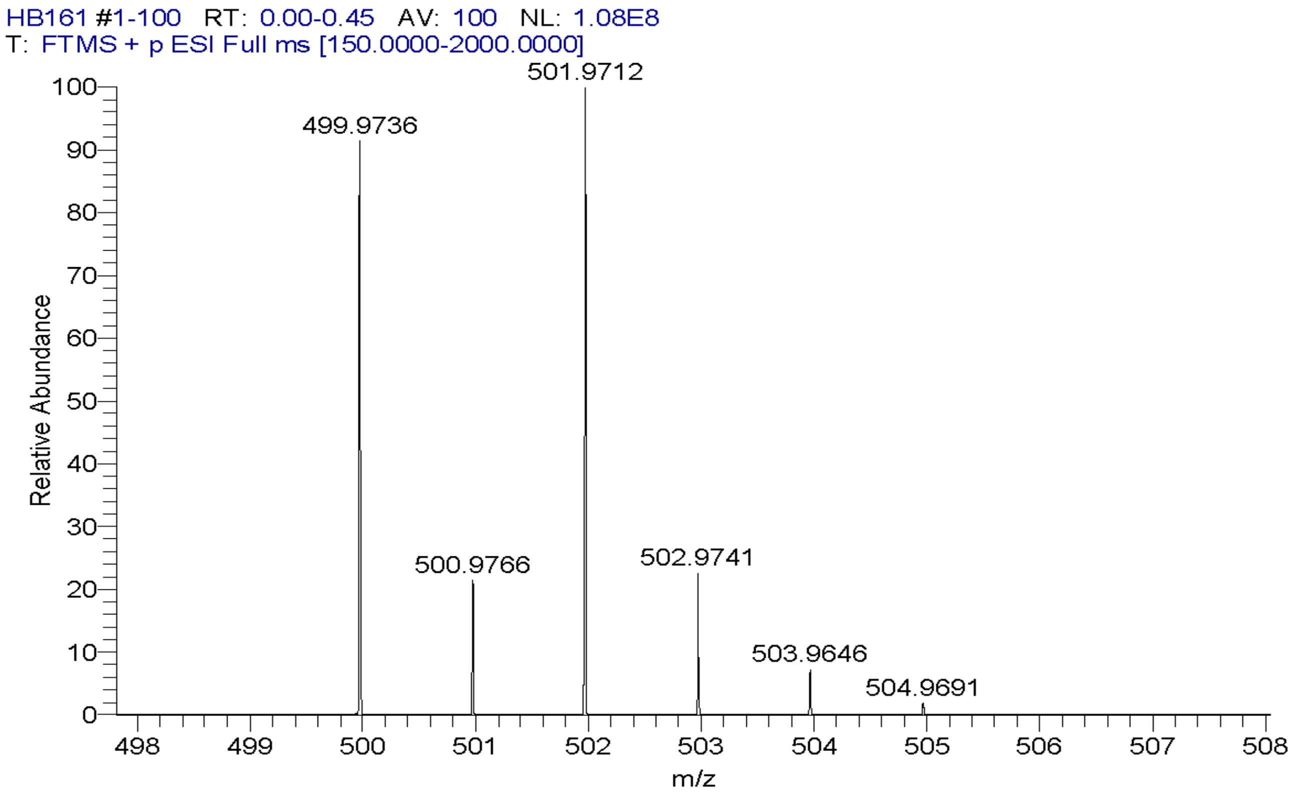
HB161 #1-100 RT: 0.00-0.45 AV: 100 NL: 1.08E8
T: FTMS + p ESI Full ms [150.0000-2000.0000]
501.9712
499.9736
500.9766
502.9741
503.9646
504.9691
Relative Abundance
m/z
100
90
80
70
60
50
40
30
20
10
0
498
499
500
501
502
503
504
505
506
507
508

Synthesis of 2-(5-(4-chlorobenzylidene)-2,4-dioxothiazolidin-3-yl)-N-(4-phenylthiazol-2-yl)acetamide (HB163)

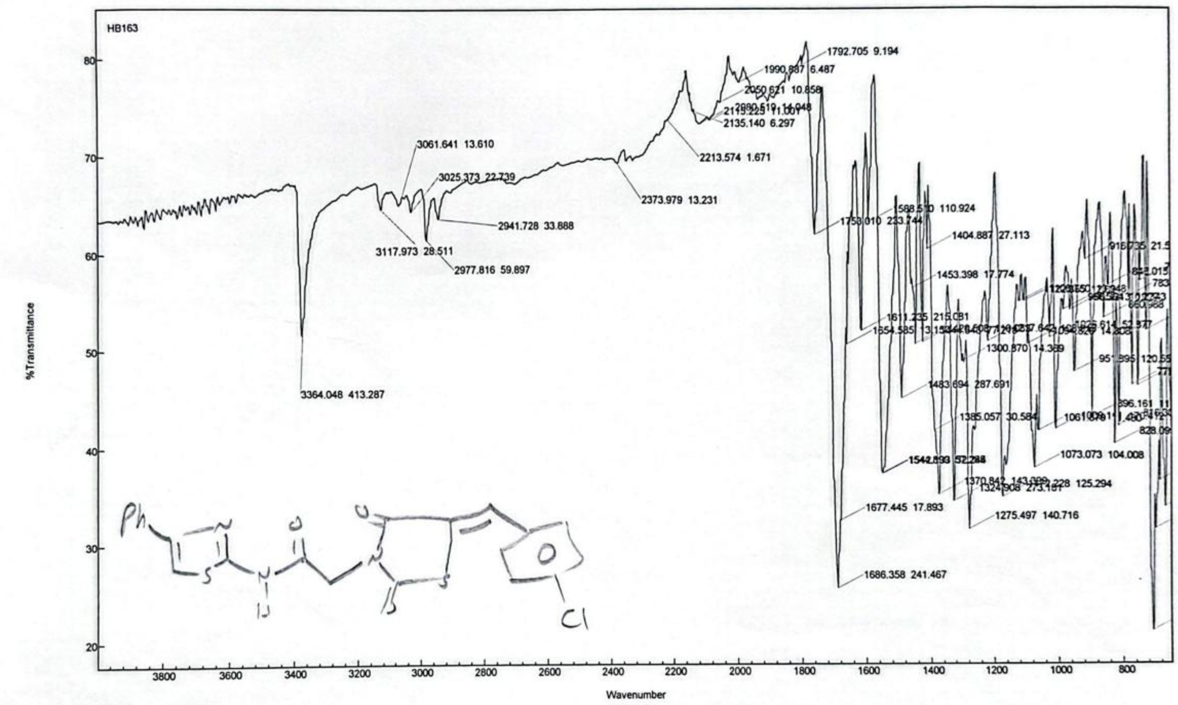

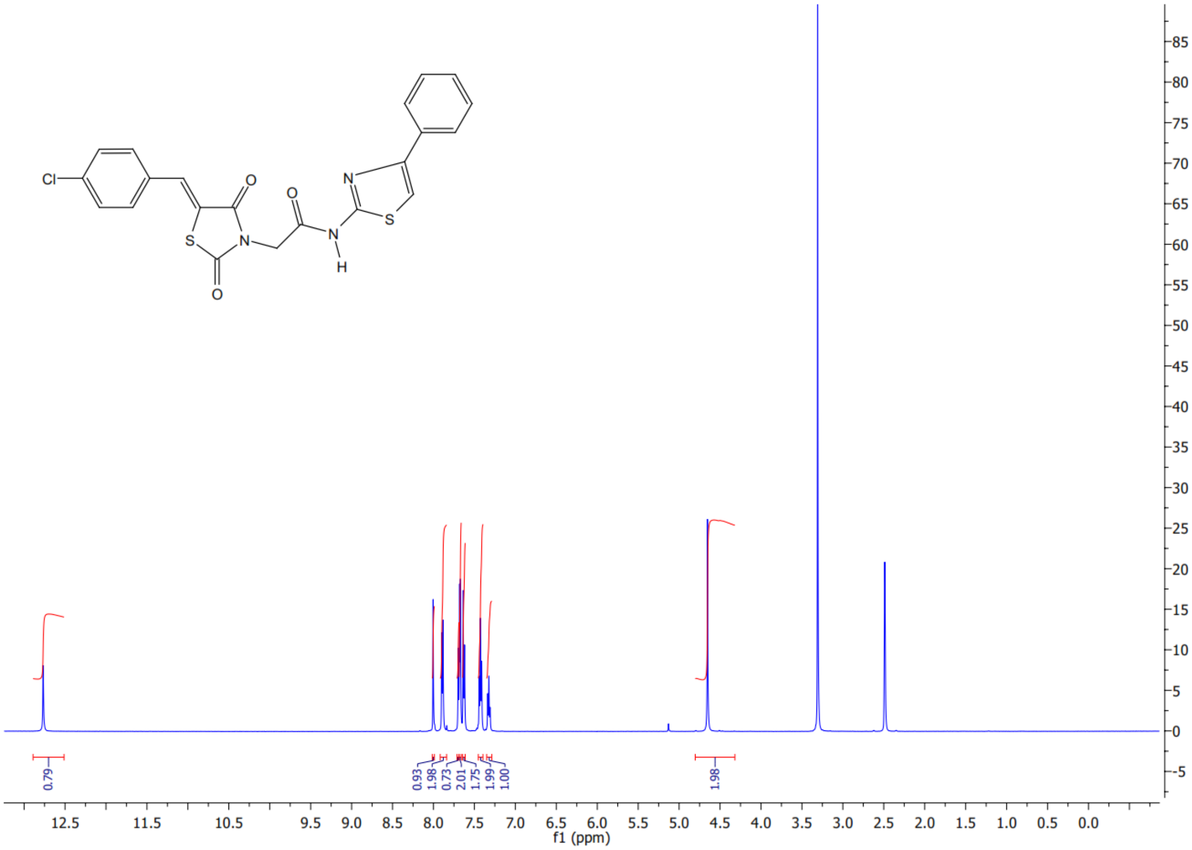

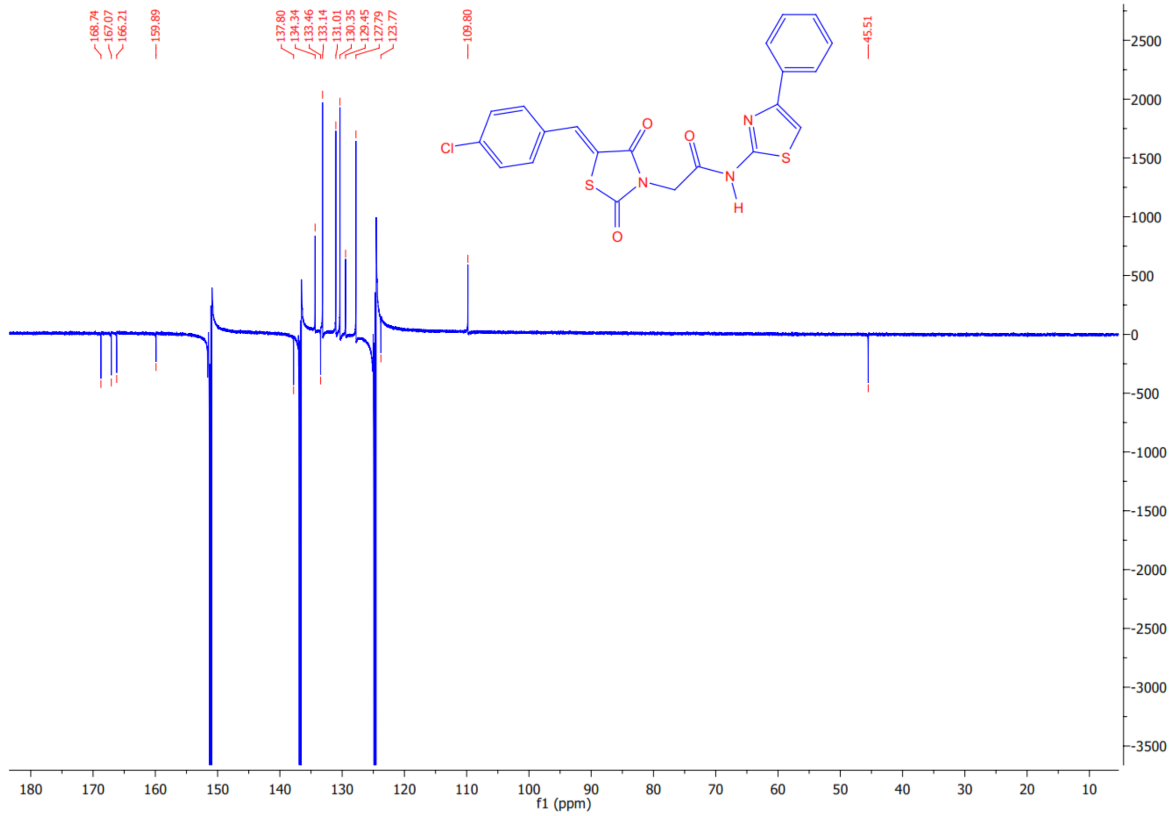
168.74
167.07
166.21
159.89
137.80
134.34
133.46
133.14
131.01
130.35
129.45
127.79
123.77
109.80
45.51
Cl
O
O
N
S
N
H
N
S
O
f1 (ppm)

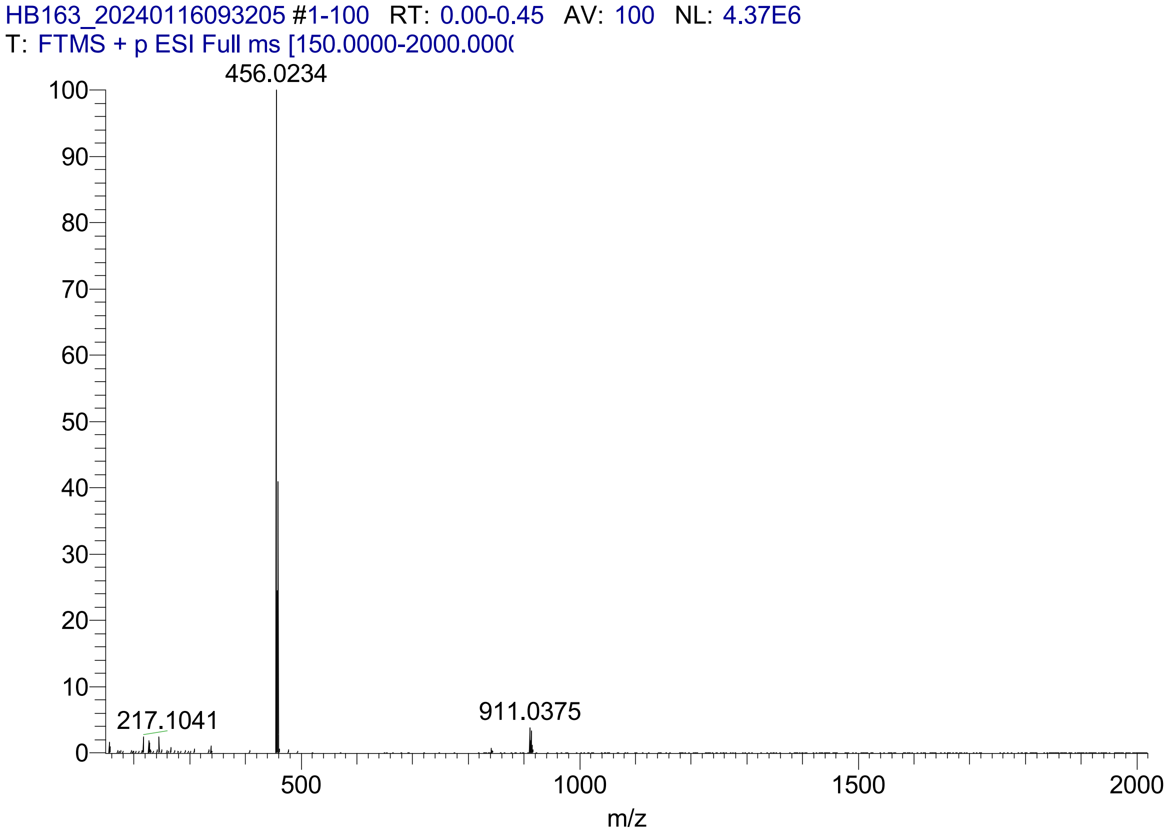
HB163_20240116093205 #1-100 RT: 0.00-0.45 AV: 100 NL: 4.37E6
T: FTMS + p ESI Full ms [150.0000-2000.000(
456.0234
217.1041
911.0375
m/z

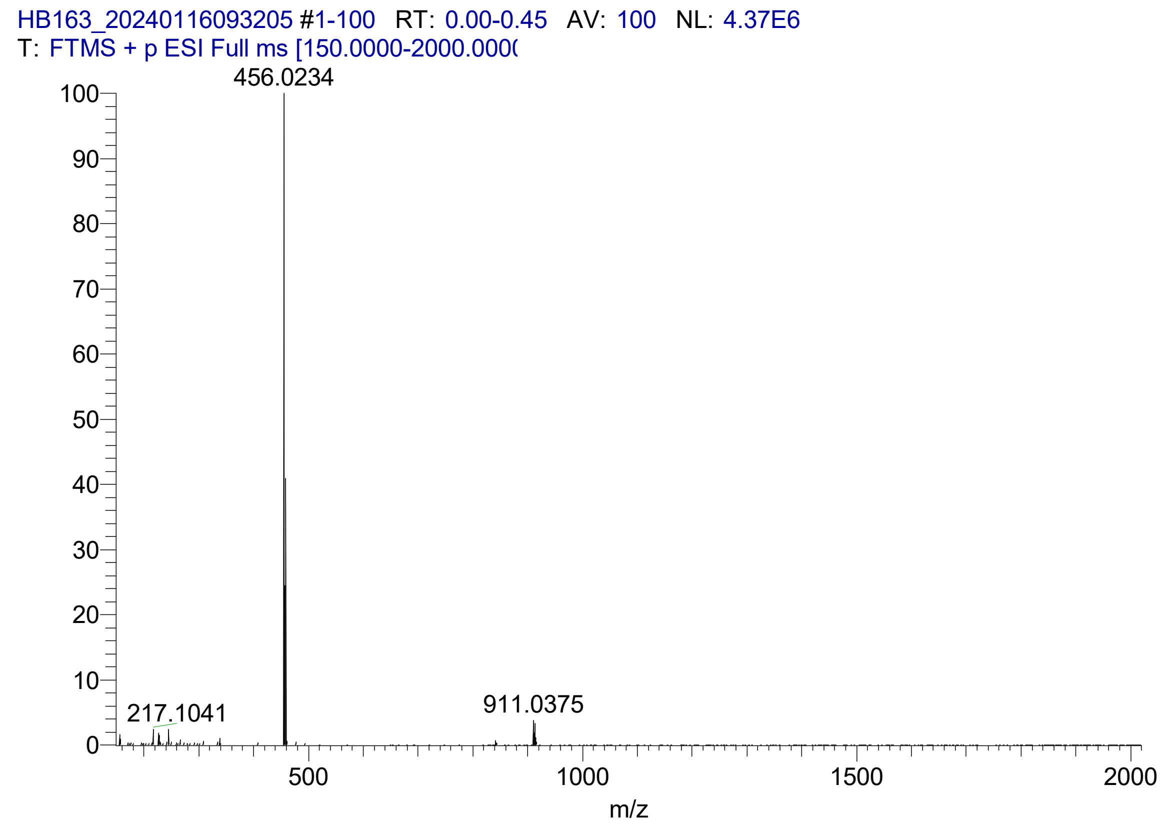
HB163_20240116093205 #1-100 RT: 0.00-0.45 AV: 100 NL: 4.37E6
T: FTMS + p ESI Full ms [150.0000-2000.000(
456.0234
217.1041
911.0375
m/z

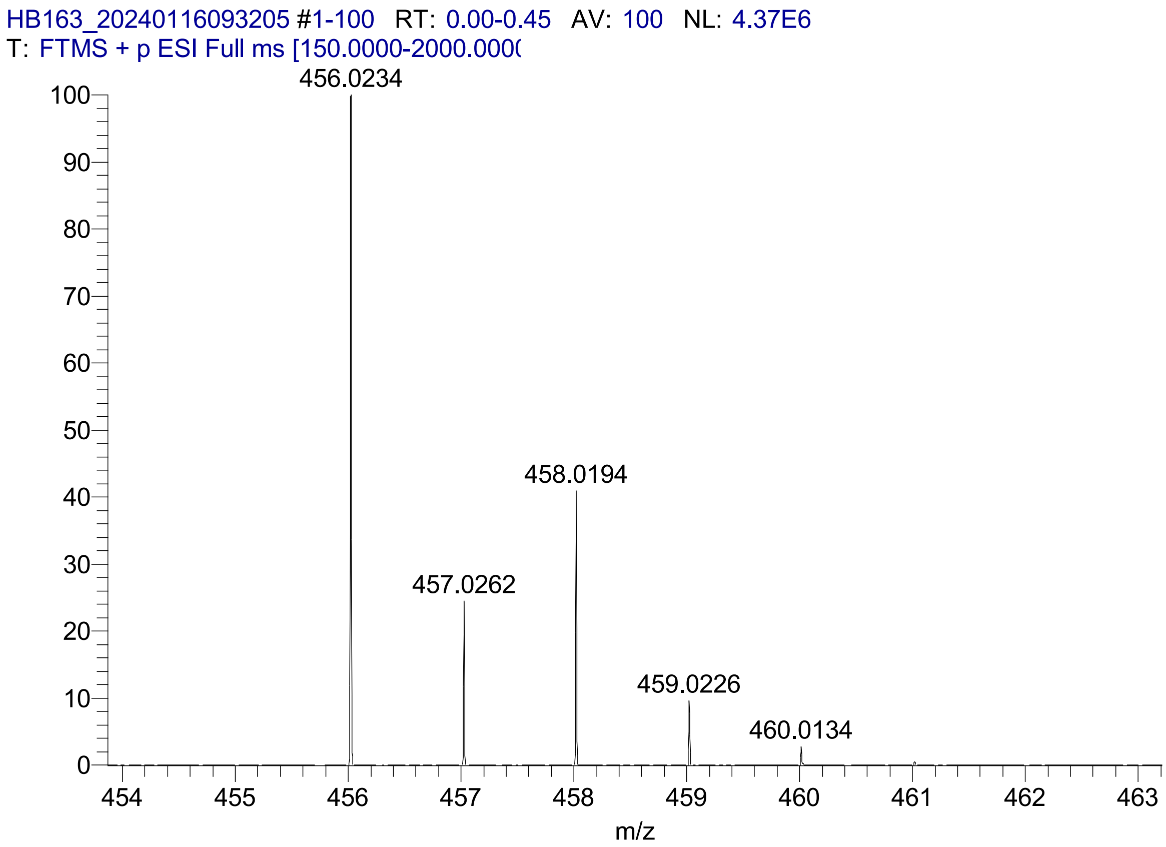
HB163_20240116093205 #1-100 RT: 0.00-0.45 AV: 100 NL: 4.37E6
T: FTMS + p ESI Full ms [150.0000-2000.000(
456.0234
458.0194
457.0262
459.0226
460.0134
m/z

2-(5-(4-fluorobenzylidene)-2,4-dioxothiazolidin-3-yl)-N-(4-phenylthiazol-2-yl)acetamide (HB121)

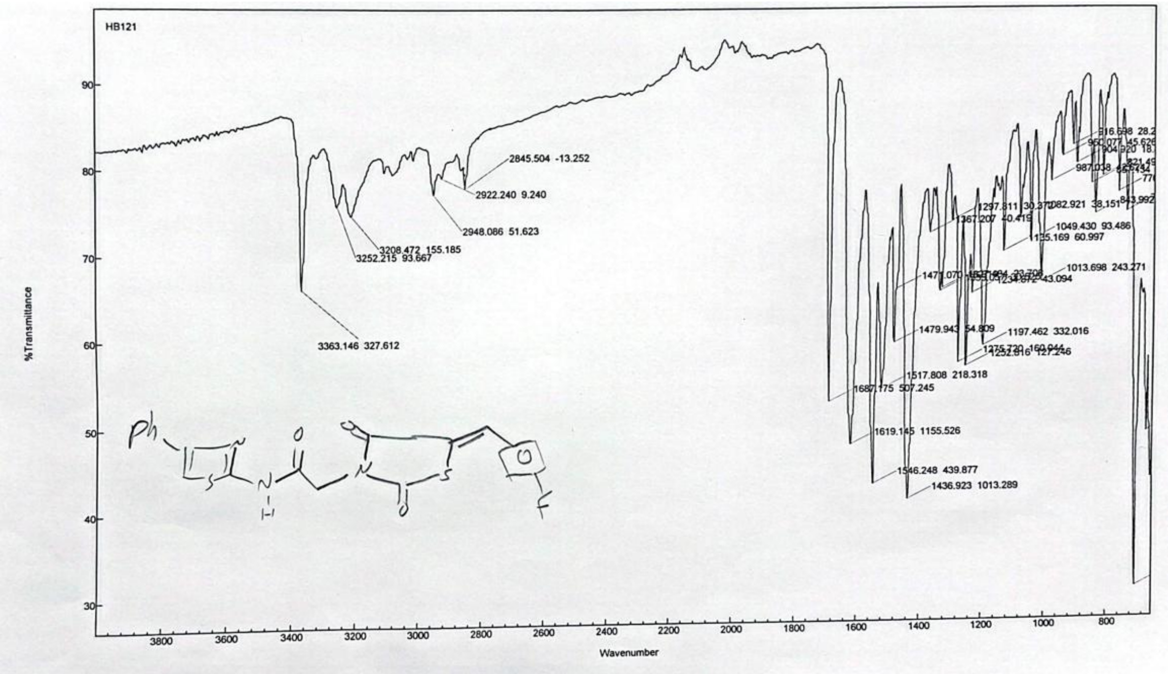

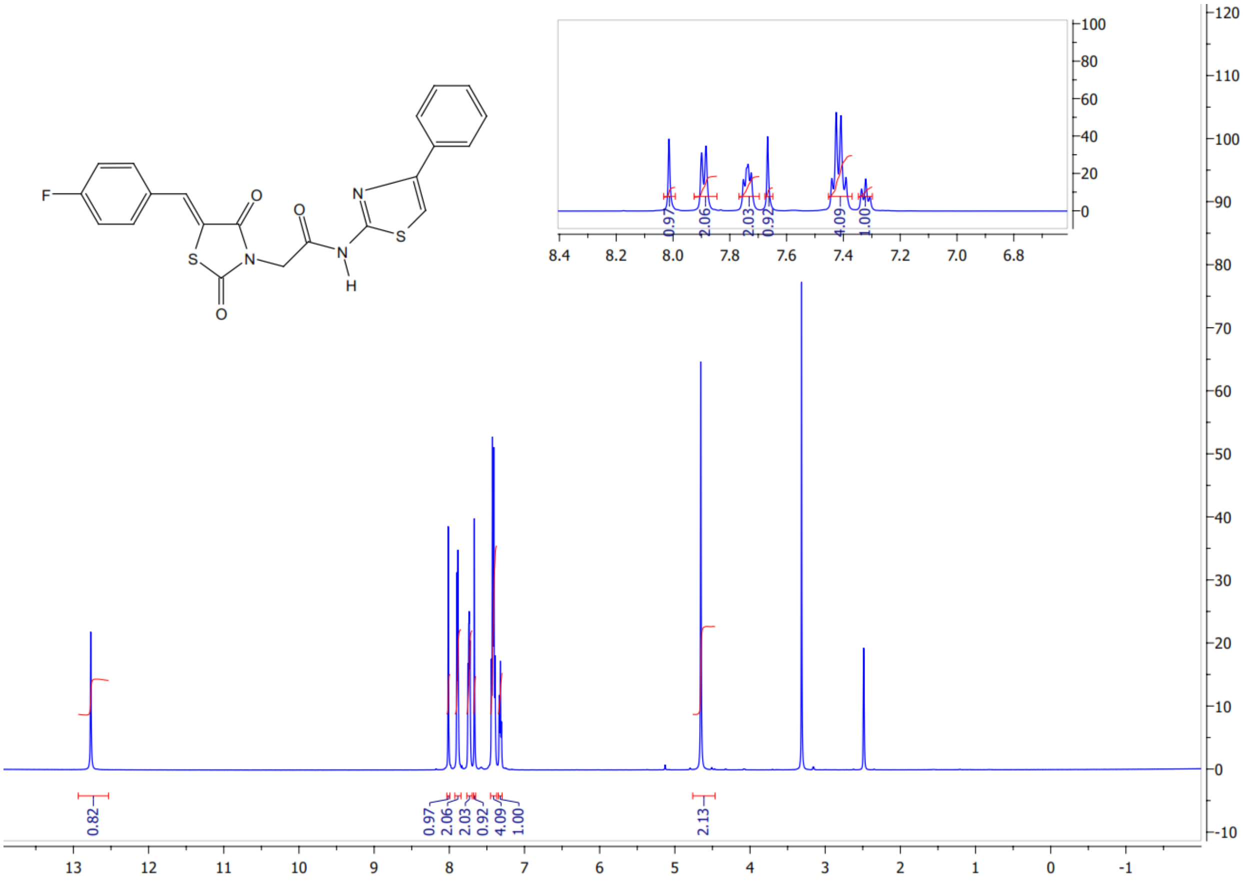

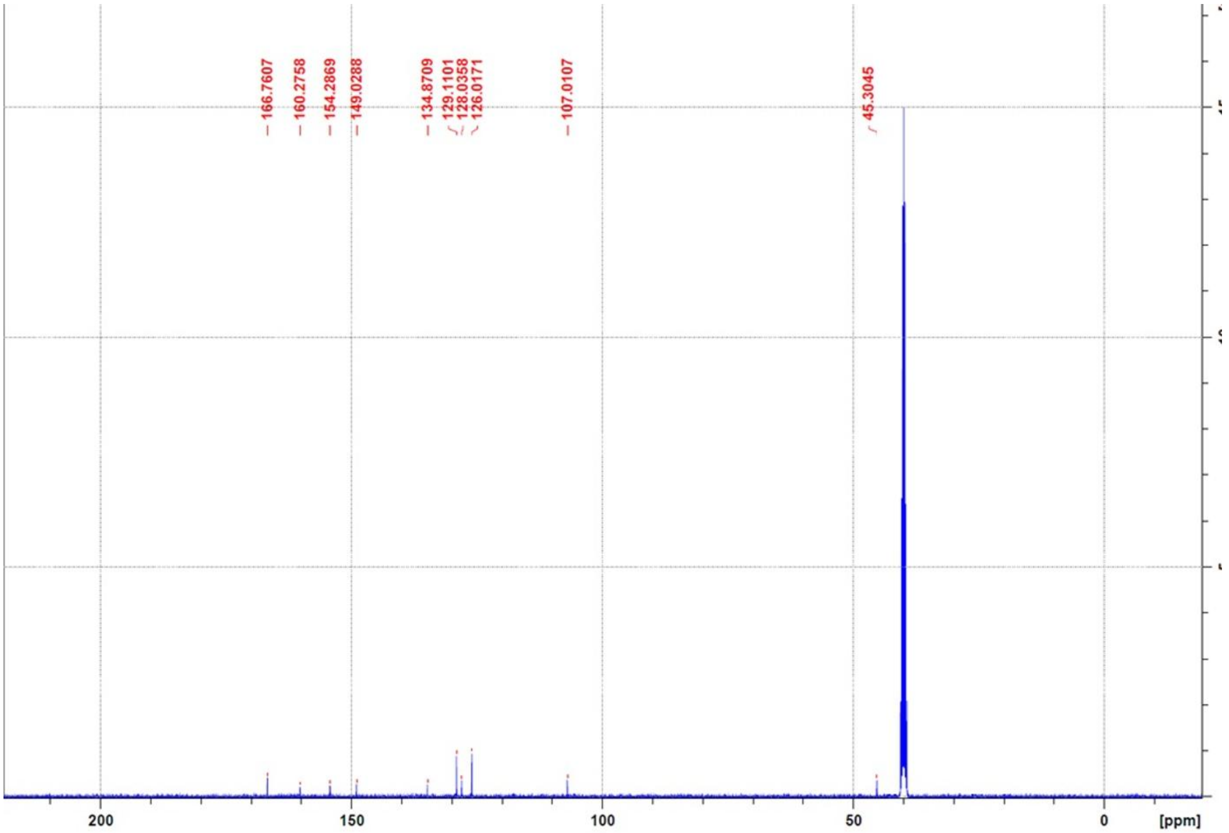
166.7607
160.2758
154.2869
149.0288
134.8709
129.1101
128.0358
126.0171
107.0107
45.3045
200
150
100
50
0
[ppm]

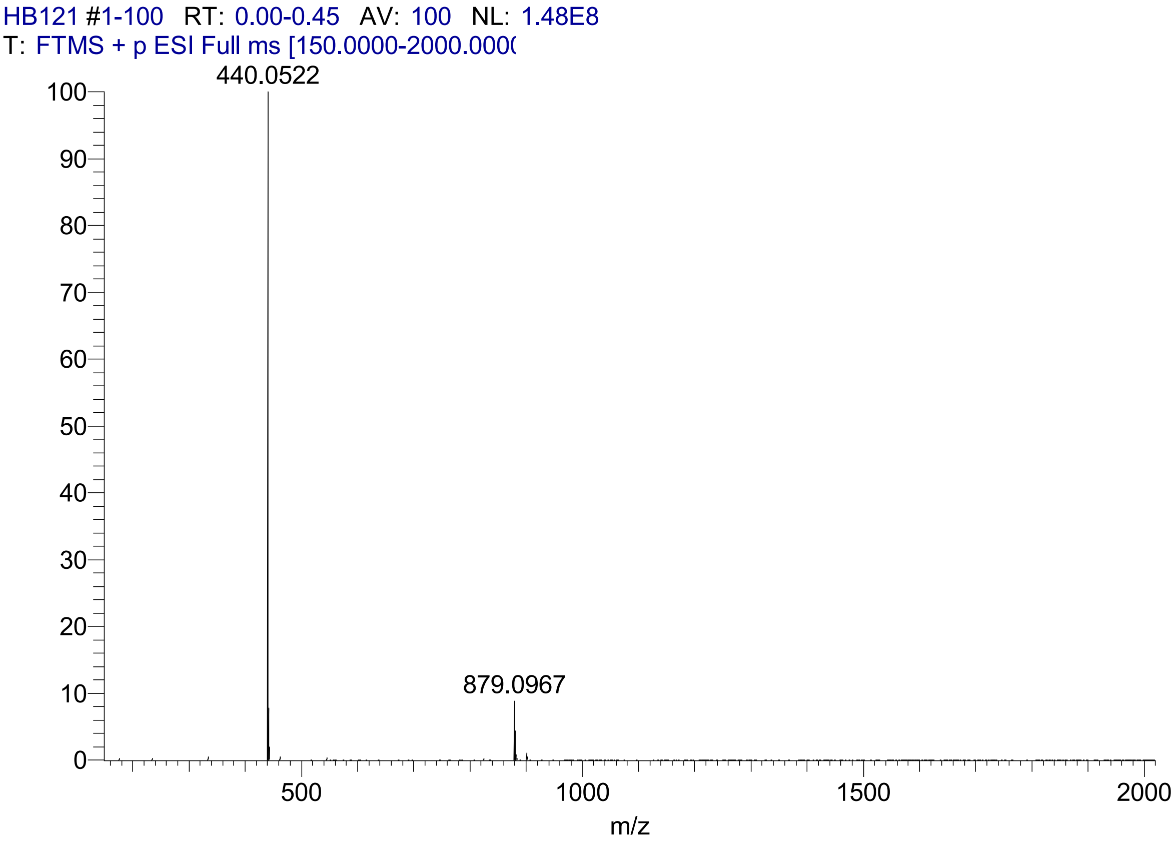
HB121 #1-100 RT: 0.00-0.45 AV: 100 NL: 1.48E8
T: FTMS + p ESI Full ms [150.0000-2000.000(
440.0522
879.0967
100
90
80
70
60
50
40
30
20
10
0
500
1000
1500
2000
m/z

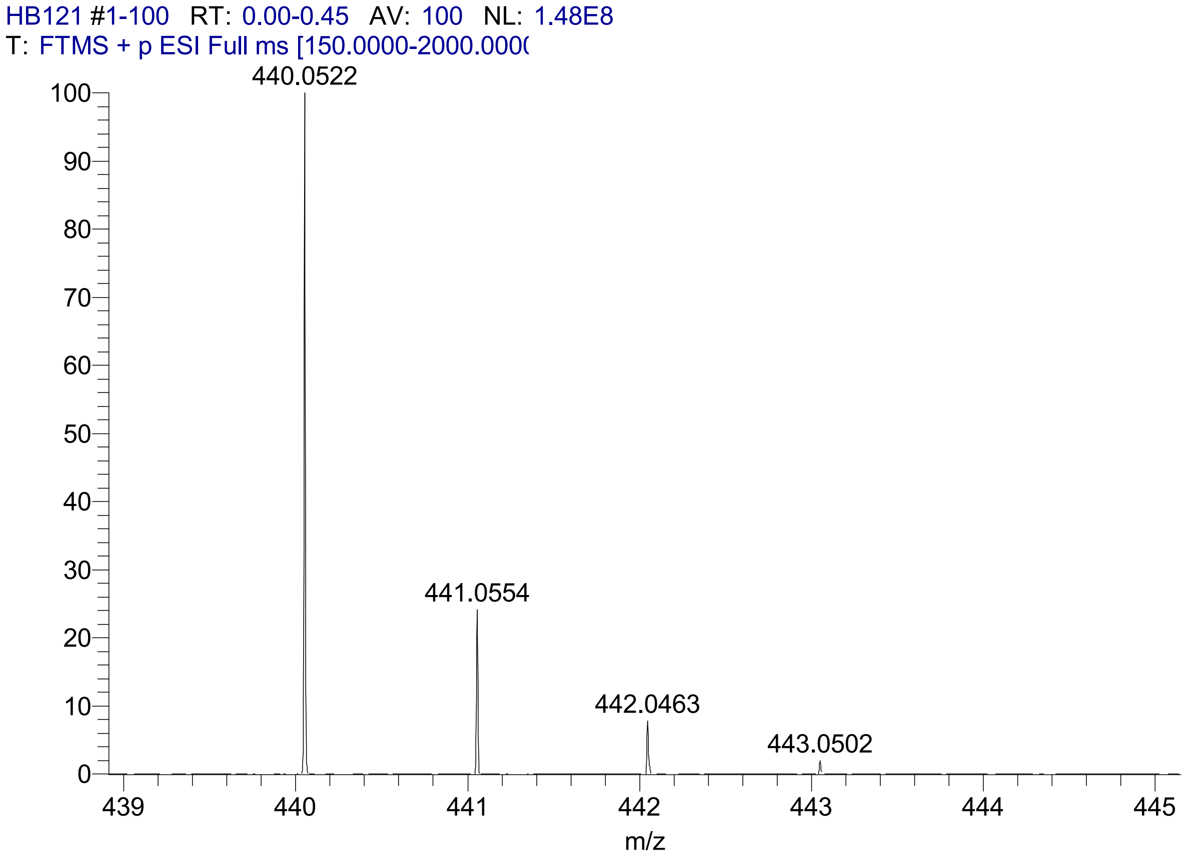
HB121 #1-100 RT: 0.00-0.45 AV: 100 NL: 1.48E8
T: FTMS + p ESI Full ms [150.0000-2000.000(
440.0522
441.0554
442.0463
443.0502
100
90
80
70
60
50
40
30
20
10
0
439
440
441
442
443
444
445
m/z

2-(2,4-dioxo-5-(pyridin-2-ylmethylene)thiazolidin-3-yl)-N-(4-phenylthiazol-2-yl)acetamide (HB123)

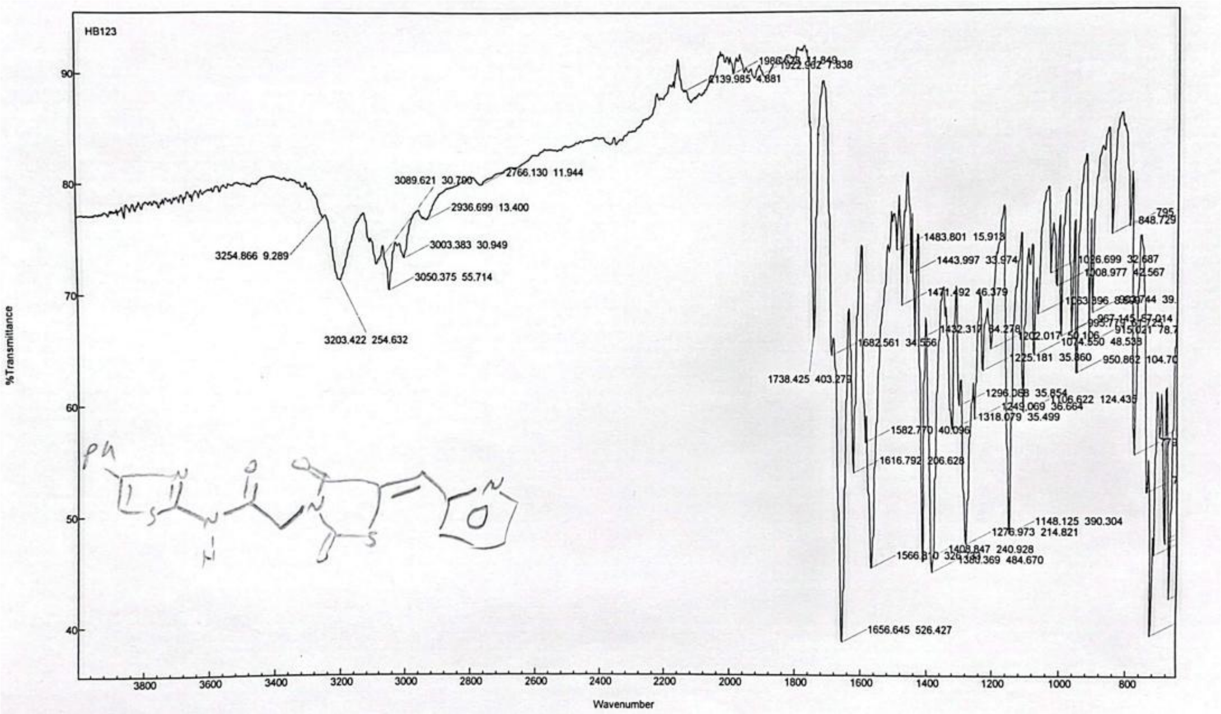

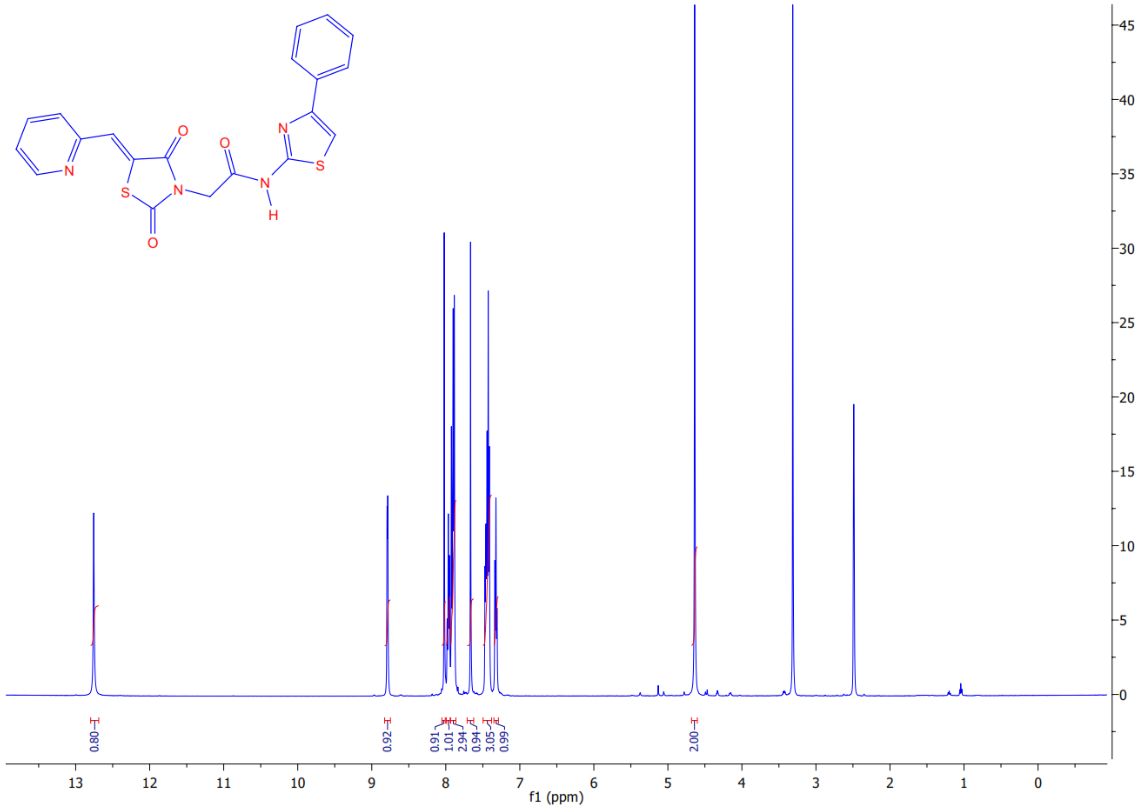

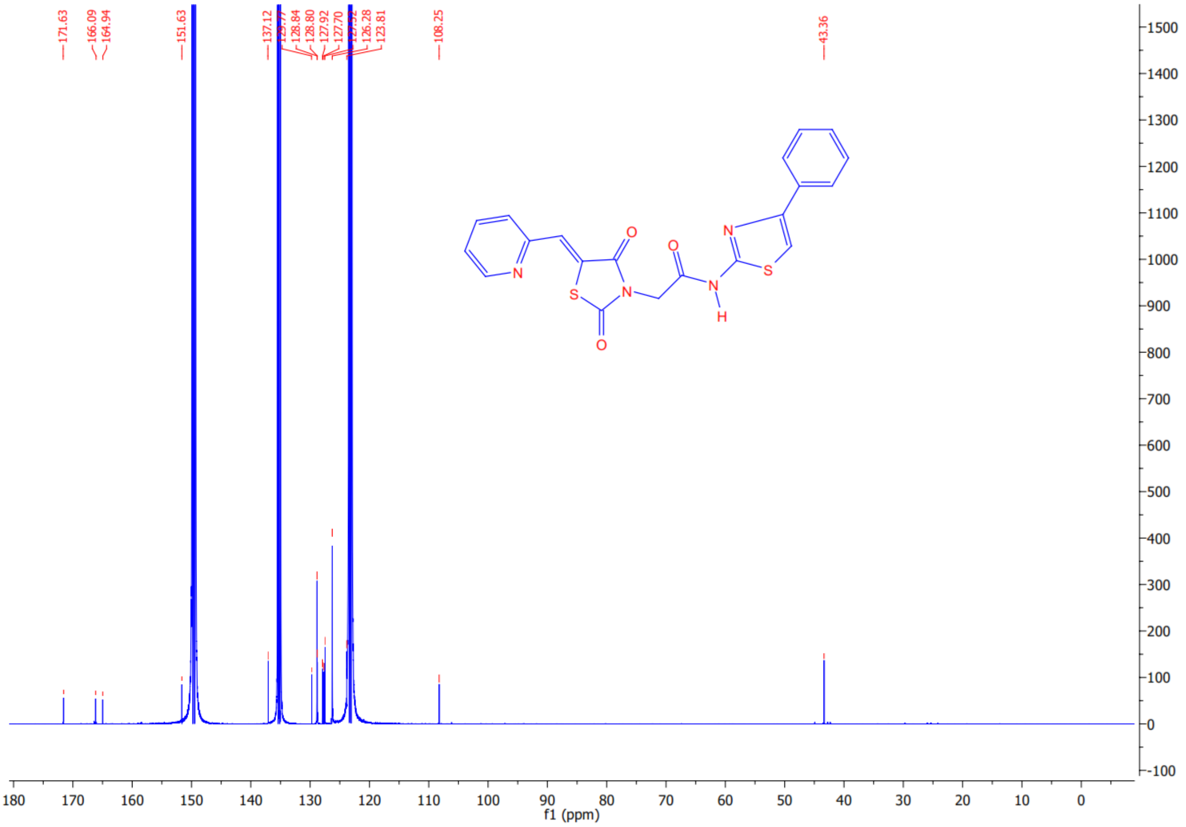
171.63
166.09
164.94
151.63
137.12
128.84
128.80
127.92
127.70
126.28
123.81
108.25
43.36
f1 (ppm)

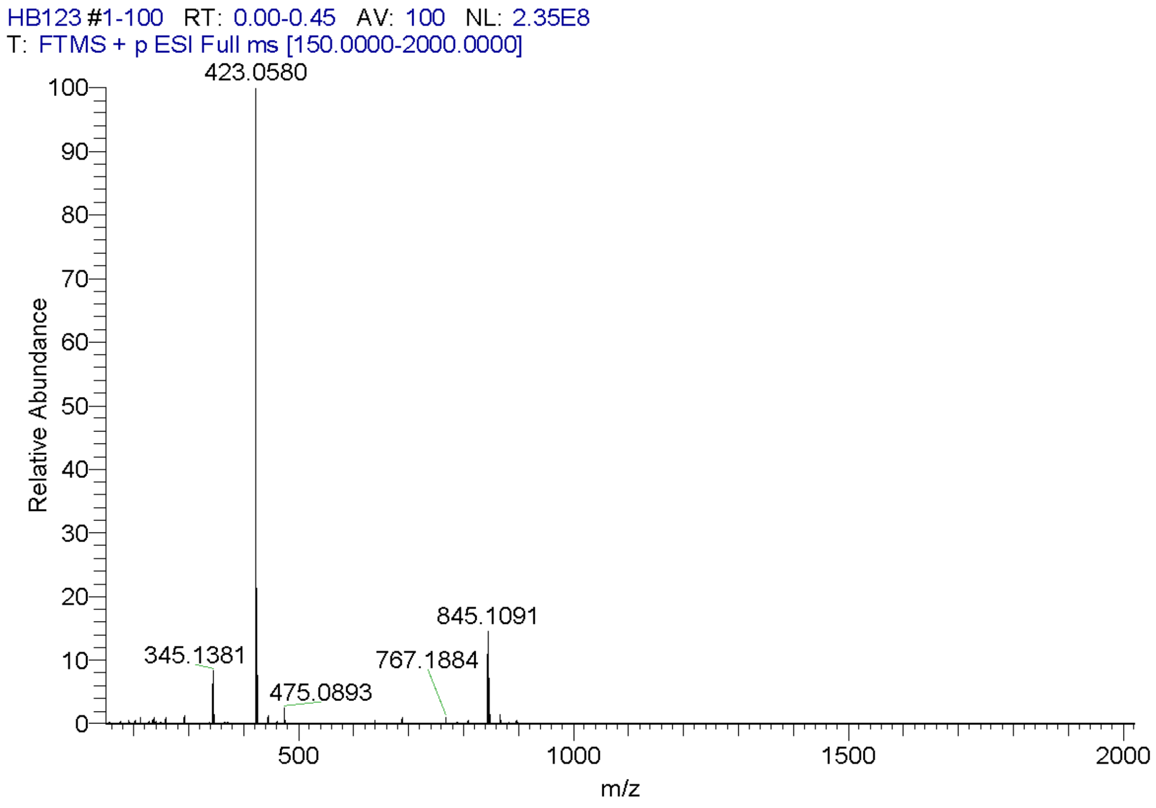
HB123 #1-100 RT: 0.00-0.45 AV: 100 NL: 2.35E8
T: FTMS + p ESI Full ms [150.0000-2000.0000]
423.0580
845.1091
345.1381
767.1884
475.0893
Relative Abundance
m/z

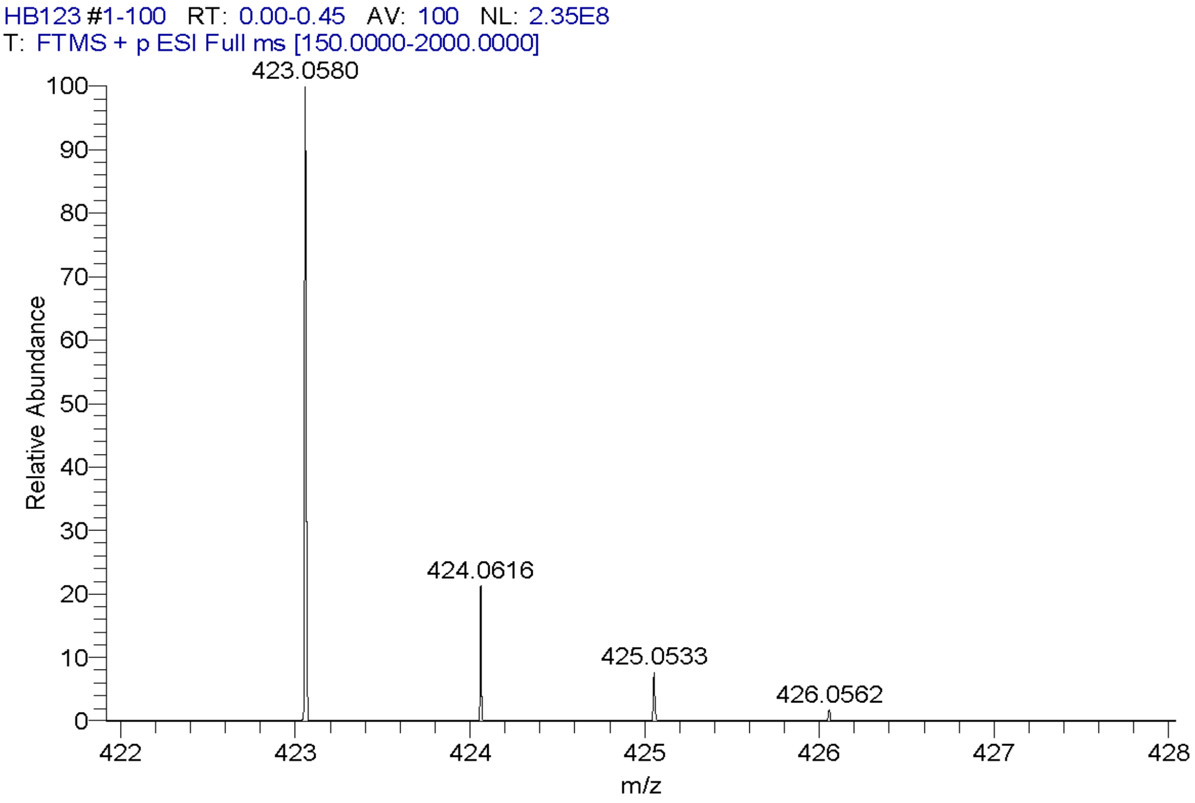
HB123 #1-100 RT: 0.00-0.45 AV: 100 NL: 2.35E8
T: FTMS + p ESI Full ms [150.0000-2000.0000]
423.0580
424.0616
425.0533
426.0562
Relative Abundance
m/z

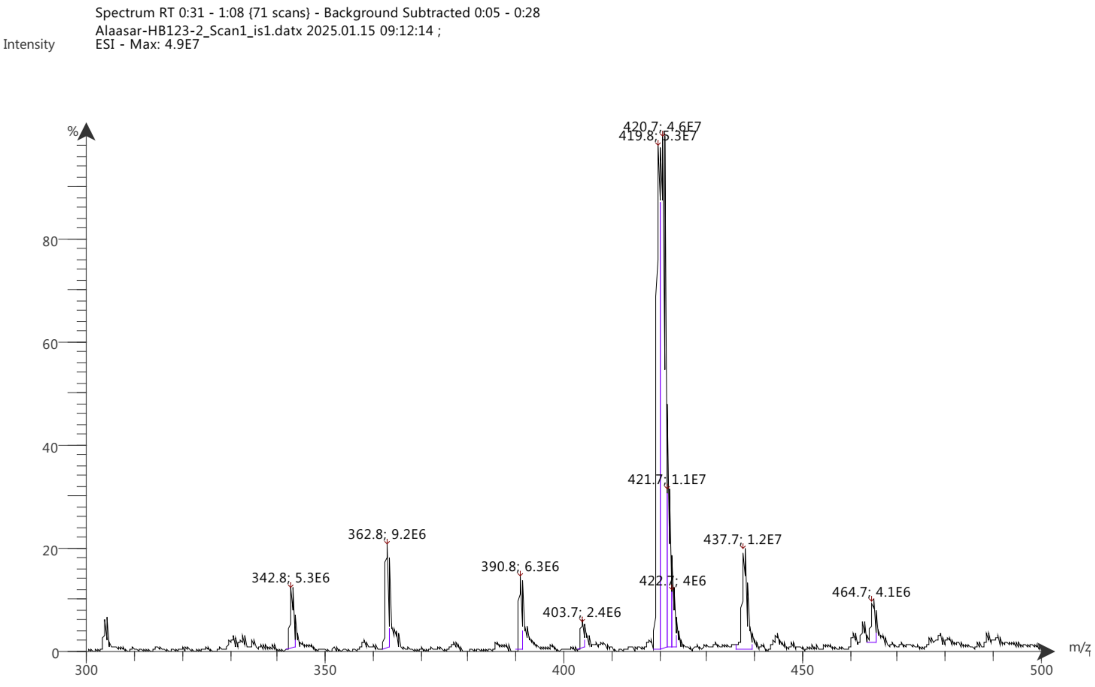
Spectrum RT 0:31 - 1:08 (71 scans) - Background Subtracted 0:05 - 0:28
Alaasar-HB123-2_Scan1_is1.datx 2025.01.15 09:12:14 ;
ESI - Max: 4.9E7
Intensity
420.7; 4.6E7
419.8; 5.3E7
421.7; 1.1E7
437.7; 1.2E7
362.8; 9.2E6
390.8; 6.3E6
342.8; 5.3E6
422.7; 4E6
464.7; 4.1E6
403.7; 2.4E6
m/z

Biological Data

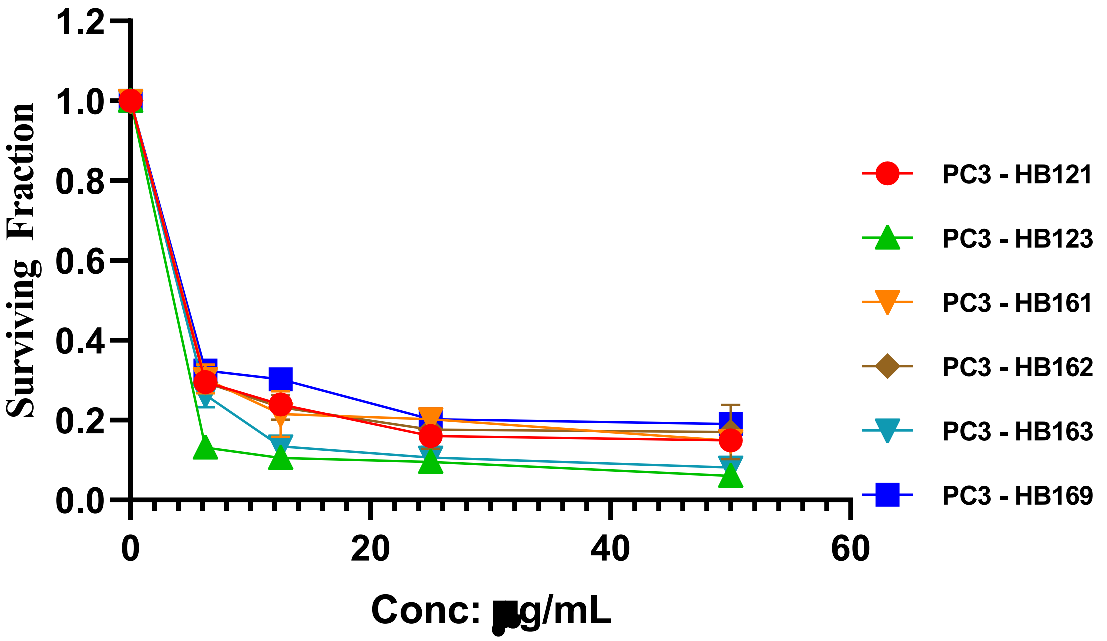

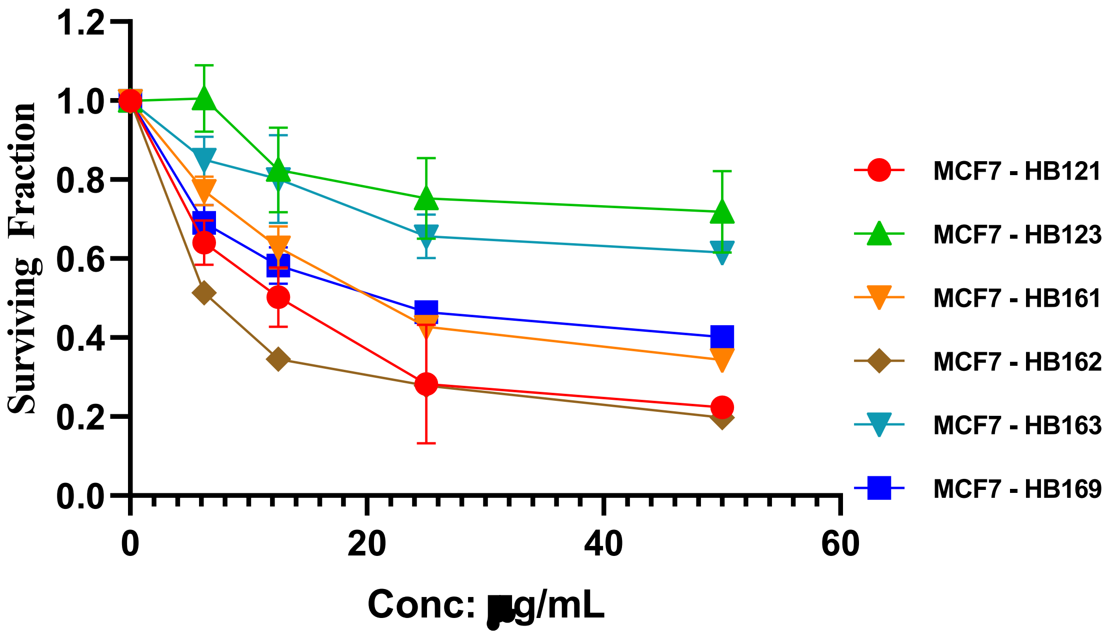

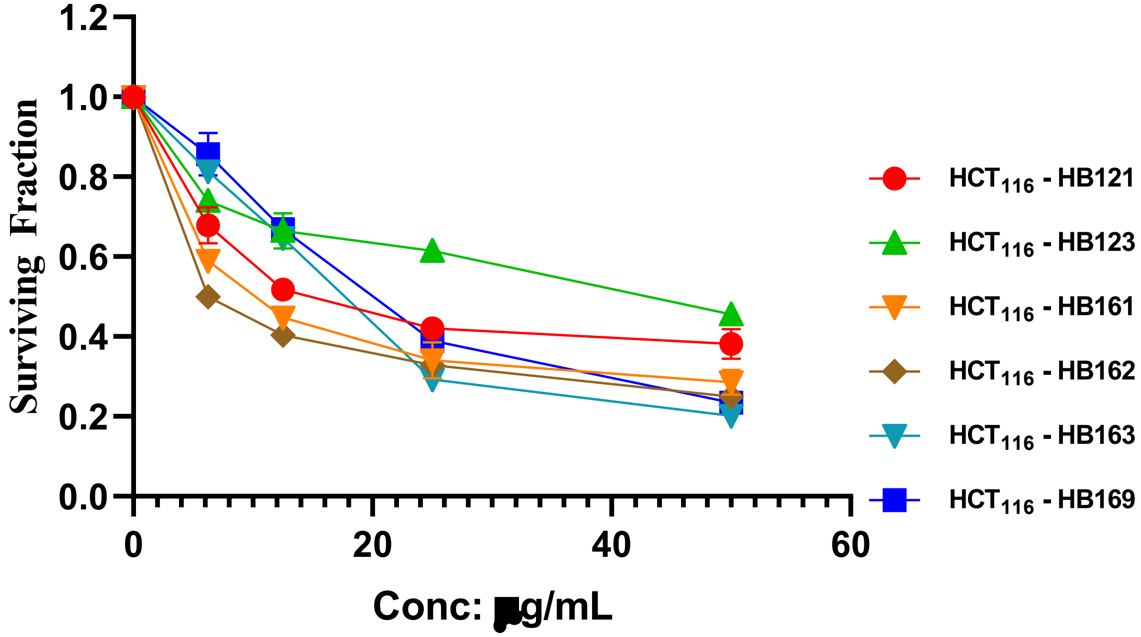

Detailed representations of the IC_{50} calculation curves of each tested cell line with the evaluated new analogues (**HB121–HB169**).

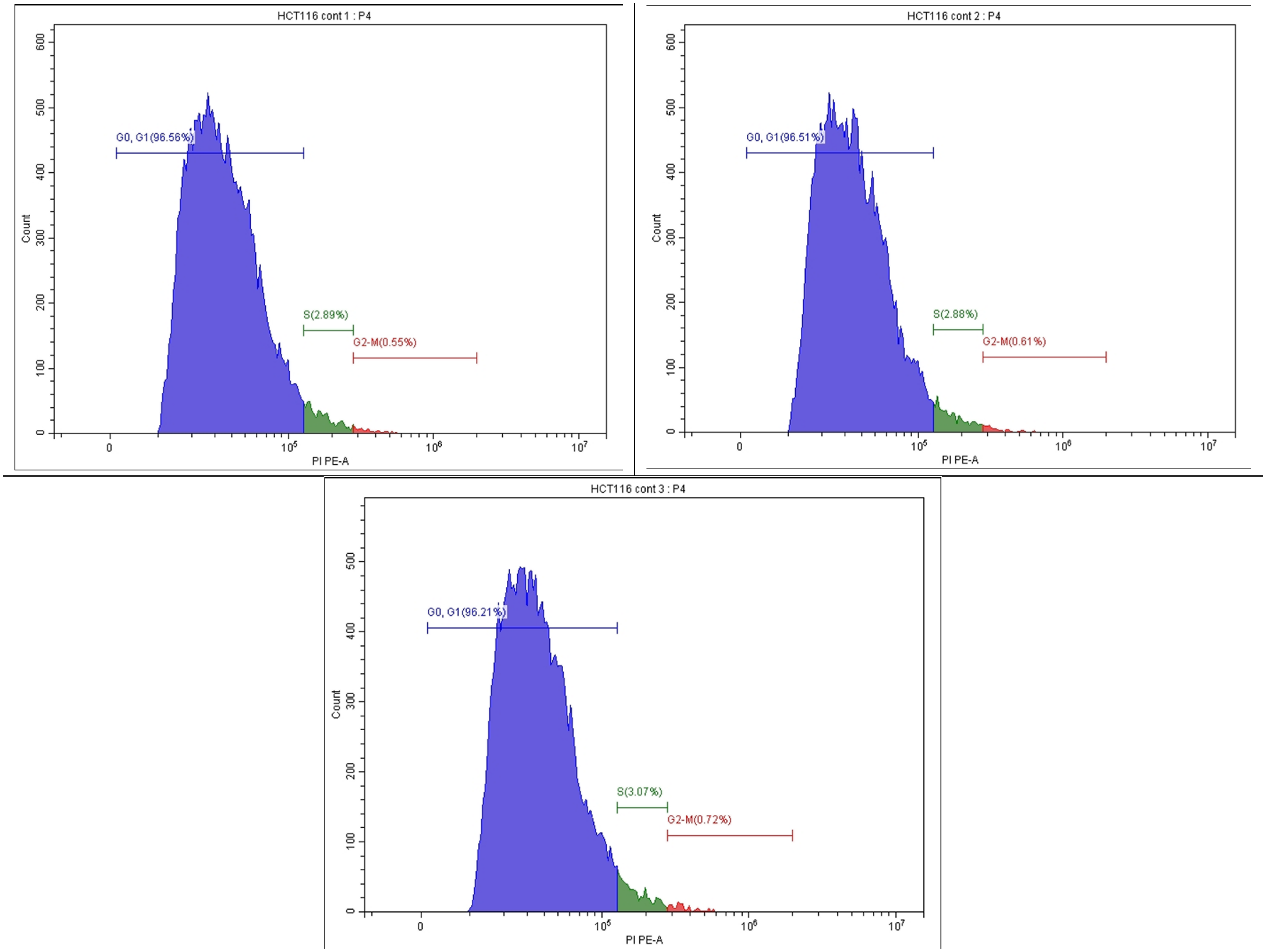

Figure S1. The triplicate experiments of the cell cycle analysis histogram of the untreated control HCT_{116} cancer cell line.

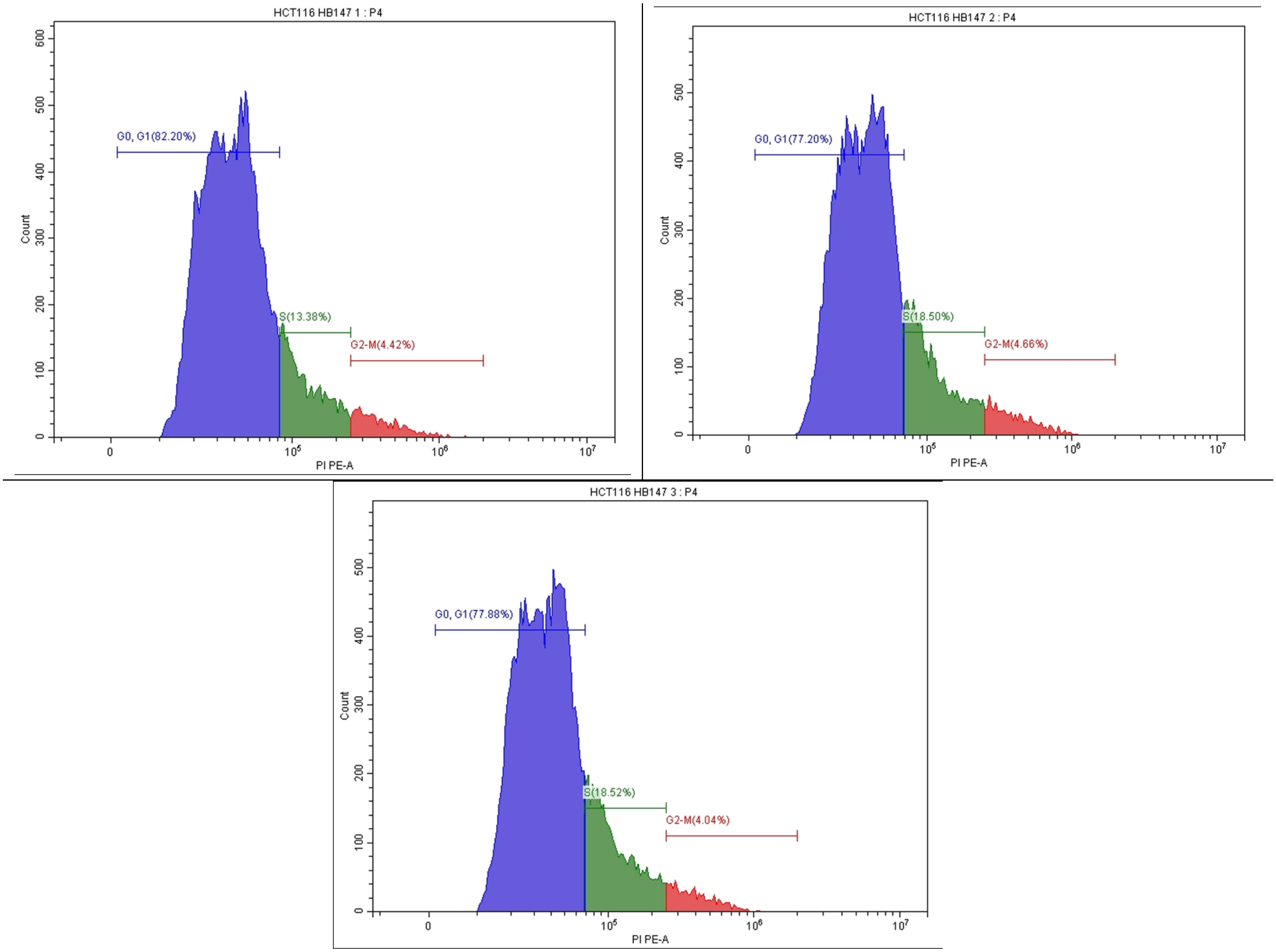

Figure S2. The triplicate experiments of the cell cycle analysis histogram of the **HB147**-treated HCT_{116} cancer cell line.

ADMET and Physicochemical Studies

HB121

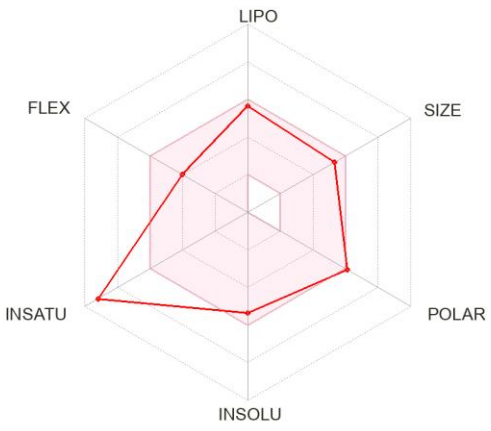

HB123

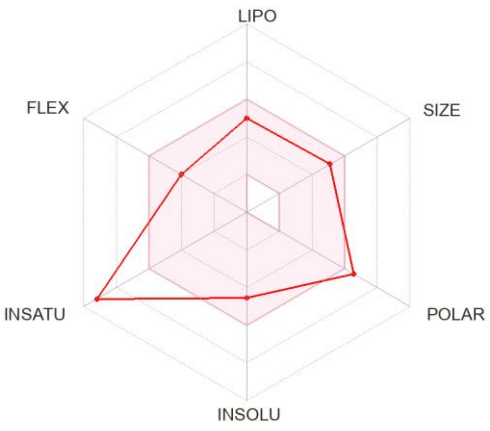

HB161

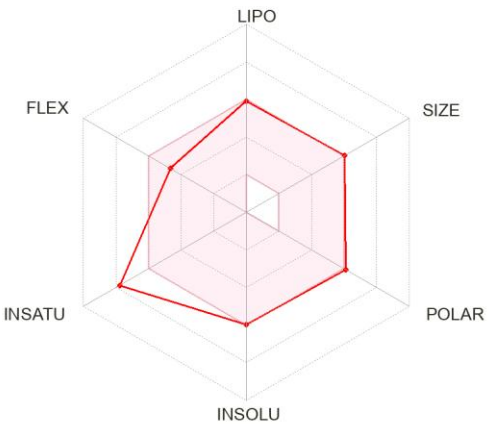

HB162

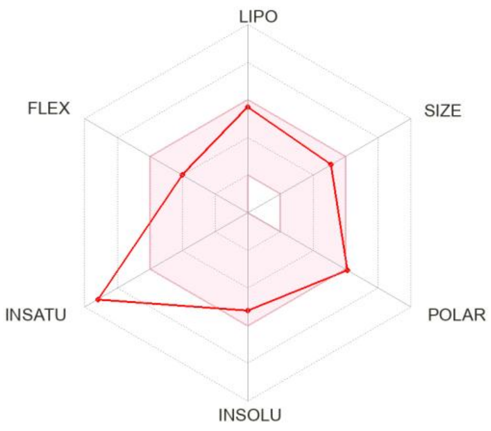

HB163

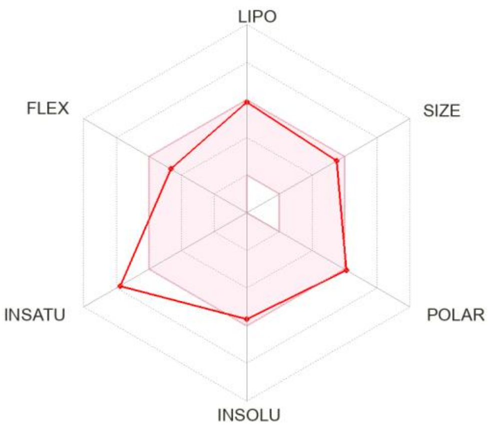

HB169

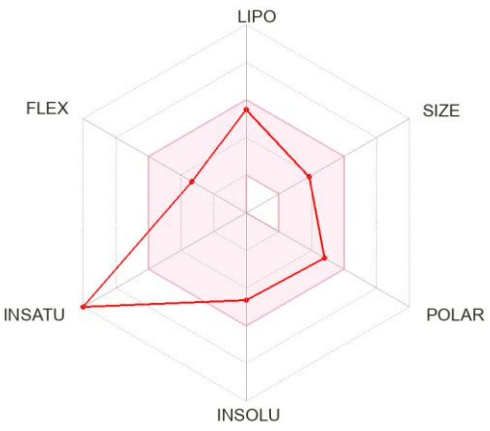

Doxorubicin

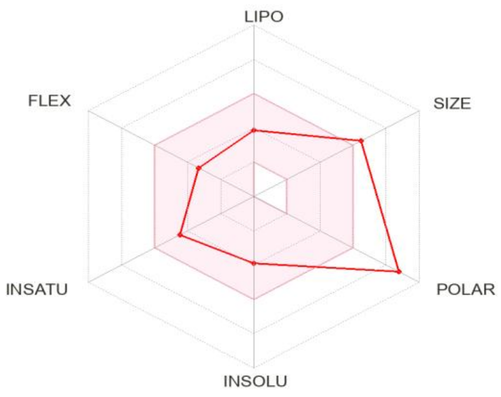

Figure S3. Radar bioavailability for the studied compounds (**5c** and **5f**), along with doxorubicin, in which the area in pink displays the specific property optimal range. (LIPO = lipophilicity

expressed as XLOGP3 (range from -0.7 to 5.0). SIZE = size expressed as molecular weight (range from 150 g/mol to 500 g/mol). POLAR = polarity expressed as TPSA (topological polar surface area) (range from 20 Å^2 to 130 Å^2). INSOLU = water insolubility by log S (ESOL) (range from -6 to 0). INSATU = insaturation expressed as for each carbon fraction in sp3 hybridization (range from 0.25 to 1). FLEX = flexibility, expressed as the number of rotatable bonds (range from 0 to 9).

References

1. E. O. Olawode, R. Tandlich, E. Prinsloo, M. Isaacs, H. Hoppe, R. Seldon, D. F. Warner, V. Steenkamp and P. T. Kaye, *ARKIVOC*, 2018, **2018**, 110-118.
2. G. Yan, L. Hao, Y. Niu, W. Huang, W. Wang, F. Xu, L. Liang, C. Wang, H. Jin and P. Xu, *European journal of medicinal chemistry*, 2017, **137**, 462-475.
3. C. Nitsche, V. N. Schreier, M. A. Behnam, A. Kumar, R. Bartenschlager and C. D. Klein, *Journal of Medicinal Chemistry*, 2013, **56**, 8389-8403.
